# Frequent intra- and inter-species introgression shapes the landscape of genetic variation in bread wheat

**DOI:** 10.1186/s13059-019-1744-x

**Published:** 2019-07-12

**Authors:** Hong Cheng, Jing Liu, Jia Wen, Xiaojun Nie, Luohao Xu, Ningbo Chen, Zhongxing Li, Qilin Wang, Zhuqing Zheng, Ming Li, Licao Cui, Zihua Liu, Jianxin Bian, Zhonghua Wang, Shengbao Xu, Qin Yang, Rudi Appels, Dejun Han, Weining Song, Qixin Sun, Yu Jiang

**Affiliations:** 1 State Key Laboratory of Crop Stress Biology in Arid Areas, College of Agronomy, Northwest A&F University, Yangling, 712100 China; 2 College of Animal Science and Technology, Northwest A&F University, Yangling, 712100 China; 3 Department of Molecular Evolution and Development, University of Vienna, Vienna, Austria; 4 State Key Laboratory of Crop Stress Biology for Arid Areas, College of Horticulture, Northwest A&F University, Yangling, 712100 China; 5 AgriBio, Centre for AgriBioscience, Department of Economic Development, Jobs, Transport, and Resources, La Trobe University, 5 Ring Road, Bundoora, VIC 3083 Australia; 6 College of Agronomy and Biotechnology, China Agricultural University, Beijing, 100193 China

**Keywords:** Bread wheat, Genetic diversity, Haplotype, Introgression, Selection

## Abstract

**Background:**

Bread wheat is one of the most important and broadly studied crops. However, due to the complexity of its genome and incomplete genome collection of wild populations, the bread wheat genome landscape and domestication history remain elusive.

**Results:**

By investigating the whole-genome resequencing data of 93 accessions from worldwide populations of bread wheat and its diploid and tetraploid progenitors, together with 90 published exome-capture data, we find that the B subgenome has more variations than A and D subgenomes, including SNPs and deletions. Population genetics analyses support a monophyletic origin of domesticated wheat from wild emmer in northern Levant, with substantial introgressed genomic fragments from southern Levant. Southern Levant contributes more than 676 Mb in AB subgenomes and enriched in the pericentromeric regions. The AB subgenome introgression happens at the early stage of wheat speciation and partially contributes to their greater genetic diversity. Furthermore, we detect massive alien introgressions that originated from distant species through natural and artificial hybridizations, resulting in the reintroduction of ~ 709 Mb and ~ 1577 Mb sequences into bread wheat landraces and varieties, respectively. A large fraction of these intra- and inter-introgression fragments are associated with quantitative trait loci of important traits, and selection events are also identified.

**Conclusion:**

We reveal the significance of multiple introgressions from distant wild populations and alien species in shaping the genetic components of bread wheat, and provide important resources and new perspectives for future wheat breeding.

**Electronic supplementary material:**

The online version of this article (10.1186/s13059-019-1744-x) contains supplementary material, which is available to authorized users.

## Background

The domestication of plants and animals near Fertile Crescent has shown great impacts on modern human civilization [1]. Among them, bread wheat (*Triticum aestivum*, AABBDD) is one of the most striking and indispensable staple crops, which now accounts for 17% of the total cultivated area in the world, and provides ~ 20% of calories global populations consumed. Given the impacts of climate and environmental changes and the growing demand for food, new wheat varieties with improved yields, enhanced resistance to diseases, and better fitness are needed. Thus, an in-depth investigation into the wheat genome and characterization of its genetic variations is extremely necessary.

Bread wheat originated from hybridization between cultivated tetraploid emmer wheat (*Triticum turgidum*. L, AABB) and wild diploid *Aegilops tauschii* (DD) approximately 8000–10,000 years ago [2, 3]. Its genomic variation was shaped by initial bottlenecks related to polyploid speciation and domestication, the subsequent reintroduction of variation mediated by natural and artificial hybridization, as well as selection during wheat improvement process [2–4]. Although extensive studies have been performed to characterize the genetic diversity, origin, and domestication of wheat [5–8], the phylogenetic history of A, B, and D ancestral genomes diverging a few million years ago has not been well documented recently [9, 10]. There are two independent evolutionary processes: (1) the initial domestication of wild emmer (*T. turgidum* ssp. *dicoccoides*, AABB) and the subsequent evolution of domesticated emmer wheat (*T. turgidum* ssp. *dicoccum*, AABB) to improved durum wheat (*T. turgidum* ssp. *durum*, AABB) and (2) hybridization of tetraploid emmer wheat and *Ae. tauschii* to form hexaploid bread wheat. However, the debate about whether tetraploid emmer wheat was domesticated independently in southeastern Turkey (northern Levant) and southern Levant or has a monophyletic origin combined with the subsequent hybridization among the population is ongoing among researchers [4, 11–14]. Detailed analyses including diverse wild and domesticated populations with a high density of genetic markers are needed to resolve this disagreement.

Based on the limited number of genetic markers such as restriction fragment length polymorphism (RFLP), simple sequence repeat (SSR), single nucleotide polymorphisms (SNPs), etc., previous studies estimated that about 58% of the variants observed in wild emmer variations were reserved in bread wheat, while only 7% in *Ae. tauschii* were reserved in bread wheat [4, 15]. The variation patterns of bread wheat are asymmetric that A and B genomes contained about 2.5 times more SNPs than D genome [6, 8], probably due to the smaller population size of the D genome ancestors for wheat formation [16]. At the same time, large fragment deletions and duplications (copy number variations (CNVs)), a major feature during the wheat diploidization, have not been detailedly investigated. Despite that, massive studies have tried to uncover the genetic relationships of geographical bread wheat populations. A previous study using worldwide collections of bread wheat accessions suggested that most landraces clustered away from varieties, and landrace accessions were clustered by geographic localities [5]. The landraces of bread wheat generally display a much higher level of genetic diversity than elite varieties. This is likely caused by further improvement and extensive utilization of certain lines for breeding, e.g., the semi-dwarf photoperiod-insensitive lines which formed the basis of the “Green Revolution” [17]. Many studies have shown that introgressions from wild relatives contribute valuable variations to wheat genomes [4, 18]. However, wild relatives, which play vital roles in the genetic architecture of bread wheat, have been lacking in previous researches. A more comprehensive analysis of genomic variation landscape across divergent bread wheat lines, the patterns of subpopulation divergence and the contributions of alien genomic components, is urgent.

The recently completed reference genome of bread wheat cv. Chinese Spring (IWGSC RefSeq v1.0) [19] allows us to systematically analyze the whole-genome resequencing data to underlie landscape of wheat variation. Here, we generated whole-genome resequencing data from worldwide wheat accessions and its wild progenitors and combined the massive published whole-genome sequencing and exome capture data to address the following questions: (1) How variations vary across diverse populations and subgenomes? (2) How multiple wild populations and alien introgression contribute to the genetic composition of bread wheat? and (3) How the above factors drive the evolution of bread wheat populations. Here, we provided a whole-genome variation map and reveal the evolutionary trajectory of wild and domesticated wheat, which can further promote functional studies and utilization of various alleles in wheat breeding.

## Results

### Genomic variation landscape across subgenomes

**Fig. 1:**
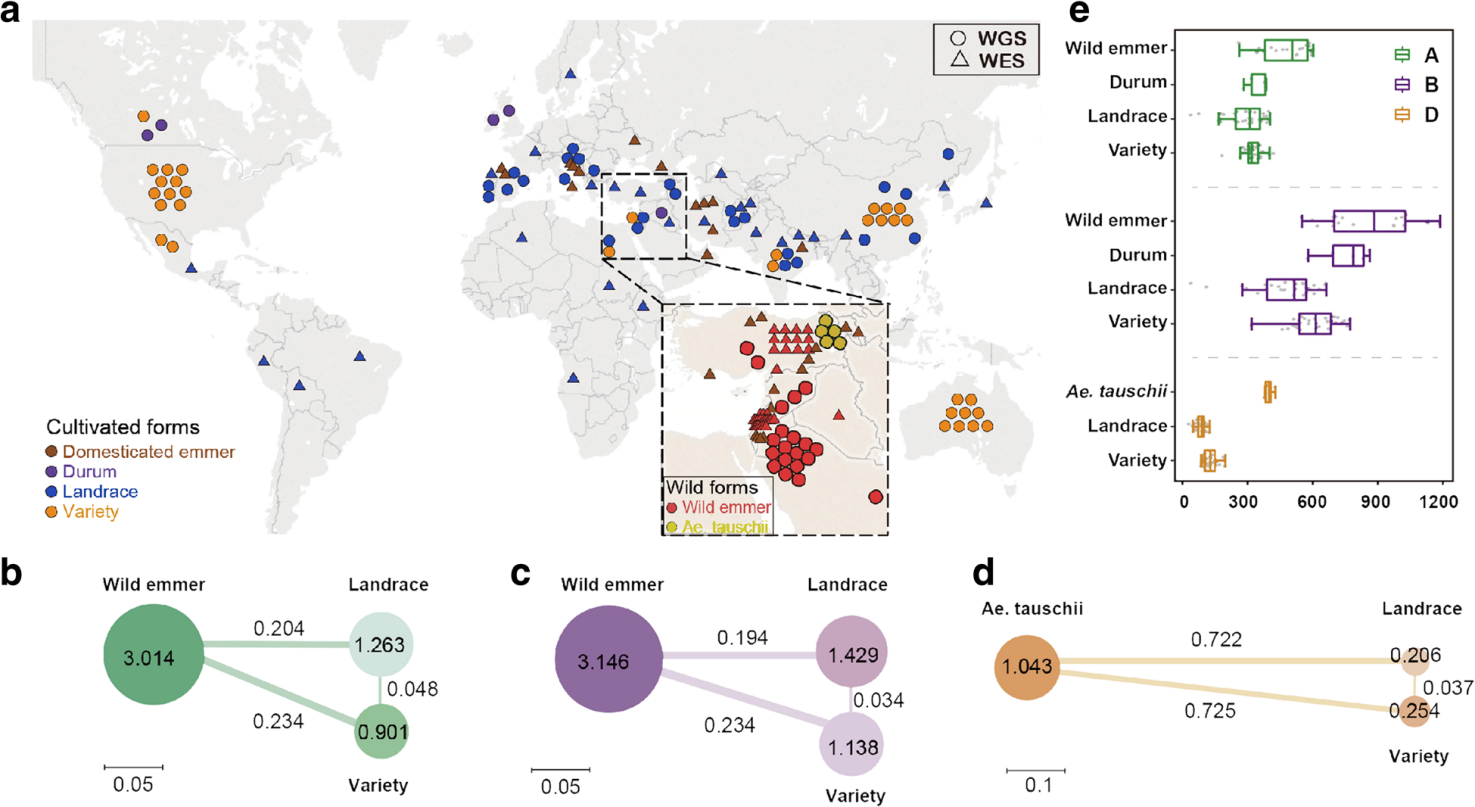
Geographic distribution and population diversity of wheat accessions. **a** Schematic geographical distribution of the collection sites for 93 whole-genome sequencing (WGS) accessions and 92 whole-exome sequencing (WES) accessions. The locations of wild progenitors of bread wheat are shown in the inset. **b**–**d** Nucleotide diversity and population divergence across the wild progenitor, landraces, and varieties among the A subgenome, B subgenome, and D subgenome. The circle size and the value in each circle represent nucleotide diversity (*π* × 10^3^), and the length of the line indicates divergence (*F*_st_) among the populations. **e** The length of the deleted CNVs in divergent populations. The deleted CNVs were identified using the IWGSC RefSeq v1.0. The B subgenome contains more deleted CNVs in length than the A and D subgenomes in the wild emmer, landrace, and variety

To construct a comprehensive map of genetic variations of bread wheat, we analyzed the resequencing data of 93 worldwide individuals, including 20 accessions of wild emmer wheat, 5 *Ae. tauschii*, 5 durum wheat, 29 hexaploid landraces, and 34 hexaploid varieties, of which 75 were newly obtained in this study while the others are published [19–21] (Fig. 1a, Additional file 2: Table S1). We identified a total of 84,594,991 SNPs and 11,628,085 indels by mapping resequencing reads against the wheat reference (IWGSC RefSeq v1.0) [19] (Additional file 1: Figures S1 and S2, Additional file 2: Table S2). Among those, there are 210,013 non-synonymous SNPs and 36,904 frameshift indels that might have impacts on protein functions. The accuracy of those SNPs reaches more than 95% as estimated by using wheat 660 K SNP arrays (http://wheat.pw.usda.gov/ggpages/topics/Wheat660_SNP_array_developed_by_CAAS.pdf) (Additional file 2: Table S3). In addition, we characterized structural variations through a global analysis of copy number variations (CNVs, including deletions and duplications) in each accession. A total of 105,316 deletions and 100,509 duplications were identified, corresponding to ~ 1923 and ~ 1407 Mb genomic sequences, respectively, with the deletion sizes ranged from 4 kb to 33 Mb (Additional file 3: Tables S4, Additional file 4: Table S5, Additional file 5: Tables S6, Additional file 6: Table S7, Additional file 7: Tables S8, Additional file 8: Table S9). To retrieve the regions that are present in wild progenitor but absent in the reference genome (Chinese Spring, IWGSC RefSeq v1.0), we reconstructed a pseudo-genome comprised of the wild emmer [22] and the *Ae. tauschii* genomes [20] to conduct the read mapping. This leads to the identification of 1517 Mb sequences that have lost in the reference genome. To our knowledge, the amount of those genetic variations is larger than any previous datasets. Such large-scale genomic resources derived from worldwide populations allow us to systematically assess the dynamics of genetic variations during the long history of wheat domestication.

One of the expected evolutionary consequences of purifying selection during wheat domestication is the decline of genetic diversity near the genic region [23]. To evaluate the diversity-reducing effect of purifying selection, we investigated the genetic diversity distribution around genes. We found a slower rate of recovery of diversity with distance away from the genic region in bread wheat compared with wild emmer wheat (Additional file 1: Figure S3), suggesting a more relaxed purifying selection in hexaploid bread wheat. During the domestication process, the genetic diversity (measured by *π*) decreased dramatically from wild emmer (3.014 × 10^−3^ for A subgenome, 3.146 × 10^−3^ for B subgenome) to the domesticated hexaploid landraces (1.263 × 10^−3^ for A subgenome, 1.429 × 10^−3^ for B subgenome), by more than twofolds (Fig. 1b, c; Additional file 1: Figures S4, S5, and S6). For the D subgenome, we observed a more extensive loss of genetic diversity (Fig. 1d). The rapid loss of genetic diversity was probably due to the severe bottlenecks associated with the domestication and polyploidization events of allohexaploid bread wheat. The B subgenome harbors higher genetic diversity than the A subgenome in wild emmer, as well as in landrace and variety even after strong selection (Fig. 1b, c), suggesting that the pattern was inherited from wild progenitors. Interestingly, unlike the A and B subgenomes, the D subgenome of the improved varieties showed slightly higher diversity than that of the landraces (Fig. 1d). This can be in part explained by introgressive hybridization with its wild relatives during modern breeding processes [24, 25].

The asymmetric variation pattern among the subgenomes is also uncovered by the analysis of deletions. While the difference of SNP density between A and B subgenomes is negligible, deletions showed a much larger difference between A and B (Fig. 1e), implying the initial diploidization in wheat genome may come with substantial gene loss. This phenomenon is also suggestive of faster evolutionary rate and/or more relaxed purifying selection on B subgenome relative to A subgenome. In a word, the differences in both genetic diversity and deletions manifested as early as in wild emmer suggest the ancestral differential evolutionary trajectories of subgenomes.

### Genetic differentiation among and within populations

**Fig. 2:**
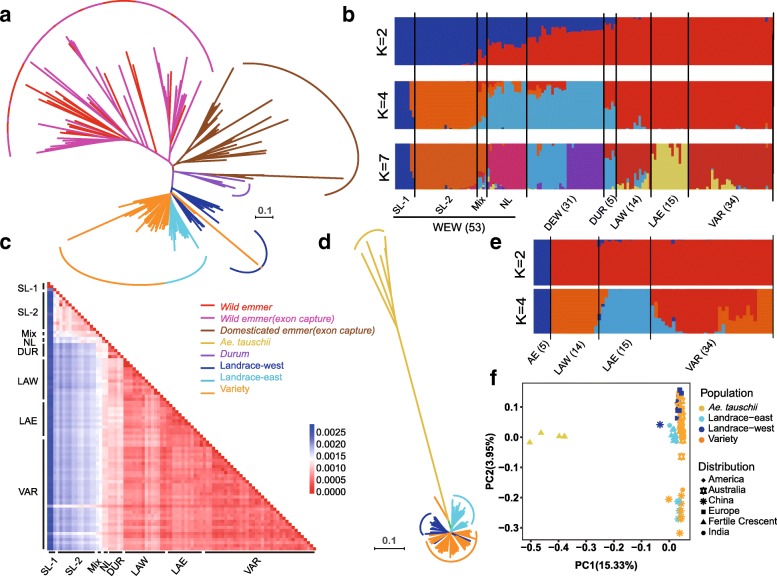
Phylogenetic relationships and population structures. **a** The NJ tree was built with 1000 bootstraps using a total of 312,952 overlapping SNPs with exome capture data. Branch colors reflect different populations. Accessions are arranged according to the populations and their geographic locations. **b** Population structure of the 152 wheat accessions on AB subgenomes, including wild emmer (WEW), domesticated emmer (DEW), durum (DUM), landrace-west (LAW), landrace-east (LAE), variety (VAR). **c** Nucleotide distance (*dxy*) between each pairwise of wheat accessions by whole-genome resequencing. **d** NJ tree of 68 whole-genome resequencing accessions on the D subgenome. The tree was built using the 1,879,923 SNPs located in the genic region. **e** Population structure of the 68 wheat accessions on the D subgenome. **f** PCA plots of the first two components of 68 wheat accessions. The color and shape of dots separately indicate the population and location

To investigate the population differentiation of wheat accessions across the globe, we used the SNPs that were shared in our datasets and previously published exome capture data [6, 22] (Additional file 2: Table S10) to perform phylogenetic tree and principal component analysis (PCA). The results showed a clear separation of wild emmer, domesticated tetraploid, and hexaploid wheat accessions, suggesting a single domestication event of wheat (Fig. 2a, Additional file 1: Figures S7, S8, and S9). Wild emmer wheat from southern and northern Levant (SL and NL) clustered into two clades, corroborating previous findings [4, 12, 22]. Moreover, accessions from SL grouped into two main clades (denoted as SL-1 and SL-2) (Fig. 2a). This differentiation pattern of SL-1, SL-2, and NL populations of wild emmer was consistent with the ADMIXTURE analysis despite a few admixed samples (Fig. 2b, Additional file 1: Figure S10). To evaluate the genetic relationship between wild emmer populations and bread wheat, we calculated the nucleotide distance (*dxy*, an index to evaluate the nucleotide distance) between each pair of accessions [26]. The result suggested that all lines of present-day bread wheat as well as domesticated emmer and durum were closer to NL population (Fig. 2b, c; Additional file 1: Figure S11), consistent with previous depiction that domesticated emmer were more similar to the wild emmer in southeast Turkey [22, 27], while SL-1 population was genetically distant to bread wheat (Fig. 2b, c; Additional file 1: Figure S11). This suggested that the NL lineage of wild emmer is likely the donor of AABB genome of bread wheat, domesticated emmer, and durum. For D subgenome, *Ae. tauschii* similarly showed a clear separation from bread wheat (Fig. 2d), corroborating the results of ADMIXTURE (*K* = 2) (Fig. 2e; Additional file 1: Figure S10) and PCA analysis (Additional file 1: Figure S9). Interestingly, the PCA result showed that most Chinese bread wheat isolated from the others for the second principal component (Fig. 2f). This is probably related to the fact that introgressions from *Ae. tauschii* did not occur in the Far East, such as China, while continued in the west [16].

The analyses of population structure and genetic distance among populations also revealed a dichotomous pattern of population divergence within landrace accessions of bread wheat (Fig. 2b, e; Additional file 1: Figures S10 and S12). Two distinct groups separated geographically correspond to European and Asian areas (Additional file 1: Figure S13), combined with an extended dataset of 26 worldwide landraces generated by exome capture [6] (Additional file 1: Figure S13). For convenience, we defined these two clusters as landrace-west and landrace-east (Additional file 2: Table S11). The varieties, in contrast, show no internal segregations in genetic structure (Fig. 2b), likely due to the extensive germplasm exchanges during the process of improvement in modern wheat varieties [28]. Similarly, population differentiation statistics (*F*_st_) also reveal substantial divergence between western and eastern landrace populations (Additional file 1: Figure S14). The average *F*_st_ between western and eastern populations are 0.159, 0.134, and 0.06 for the A, B, and D subgenome, respectively, significantly higher than the average *F*_st_ between landrace and varieties (0.048, 0.034, and 0.037 for the A, B, and D subgenome, respectively) (Fig. 1b–d, Additional file 1: Figure S15), demonstrating greater population differentiation between landrace-west and landrace-east than that between landrace and variety of bread wheat.

### Contributions of diverse haplotypes from various wild emmer populations

**Fig. 3:**
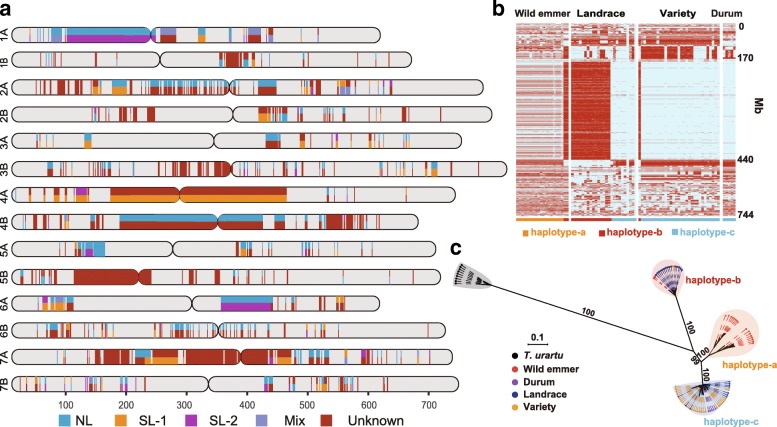
Segmental ancestry inference of present-day bread wheat. **a** The distributions of long haploblocks with haplotype diversity along the AB subgenomes of bread wheat. The origins of haplotypes are shown in different colors. Segmental ancestry derived from the four populations of wild emmer, NL, SL-1, SL-2, and mix. **b** Haplotype patterns of chromosome 4A in diverse populations. Each column is an accession, each row is a phased haplotype. The haplotypes were constructed for each accession using all the SNPs on chromosome 4A. Alleles that are identical to or different from the ones in the IWGSC RefSeq v1.0 reference genome are indicated by blue and red, respectively. **c** ML tree of the longest haplotypes on chromosome 4A from ~ 170 to 440 Mb. All the accessions clustered into three groups corresponding to three distinct haplotypes in **b**

It has been suggested that frequent gene flow between wild and domesticated emmer occurred across broad natural habitat of wild emmer [4]. To investigate the relative contributions of various populations of wild emmer partitioned above to genetic pools of bread wheat, we sought to identify the haploblocks in bread wheat using the whole-genome resequencing data. In total, we identified of 438 haploblocks (1914 Mb) longer than 1 Mb with haplotype diversity, of which 41 haploblocks spanned over 10 Mb, and 5 were more than 100 Mb encompassing the defined centromeric regions [19] (Fig. 3a, Additional file 9: Table S12). The substantially reduced recombination rates in the centromeric regions are probably the reason why these haplotypes have maintained for such a prolonged period. SNPs on these haploblocks were used to examine the patterns of haplotype sharing and to trace back the putative origins of distinct haplotypes (Additional file 1: Figure S16). We found that phylogenetic relationships differ among different regions (Additional file 1: Figure S16). A general pattern is that the bread wheat accessions with distinct haplotypes are always grouped with different wild emmer accessions indicating their various origins (Additional file 1: Figure S16). Among the 438 haploblocks, we found 224 are derived from the NL population with a total length of 1164 Mb. Surprisingly, we identified 94 out of those haploblocks (94/438) were likely derived from the SL-1 population, the most distant population to bread wheat, and they covered ~ 676 Mb genomic sequence. Meanwhile, we found a large portion of haplotypes in bread wheat were not present in any of the 53 wild emmer accession in 346 haploblocks, which could be due to the introgression from unknown or extinct emmer populations (Fig. 3a, Additional file 1: Figure S16).

To estimate the divergence time of the different haplotypes, we used the longest haploblock on chromosome 4A from position ~ 170 to ~ 440 Mb (Fig. 3b, c; Additional file 1: Figure S17), as it contains the largest number of informative variants. There appears to be three distinct groups of haplotypes in this region, named haplotype-a, haplotype-b, and haplotype-c (Fig. 3b, Additional file 1: Figure S17). After applying the isolation-with-migration model embedded in CoalHMM [29] in 10-Mb windows with 5-Mb step size, we estimated that the most differentiated haplotype-b and haplotype-c diverged on average ~ 0.46 million years ago, haplotype-a and haplotype-b diverged ~ 0.40 million years ago, and haplotype-a and haplotype-c diverged ~ 0.20 million years ago (Additional file 1: Figure S18), largely within the time frame of wild emmer origin [30, 31]. The large divergent time among the three haplotypes is more likely due to the different wild emmer origins rather than the scenario of a single origin during wheat domestication followed by subsequent divergence. This remarkably long haplotype block was likely maintained by low recombination rate near the centromere and/or selective pressure, and the large divergence between the two haplotypes may further lead to recombination suppression. The multiple sources of inter-species introgression from various wild populations have probably contributed to the genetic diversity of bread wheat, as demonstrated by the significantly higher genetic diversity exhibited in the regions with multiple haplotypes than those with a single haplotype (Additional file 1: Figure S19).

### Widespread alien introgression into the wheat genome

**Fig. 4:**
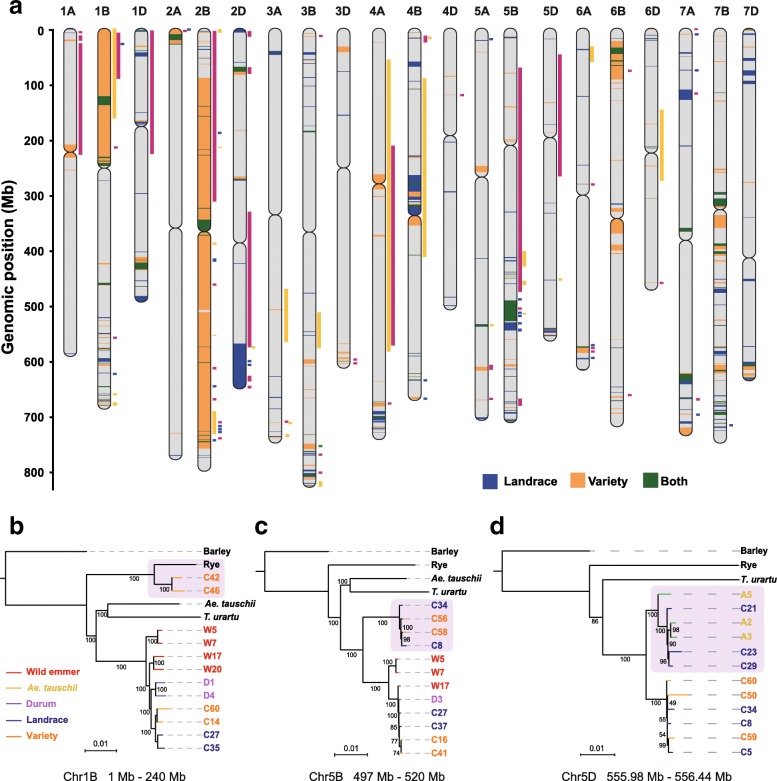
Genome-wide introgressions from wild relatives into landraces and varieties. **a** Map of the lengths and distributions of all the putative introgressed segments on 21 chromosomes. Introgression regions in different populations are distinguished by the color: blue for landrace, orange for variety, and green for the introgressed segments shared between landrace and variety. The length of these columns indicates the actual length of the introgression segments. Previously mapped QTLs overlapped with introgression regions are indicated to the right side of the chromosomes by colored rectangles, magenta for QTLs related to yield, blue for QTLs associated with disease resistance, and yellow for QTLs related to development. The QTLs indicate references and confidence intervals are provided in Additional file 4: Table S9. **b** ML tree of the 1B/1R region on the chromosome 1B of the IWGSC RefSeq 1.0 from 0 to approximately 240 Mb using sequences of each individual. C42 and C46 clustered with rye. **c** ML tree of the 23 Mb introgression fragment on chromosome 5B. Several bread wheat accessions clustered in a clade, segregating from all wild emmer accessions. **d** ML tree of a 0.45-Mb fragment on chromosome 5D introgressed from *Ae. tauschii*. This fragment was shared in six landrace accessions. Trees constructed by all accessions are provided in Additional file 1: Figures S21, S29, and S31

Apart from the introduction of genetic variation via introgression from various populations of tetraploid ancestors, introgression from alien germplasm is also an important source of exotic variations for bread wheat [32–35], such as 1B1R translocation line [36, 37]. The combined evidences from deletions and SNP density were used to identify genome-wide signals of introgression. We first tested our method for a known case (the 1BS loci) of introgression in two Chinese varieties (C42 and C46) which was also independently supported by our analyses of maximum likelihood tree and *dxy* distance (Fig. 4b, Additional file 1: Figure S20, Additional file 2: Figures S21 and S22). A large deletion region was detected in these two accessions and precisely corresponds to the 1BS loci because of the low mappability of rye sequences to the wheat genome (Additional file 2: Figure S23). And the mapped reads in this region showed excessive mismatch sites (Additional file 2: Figure S24), which has been used to identify introgression in wheat [35]. Thus, we considered large segmental deletions and exceptionally high SNP density as reliable signals of inter-species introgression. Subsequent phylogenetic clustering analysis was used for verification.

Using this method, we detected introgressions in the genomes of all 63 bread wheat accessions (Fig. 4a). A total of ~ 709 Mb and ~ 1577 Mb introgressive regions in landraces and varieties were identified, respectively, with 304 Mb shared (Fig. 4a). The average length of introgressive segments per accession is ~ 59.6 Mb (Additional file 2: Figure S25). It is perhaps not surprising that varieties had much more introgressive segments than landraces, given the intense efforts of hybridization breeding in varieties. But the extensive introgressive segments found in landraces suggest that natural introgression has occurred before modern improvement. Interestingly, the total length of the putative introgression regions per accession on the B subgenome (~ 1483 Mb) was larger than those of the A (~ 259.8 Mb) and D (~ 281.7 Mb) subgenomes (Additional file 2: Figure S26), suggesting faster evolutionary rate of the B subgenome, a pattern that is also revealed by deletion analysis. Besides, to recover the alien introgression in Chinese Spring, we also compared the reference genome (IWGSC RefSeq v1.0) with the sequence of progenitors, wild emmer [22], and the *Ae. tauschii* reference genomes [20], using the flanking sequence of the deletions in Chinese Spring. A total of 53 introgressive segments with spanning ~ 17.1 Mb in Chinese Spring are determined by genomic synteny analysis (Additional file 2: Figure S27).

Four introgressive haploblocks of 4, 8, 9, and 48 Mb with a 35-fold increased SNP density on chromosome 2D, which were identified through a previous comparative sequence analysis [35], were also detected in our resequencing data (Additional file 2: Figure S28). Two of these introgressions on the long arm (Additional file 2: Figure S28) seem to be introduced through one single hybridization, as a landrace accession of bread wheat (C10) showed continuous elevated SNP density from ~ 570 Mb to the end of chromosome 2D, completely connecting these two introgression segments. This intact introgression fragment was then broken by recombination in some other accessions, e.g., C12 and C13 (Additional file 2: Figure S28). Using the regions with mapped reads, we further verified alien introgressions by constructing a phylogenetic tree together with sequences of potential donors, including barley (*Hordeum vulgare*) [38], rye (*Secale cereale*) [39], *T. urartu* [40], wild emmer, and *Ae. tauschii*. Though most of the introgressions cannot be assigned with reliable origins, the introgressed accessions showed great genetic distance with the other normal accessions remained. Two introgressions are found on chromosome 4D from 501 to 502 Mb and chromosome 5D from 555 to 556 Mb, likely from *Ae. tauschii*, verified by both phylogenetic analyses (Fig. 4d, Additional file 2: Figures S30 and S31) and pairwise *dxy* values (Additional file 2: Figures S32 and S33), seem to be specific to landraces and not detected in varieties. This is possibly due to the occasional introgressive hybridization in the wild. These introgressions can not only increase the genetic diversity of wheat populations, but also contribute beneficial alleles related to valuable traits for agriculture. For instance, we identified an introgressive fragment on chromosome 5B (~ 497 to ~ 529 Mb) which harbors 336 genes (Fig. 4c, Additional file 1: Figure S29). The frequency of this alien introgression significantly increased from 0.14 in landraces to 0.59 in varieties. Two previously reported quantitative trait loci (QTLs) associated with disease resistance to leaf spot and stripe rust also map to this region [41, 42] (Fig. 4a), suggesting that the introgressive segment may have been selected for its role in disease resistance in bread wheat.

We investigated co-localization of the introgressive segments and reported QTLs and found a total of 79 introgressive fragments overlapped with 124 QTLs related to multiple important agronomic traits, such as grain yield, disease resistance, and plant height (Fig. 4a, Additional file 10: Table S13). About 9600 genes were identified in these overlapped regions. The non-sense or shift mutations of these genes were examined between introgression and non-introgression accessions. Interestingly, we found a *CAM7* gene (Calmodulin 7, TraesCS2B01G113800), which co-localized with a QTL associated with heading date and kernel weight [43, 44], contained a pre-mature stop mutation (Additional file 2: Figure S34). The stop codon mutation was verified in 18 introgression accessions that we sequenced. *CAM7* has been reported to act as a transcriptional regulator to enhance photomorphogenesis and to regulate gene expression under various light conditions, as well as to regulate root growth and abscisic acid responses [45, 46]. The early termination of this gene might cause its function alteration such as heading date in the introgression accessions. Another case is TraesCS2B01G534200, which is the orthology gene of *ATR2* in *Arabidopsis thaliana* encoding an NADPH-cytochrome P450 reductase. A T-to-A mutation on the third exon in non-introgression accessions made the initial stop codon (TAG) in introgression accessions change to AAG (Lysine) (Additional file 2: Figure S35), which was verified in 11 introgression accessions. This gene co-localized with the QTLs associated with leaf rust and plant height [38, 43]. In plants, *ATR2* is found to be involved in altering tryptophan metabolism [39]. Tryptophan, which is as an important precursor for auxin biosynthesis in plants, plays a vital role in disease resistance and plant growth. The mutation in this gene might be associated with disease resistance to leaf rust and plant height between introgression and non-introgression accessions.

We further performed RNA sequencing to analyze the expression dynamics of the genes located in 45 introgressive regions, where at least two introgression accessions and two non-introgression accessions were sequenced. Eighteen out of 1373 genes expressed differentially (*t* test, corrected *P* < 0.05) between introgression and non-introgression accessions (Additional file 2: Table S14). The expression of TraesCS2B01G113800 and TraesCS2B01G534200 in introgression and non-introgression accessions were also detected and compared. It is no accident that TraesCS2B01G113800 displayed almost no expression in the introgression accessions but highly expressed in the non-introgression accessions (Additional file 2: Figure S34). In addition, TraesCS2B01G534200 showed lower expression in introgression accessions than in non-introgression accessions (Additional file 2: Figure S35).

### Footprints of selection during domestication and improvement

**Fig. 5:**
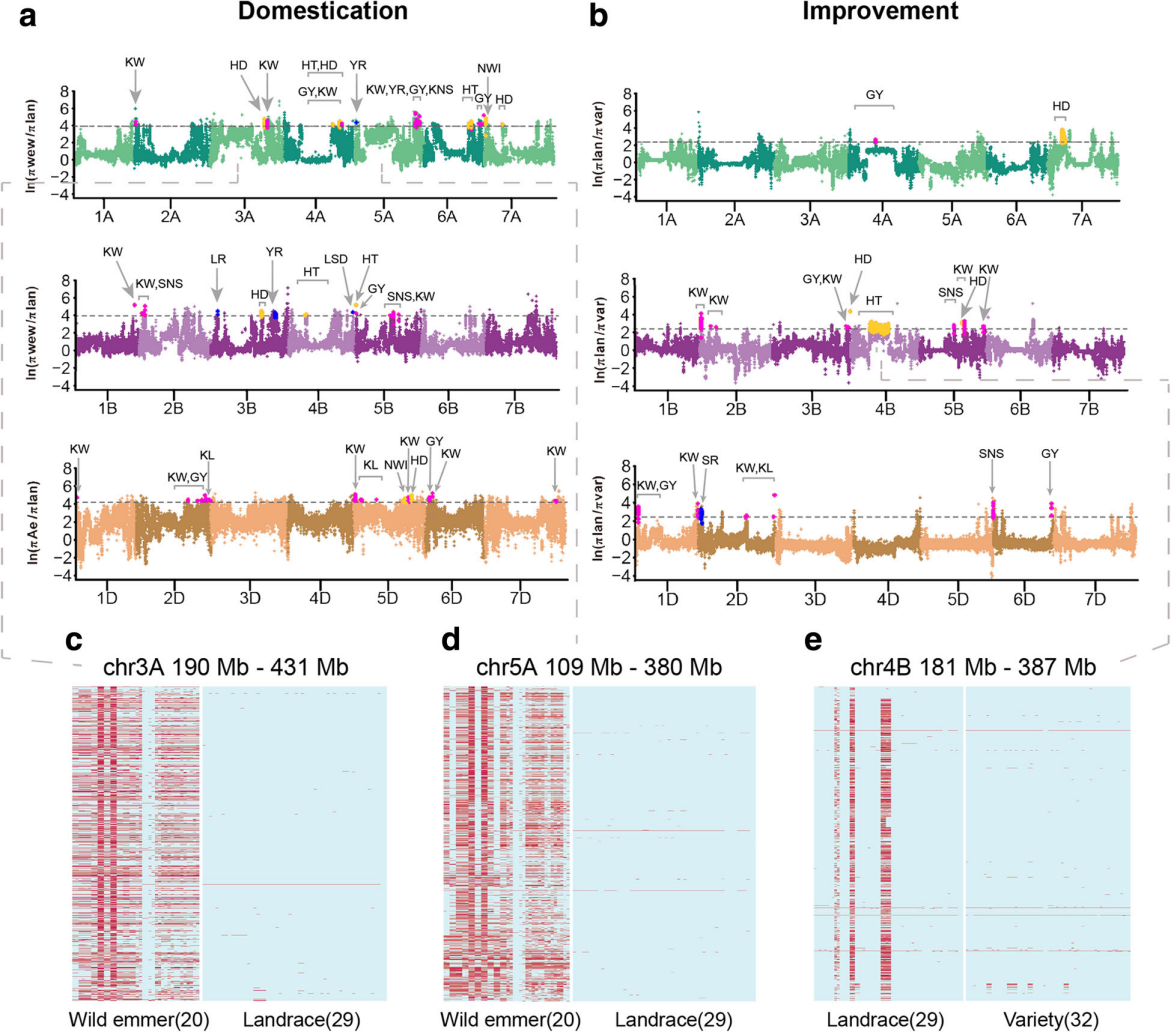
Genome-wide selective signals during domestication and improvement. Whole-genome screening of selective signals during domestication (**a**) and improvement (**b**). The ln *π* ratio values are plotted against the position on each of the 21 chromosomes. The horizontal gray dashed lines show the genome-wide threshold for selective sweeps, ln *π*_wild emmer_/*π*_landrace_ > 3.95, ln *π*_*Ae. auschii*_/*π*_landrace_ > 4.29, and ln *π*_landrace_/*π*_variety_ > 2.41. The previous reported QTLs that overlap with selection signals are highlighted with points in different colors, magenta for QTLs related to yield, blue for QTLs associated with disease resistance, and yellow for development related. All the windows overlapped with QTLs, and the QTLs indicate references and confidence intervals are provided in Additional file 9: Tables S15 and Additional file 10: S16. **c**–**e** The patterns of SNPs of the large regions with long stretches of elevated *π* ratio on chromosomes 3A, 5A, and 4B. Each column is an accession, and each row is an SNP site. Different colors donate the genotypes of SNPs, light blue for reference homozygous sites and red for homozygous non-reference sites

To further study the footprints of selection during the processes of domestication (wild emmer and *Ae*. *tauschii* versus landraces) and improvement (landraces versus varieties), we scanned the whole genome for the regions with elevated differences in genetic diversity (*π* ratio) (see the “Methods” section) to detect candidate selective sweeps (Fig. 5a, b). A total of 547 domestication-related selective sweeps (192, 146, and 209 in A, B, and D subgenome, respectively) (Fig. 5a, Additional file 11: Tables S15, and Additional file 12: S16) and 438 improvement-related selective sweeps (107, 231, and 100 in A, B, and D subgenome, respectively) (Fig. 5b, Additional file 13: Table S17) were identified. The causal mutations of well-known selected loci *TtBtr1-A* and *TtBtr1-B* controlling non-brittle rachis in domesticated emmer [22] were fixed in bread wheat. And the *Q* locus that encodes AP2-like transcription factor controlling free threshing showed a strong selection signal (Fig. 5a). The “circadian rhythm pathway” appears to be an enriched KEGG term (corrected *P* value < 0.05) for genes under selection, which includes seven genes encoding phytochrome or flowering locus T protein (Additional file 2: Table S18). During the improvement process, the GO term of “carbon fixation in photosynthetic organism” pathway is enriched (Additional file 2: Table S19). Notably, the selected regions overlap with 71 reported QTL regions (Fig. 5a, b; Additional file 14: Table S20; Additional file 15: Table S21).

Interestingly, we found several exceptionally large regions with elevated *π* ratio on chromosomes 3A, 5A, 4A, and 4B (Fig. 5c–e). These regions almost always span centromeres with unusually long-range complete LD [40], overlapping with the substantially large haplotype regions that we identified above. It is noted that these regions exhibit a remarkable shift in haplotype frequency during domestication or improvement process. For instance, we found two haploblocks are nearly fixed in all landraces compared with wild emmer, showing greatly reduced diversity on chromosomes 3A and 5A (Fig. 5c, d). On chromosome 4A at ~ 170 to ~ 440 Mb, the frequency of the dominant haplotype in varieties increases from 0.02 (one in wild emmer) to 0.40 in landraces and to 0.97 in varieties, suggesting continuous effects of artificial selection (Additional file 2: Figure S36). Some QTLs associated with grain yield, thousand kernel weight, and heading date were located in this block [41, 43]. Similarly, one haplotype on chromosome 4B (~ 180 to ~ 380 Mb) where a QTL with thousand kernel weight located [42], is fixed in all 34 varieties, which is also found in two wild emmer accessions from Turkey where the haplotype probably originated (Fig. 5e, Additional file 1: Figure S16). Overall, our results suggest that distinct haplotypes may originate from different wild emmer populations, providing additional targets for artificial selection, and the selected haplotypes related to elite performance in agricultural traits, environmental adaption, or disease resistance can rise in frequency through the prolonged processes of domestication and improvement.

In addition, we observed many genomic regions with elevated *F*_st_ displaying remarkable haplotype frequency differences between western and eastern landraces. The highly divergent regions (high *F*_st_) can stretch over tens of Mb across the centromeric regions of chromosomes 1A, 2A, and 5B (Additional file 1: Figure S14). The divergent haplotypes derived from western and eastern landraces are probably due to the population-specific selection for locally adapted traits, but we could not exclude the possible contributions of genetic drift or migration during the spreading of bread wheat.

CNVs may also be the targets of artificial selection, which can result in the differences in allele frequency among populations. We adopted a statistical parameter, relative frequency difference (RFD), to identify these CNVs (see the “Methods” section). The highest 1% RFD value was used as the threshold for identifying selected CNVs (Additional file 2: Figure S37). Selected CNVs can also play a vital role in wheat domestication. For example, the allele frequency of a deletion located at 51 Mb on chromosome 1D decreases from about 0.8 in landraces to 0.2 in varieties. This deletion involved four genes, which significantly enriched for photosynthesis KEGG pathway.

## Discussion

The whole-genome resequencing covering global accessions reported here has provided a novel insight towards the characterization of genetic origin and diversity of wheat. The recently assembled genome reference allowed us to detect small- and large-scale mutations, describe the uneven variation distribution among subgenomes, and reveal the complex genetic population structures. Our data also suggested the valuable genetic resources from the intra- and inter-species introgression in bread wheat. These factors together with artificial selection shaped the genetic architecture of the present bread wheat.

Bread wheat genome consists of three closely related genomes, evolving different genomic properties cross subgenomes. Genomic variation map showed the B subgenome has the most variations, including SNPs, indels, and deleted CNVs. The abundant CNVs identified in diverse accessions of bread wheat indicated the necessity to build a pan-genome. Besides, the largest volumes of deletions and alien introgressions imply the B subgenome may not be the core subgenome. We suspect that those differences have intensified the functional differentiation of subgenomes. But we still need more functional evidences to support this view.

The phylogenetic analysis indicated that all currently grown cultivated wheat, including domesticated emmer, durum, and bread wheat, showed the closest distance to wild emmer from northern Levant. This result supported that the tetraploid emmer wheat and AB subgenome of bread wheat originated from a single domestication event than multiple origins, which remained inconclusive and has been argued among researchers on tetraploid emmer wheat [4, 11-14]. From our results, wild emmer in northern Levant (NL) was the most likely direct progenitor of AB subgenomes of wheat. In spite of lacking the primary domesticated emmer wheat in our collections, we showed that cultivation of bread wheat is a single domestication event with subsequent multiple wild contributions rather than multiple origins. Except for the NL wild emmer, extensive long-range haplotypes were found to belong to wild emmer populations in southern Levant (SL). These haplotypes may be caused by gene flow between wild and domesticated emmer during the spread of domesticated wheat. Though the results that domesticated wheat has a closer genetic relationship with NL wild emmer which could be the consequence of admixture between them, the more admixture components still suggest that northern Levant is the domestication center of tetraploid wheat. Unlike the AB subgenomes contributed by multiple wild populations, it seems like very few *Ae.tauschii* had added into the D subgenome, laying the basis of significant lower genetic diversity of D subgenome than AB. The genetic distance between *Ae. tauschii* and bread wheat D subgenome is much farther than that of wild emmer in AB, implying *Ae. tauschii* is not the most recent progenitor of D subgenome.

European and Asian landraces showed a clear subdivision in bread wheat. With the evidence of geographically and genetically division of wild and domesticated emmer revealed in this research as well as the previous findings [4, 11, 12, 14], we infer this phenomenon was probably related to the differences among source populations of domesticated emmer that landrace-west and landrace-east derived from. One the other hand, there are several differences between the West and the East wheat, including the ripening time, and cooking ways mainly caused by the protein content. Those differences and their underlying genes underwent artificial selection during local breeding and were likely another explanation for the divergence between western and eastern landraces. Unlike landraces, all the varieties derived from a relatively single source. It was likely caused by that the modern varieties developed during Green Revolution were widely adopted by farmers in the 1960s and 1970s [47] and the frequent germplasm exchanges worldwide during globalization.

Previous studies had reported extensive introgression lines of bread wheat, the famous one of which is the 1B1R translocation [36, 37]. Our results present integrated alien introgression profiles across the global bread wheat. The most introgression events are at the low frequency and selective, suggesting local and adaptive occurrences. We expected to detect the introgression signals in varieties since the great efforts of breeders into the crossbreeding, but the extensive introgressed fragments found in landraces, indicated the natural hybridization between bread wheat and wild emmer. However, the introgressed fragments in landraces could also be the consequence of gene flow between landraces and varieties. More work is needed to exposit this phenomenon.

## Conclusion

Whole-genome resequencing of 93 wheat accessions provides a comprehensive whole-genome variation datasets of worldwide bread wheat and its wild progenitors, including diploid *Ae. tauschii* and tetraploid emmer wheat. The genomic diversity of bread wheat which was made up of various resources mainly include self-accumulating mutations due to the decreased constrained selection, multiple genetic contributions of wild emmer, and alien introgressions. The frequency of a series of haplotypes and introgressive fragments dramatically changed in the evolutionary process, suggesting their potential functions and important role in wheat domestication, broadening the genetic basis, and introducing abundant variations to cultivated bread wheat in the breeding process.

This study will increase our understanding of the genetic diversity and evolutionary process of bread wheat. Besides, our data represents a valuable resource for further identifying functional variants contributing to the phenotypic diversity of bread wheat.

## Methods

### Sample collection and whole-genome resequencing

To construct an integrated genetic variation map of bread wheat, we collected whole-genome sequencing data of 93 accessions (Additional file 2: Table S1), including 20 wild emmer accessions from seven countries (Israel, Syria, Turkey, Israel, Bahrain, Jordan, and Lebanon) in the Fertile Crescent; 5 *Aegilops tauschii* accessions from the Caucasus; 5 durum accessions from Canada, Iraq, and Britain; 29 hexploid landraces collected worldwide (Austria, Bulgaria, Andorra, Spain, Italy, Portugal, Jordan, Armenia, Egypt, Afghanistan, Uzbekistan, India, and China); and 34 varieties from the USA, Egypt, India, Australia, and China (Additional file 2: Table S1). Of these, the sequencing data of 75 accessions were generated in this study, and the other 18 accessions are published [20–22].

Young leaves were collected for genomic DNA extraction using a standard cetyltrimethylammonium bromide (CTAB)-based protocol [48]. About 10 μg of genomic DNA was used to construct a paired-end sequencing library following Illumina’s standard pipeline for each accession. The insert size of the sequencing library was approximately 500 bp, and the read length was 150 bp. All libraries were sequenced on an Illumina® HiSeq X Ten platform according to the manufacturer’s standard protocols with an average raw read sequencing coverage ~ 8× for newly sequenced individuals in this study (Additional file 2: Table S1). To further explore the genetic structure in wild emmer and bread wheat, we downloaded the published exon capture data of 33 wild emmer, 31 domesticated emmer, and 26 landrace accessions [6] (Additional file 2: Table S10).

### Variation calling and annotation

Raw reads were trimmed using trimmomatic (version 0.36) [49], and high-quality clean reads were mapped to the Chinese Spring (CS) wheat reference genome (IWGSC RefSeq v1.0) using BWA-MEM (version 0.7.13r1126) with default parameters [50]. In light of the limitation of BWA software regarding chromosome length, we split the chromosome into two parts—the first 400 Mb and the remaining sequence. The marked duplicate reads were removed using Picard tools (version 2.1.1) [51]. Reads with an abnormal insert size (> 10,000, < − 10,000 or = 0) and low mapping quality (< 1) were filtered using Bamtools (version 2.4.1) [52]. Moreover, all reads with multiple hits were removed using ANGSD (version 0.918) [53] to avoid the adverse impact of homoeologous and repetitive sequences on variation identification. Then, SNP/indel detection was performed using the GATK HaplotypeCaller (version 3.5-0 g36282e4) set for diploids with default filtering settings [54]. SNPs were preliminarily filtered using GATK VariantFiltration with the parameter --filterExpression “QD < 2.0 || FS > 60.0 || MQRankSum < − 12.5 || ReadPosRankSum < − 8.0 || SOR > 3.0 || MQ < 40.0.” The filtering settings for indels were “QD < 2.0, FS > 200.0,” and “ReadPosRankSum < − 20.0.” SNPs that did not meet the following criteria were further excluded: (1) a total read depth (DP) > 240 and < 2200; (2) minor allele frequency (MAF) ≥ 0.05 for each population, and for *Ae. tauschii* (*n* = 5), MAF should be ≥ 0.2; (3) a maximum missing rate < 0.1; and (4) biallelic alleles. Only variations detected by both GATK and ANGSD [53] were used for further analysis. Finally, a total of 84,594,991 SNPs and 11,628,085 indels were obtained. The distributions of SNPs and indels in wild emmer, *Ae. tauschii*, landraces, and varieties are shown in Additional file 1: Figure S1 and Additional file 2: Table S2. The accuracy of these SNPs was estimated by using the wheat 660 K SNP arrays by scanning four resequencing samples selected from different populations (Additional file 2: Table S3), providing the confidence and the accuracy of the SNP calling process. The SNPs of each accession were counted (Additional file 2: Table S1). CS only has 340 heterozygous SNPs, further proving the accuracy of the SNP calling process.

SNP and indel annotations were performed according to the wheat genome annotation using the software SnpEff (version 4.3p) [55]. Based on the genome annotation, SNPs/indels were categorized into exonic regions, splicing sites, 5′UTRs, 3′UTRs, intronic regions, upstream and downstream regions, and intergenic regions (Additional file 2: Table S2). SNPs in the coding exons were further classified into synonymous or nonsynonymous SNPs.

### Identification of CNVs

Copy number variations (CNVs) (deletion and duplication) were called using CNVcaller [56]. The aligned read number was counted along the whole genome using 1000-bp sliding windows with a 500-bp step size. Candidate deleted and duplicated windows were initially defined as 1000-bp windows with a read depth < 0.25 and > 1.75, respectively. CNVs were initially defined as having five of seven or more consecutive 1000-bp overlapping windows with the read depth values described above. The initial calls of CNVs with the distance of less than 20% of their combined length were merged. The processes above were performed for each of the 93 accessions separately. To study the CNVs among accessions, we intersected calls from the whole genome excluding the introgression regions and defined two calls as overlapping calls if they have > 50% reciprocal overlap. Then, the overlapping calls were reduced for redundancy by selecting the largest call of all overlapped ones.

To detect sequence deletions in IWGSC RefSeq v1.0, high-quality clean reads were additionally mapped to a pseudo-genome combining the wild emmer wheat reference genome [22] and *Ae. tauschii* reference genome [20] to provide a more comprehensive CNVs dataset. The method of CNV calling for the mapping results against the pseudo-genome was the same as described above. Overlapped CNVs with fine synteny between the IWGSC RefSeq v1.0 and pseudo-genome were removed from this dataset.

### Population genetics analysis

Due to the highly repetitive nature of the wheat genome, especially in the intergenic regions, only the SNPs located in the genic regions were used to construct NJ trees for the A, B, and D subgenomes with PHYLIP (version 3.68) [57]. Interactive Tree of Life (iTOL) was used to visualize these trees [58]. To further explore the population structure within wild emmer, we combined our data with a set of SNPs of wild and domesticated emmer wheat generated by exome capture sequencing [22]. PCA was performed using the whole-genome SNPs with the smartpca program embedded in EIGENSOFT (version 4.2) [59]. ADMIXTURE (version 1.3.0) software was used to quantify the genome-wide population structures [60]. ADMIXTURE was run for *k* values from 2 to 7 with 20 bootstrapping replicates to estimate the standard errors of parameters. The divergence between landrace-west and landrace-east (Additional file 2: Table S11) was further confirmed by an extended dataset containing the exome capture data of 26 worldwide landraces [6] (Additional file 1: Figure S13).

### Haploblocks analysis and validation

Haplotype blocks were identified using PLINK [61] with the parameters (--blocks no-pheno-req --blocks-max-kb 1000 --geno 0.1 --blocks-min-maf 0.1) in the landrace population. SNPs with a frequency lower than 0.1 and a missing rate higher than 0.1 were discarded in the process of block identification. The parameter --blocks-max-kb may limit the extent of haplotype blocks because the blocks with a length of 1 Mb appear to be continuous in certain regions, except for few small intervals (usually < 10 kb) between two adjacent 1-Mb blocks. The same phenomenon emerged even when we increased the bound with parameter --blocks-max-kb to 2 Mb or longer. Therefore, we merged these 1 Mb haploblocks into a long-range haploblock using a custom perl script. Each of the haploblock was validated by examining the patterns of haplotype sharing using SNPs and by constructing phylogenetic trees.

### Estimation of the divergence time among haplotypes

Two models, isolation (I) and isolation-with-migration (IM), were used to estimate the divergence time among the three major haplotypes on chromosome 4A (haplotype-a, haplotype-b, and haplotype-c) using IM-CoalHMM [29] (Additional file 1: Figure S18). All bam files of each haplotype were combined and used as the input of ANGSD to call the consensus sequences with the parameters -doFasta 2 -doCounts 1 -minQ 20 -minMapQ 20 -only_proper_pairs 1 -uniqueOnly 1 -remove_bads 1. To evaluate the variation in estimates, the consensus haplotype sequences were divided into 10 Mb with 5 Mb overlapping segments. We then analyzed the haplotype pairs in segments using both the I model and IM model. The estimated model parameters were rescaled to years using a per-generation mutation rate of 0.69 × 10^−8^ and a generation time of 1 year.

### Identification of genome-wide introgressions

As indicated by 1B1R translocation [36, 37], introgression possessed two obvious characteristics: (1) except for genic and repetitive regions, few reads were mapped to the introgressed regions, resulting in identification as deleted CNVs; (2) though a few mapped to the introgressed region, the reads have excessive divergent sites compared with the reference genome. SNP density has been used to identify introgression in wheat, and two of the identified introgressed segments have been traced to their origins [35]. For each deleted CNV, we calculate the density of divergent sites in a 50-kb sliding window using ANGSD [53] for each accession, except for CS (the IWGSC RefSeq v1.0). The command is as follows: angsd -i bam -only_proper_pairs 1 -uniqueOnly 1 -r window -remove_bads -minQ 20 -anc refence_genome -ref reference_genome -dosaf 1 -out $out -skipTriallelic 1 -setMinDepth 1 -setMaxDepth 20 -P 6 -doMajorMinor 1 -doCounts 1 -GL 1.

Both heterozygous and homologous sites different from the IWGSC RefSeq v1.0 were defined as the divergent sites. Then, the ratio of divergent sites (the number of divergent sites /the sequence length covered by reads) was calculated. *Z* tests were performed on the ratios of divergent sites for all of the non-overlapped 50-kb sliding windows for each individual to identify high outliers with *P* < 0.05 (*Z* score > 1.644853627) in the landraces and varieties, respectively. The windows identified as outliers for each individual were defined as candidate introgressed windows. For each accession, we then counted the numbers of candidate introgressed window for all the deleted CNVs. If more than half of the windows for a deleted CNV belong to the candidate introgressed windows, this deleted CNV was ultimately determined as a putative introgressed region. The deleted CNV covered the 1B1R translocation in C42, and C46 contains 4982 windows, of which 3143 (63%) passed the *Z* test and were identified as candidate introgressed windows, suggesting the validation of this method. To decrease the false-positive rate, only the deleted CNVs with a length ≥ 500 kb (≥ 10 windows) were used for the analysis.

To identify the original donors of these introgressed segments, we selected several species, including barley (*Hordeum vulgare*), rye (*Secale cereale*), *T. urautu*, wild emmer, and *Ae. tauschii*, as candidates. The reads generated by whole-genome resequencing or short sequences obtained by breaking the reference genomes of the species mentioned above were mapped to the IWGSC RefSeq v1.0. Consensus sequences were obtained with ANGSD for the 93 whole-genome resequencing accessions and the candidate original donors. The command was as follows: angsd -i bam -rf region -only_proper_pairs 0 -uniqueOnly 1 -remove_bads 1 -nThreads 5 -minQ 20 -minMapQ 1 -doFasta 1 -basesPerLine 100 -doCounts 1 -out output. Then, we constructed phylogenetic trees and calculated the absolute divergence (*dxy*) among accessions using the consensus sequences to verify these introgression segments.

### Gene expression analysis of introgressive fragments

The samples were collected from the roots and leaves from 3 wild emmer, 3 *Ae. tauschii*, 3 hexaploid landraces, and 3 hexaploid varieties, which also had been re-sequenced. Total RNA was extracted using the Qiagen Plant Tissue kit. In total, 24 paired-end libraries were prepared and sequenced on an Illumina® HiSeq X Ten platform with 150-bp read length. The raw RNA-seq reads were filtered for contamination with adaptor reads, low-quality reads, or unknown nucleotides using FastQC (version 0.11.7) [62] and Trimmomatic (version 0.36) [49]. Filtered reads from each sample were mapped to the IWGSC RefSeq (version 1.0) with HISAT2 (version 2.1.0) [63]. Then, the alignments were used for transcript assembly with StringTie v1.3.3b [64]. After the transcript assembly of each sample, the results were merged using StringTie’s merge function.

The introgression regions were used to perform the analysis of differentially expressed genes (DEGs). The genes with the FPKM in all samples lower than 1 or even not expressed were discarded. The gene expression difference between introgression and non-introgression accessions were examined using *t* test. And then, the *P* value was corrected using p.adjust function in R.

### Genome scanning for selective signals

For selective signal identification, we divided the process into two periods: (1) domestication from wild progenitors to landraces and (2) improvement from landraces to varieties. The population statistics used as indicators of selection included nucleotide diversity and *F*_st_. The genetic diversity in the wild progenitors (wild emmer and *Ae. tauschii*) was compared with that in the landrace (*π*_wild emmer_/*π*_landrace_, *π*_*Ae. tauschii*_/*π*_landrace_), and the genetic diversity in the landrace was compared with that in the variety group (*π*_landrace_/*π*_variety_). Candidate selective windows (non-overlapping 100-kb windows) were identified with the top 1% of values. Selective windows that were separated by a distance of < 200 kb were merged as selective sweeps. The protein sequences of the genes located in selective regions were used to perform KEGG analysis on KOBAS [65].

*F*_st_ values were also calculated in non-overlapping 100-kb windows. To run *F*_st_, we reduced the number of false positives by excluding the windows with fewer than 20 SNPs because the variance of *F*_st_ depends on the number of SNPs used for calculation.

### Physical position of QTLs

We collected the previously reported QTLs and GWAS signals associated with various traits, including grain yield (GY), kernel number per spike (KNS), kernel weight (KW), kernel length (KL), spikelet number per spike (SNS), leaf spot disease (LSD), reaction to leaf rust (LR), reaction to *Puccinia graminis* Pers (SR), reaction to *Puccinia striiformis* Westend (YR), plant height (HT), heading date (HT), sprouting (SP), normalized water index (NWI), and grain color.

We determined the boundaries of QTLs and GWAS signals by blasting their sequences to the reference. For the signals without sequences provided, we extended 2 Mb upstream and downstream as their boundaries. Then, we used these boundaries to examine whether the QTLs or GWAS signals overlap with introgressions or selective signals.

## Additional files

Additional file 1: Figure S1. Venn diagram represents the population-specific and overlapped SNPs among different populations. **Figure S2.** The frequency spectrum of SNPs in the A, B, and D subgenomes.** Figure S3.** The relative diversity of A and B subgenomes for wild emmer, durum, landrace and variety wheat. **Figure S4.** Nucleotide diversity (π) of the A subgenome of wild emmer, durum, the landraces, and the varieties. **Figure S5.** Nucleotide diversity (π) of the B subgenome of wild emmer, durum, the landraces, and the varieties. **Figure S6.** Nucleotide diversity (π) of the D subgenome of Ae. tauschii, the landraces, and the varieties. **Figure S7. **Neighbour-joining (NJ) trees of 93 accessions on the A, B and D subgenomes. **Figure S8.** Neighbour-joining (NJ) trees of 152 accessions on the A and B subgenomes. **Figure S9.** Principal component analysis of all accessions in the A, B, and D subgenomes. **Figure S10.** Population structure of AB and D subgenomes using ADMIXTURE. **Figure S11.** Absolute sequence divergence dxy value (the number of pairwise differences per site) between each pairwise accessions on A and B subgenomes. **Figure S12.** Principal component analysis (PCA) plots based on the first two principal components of 63 bread wheat accessions on A, B, and D subgenomes. **Figure S13.** ADMIXTURE analysis of 93 wheat accessions combined with the exon capture data of 26 landraces. **Figure S14.** Population divergence(*F*_st_) of A, B and D subgenome. **Figure S15.** Nucleotide diversity (π) and population divergence (*F*_st_) across five or four groups. **Figure S16.** Haplotype patterns and phylogenetic analysis of regions which length larger than 10Mb. **Figure S17.** Neighbour-joining phylogenetic tree of the longest haploblock on chromosome 4A. **Figure S18.** Divergence time of the different haplotypes of the longest haploblocks on chromosome 4A. **Figure S19.** Haplotype patterns of chromosome 4A in diverse populations. **Figure S20.** Boxplots of the sequence identity between reads of C46 mapped to chromosome 1BS and the sequence of outgroups. (DOCX 18309 kb)

Additional file 2: Figure S21. The maximum likelihood trees of the 1B/1R translocation region on chromosome 1B from 1 to 240 Mb. **Figure S22.** Absolute sequence divergence dxy value (the number of pairwise differences per site) between each pairwise accession. **Figure S23.** The pattern of haplotypes sharing in diverse populations on chromosome 1B. **Figure S24.** The mapping statistics of the resequencing data in introgressed regions visualized by Integrative Genomics Viewer (IGV). **Figure S25.** The length of the introgressions of each bread wheat accession.** Figure S26.** The length of the introgressions of A, B, and D subgenomes. **Figure S27.** The introgressied segments with accurate breakpoints identified in Chinese Spring. **Figure S28.** Single nucleotide polymorphism (SNP) density across chromosome 2D of the Chinese Spring. **Figure S29.** The maximum likelihood tree of a 33 Mb introgression fragment on chromosome 5B from ~ 497 Mb to 529 Mb (853000 SNP). **Figure S30.** The maximum likelihood tree of an introgression fragment from *Ae.tauschii*. **Figure S31.** The maximum likelihood tree of a introgression segment on chromosome 5D form 555, 981, 000 bp to 556, 438, 500 bp (70000 SNP). **Figure S32.** Absolute sequence divergence dxy value (the number of pairwise differences per site) between each pairwise accessions on chromosome 4D from 500, 862, 001 bp to 501, 999, 000 bp. **Figure S33.** Absolute sequence divergence dxy value (the number of pairwise differences per site) between each pairwise accessions on chromosome 5D form 555, 981, 000 bp to 556, 438, 500 bp. **Figure S34. **Comparison of mapping statistics and gene expression of TraesCS2B01G113800 in intro (introgression) and non-intro (non-introgression) accessions. **Figure S35.** Comparison of mapping statistics and gene expression of TraesCS2B01G534200 in intro (introgression) and non-intro (non-introgression) accessions. **Figure S36.** The frequency of haplotype-c. **Figure S37.** Relative frequency difference (RFD) of CNVs between landraces and varieties. **Table S1.** Detailed information of the 93 accessions used in this study. **Table S2. **Statistics of the whole-genome SNPs and indels. **Table S3.** The accuracy of the SNPs calling determined by wheat 660K array. **Table S10.** Detailed information of the exon capture data of wheat used in this study. **Table S11.** Two groups of the landraces, Landrace-west and Landrace-east. **Table S14.** The list of differentially expressed genes (DEGs) between intro and non-intro accessions. **Table S18. **Summary of the KEGG enrichment analysis of genes located in selective regions during domestication process. **Table S19.** Summary of the KEGG enrichment analysis of genes located in selective regions during the improvement process. (DOCX 3387 kb)

Additional file 3: **Table S4.** The deleted CNV regions (CNVRs) on A subgenome of wheat based on IWGSC Refseq v1.0. (XLSX 15732 kb)

Additional file 4: **Table S5.** The deleted CNV regions (CNVRs) on B subgenome of wheat based on IWGSC Refseq v1.0. (XLSX 17806 kb)

Additional file 5: **Table S6.** The deleted CNV regions (CNVRs) on D subgenome of wheat based on IWGSC Refseq v1.0. (XLSX 6430 kb)

Additional file 6: **Table S7.** The duplicated CNV regions (CNVRs) on A subgenome of wheat based on IWGSC Refseq v1.0. (XLSX 13571 kb)

Additional file 7: **Table S8.** The duplicated CNV regions (CNVRs) on B subgenome of wheat based on IWGSC Refseq v1.0. (XLSX 18439 kb)

Additional file 8: **Table S9.** The duplicated CNV regions (CNVRs) on D subgenome of wheat based on IWGSC Refseq v1.0. (XLSX 7229 kb)

Additional file 9: **Table S12.** List of haplotype blocks with a length > 1 Mb along the A, B, and D subgenomes. (XLSX 39 kb)

Additional file 10: **Table S13.** The introgressions overlapped with the previously mapped QTLs. (XLSX 23 kb)

Additional file 11: **Table S15.** Genomic regions identified as a selective sweep by nucleotide diversity (*π*) between wild emmer and the landraces. (XLSX 40 kb)

Additional file 12: **Table S16.** Genomic regions identified as a selective sweep by nucleotide diversity (*π*) between *Ae. tauschii* and the landraces. (XLSX 27 kb)

Additional file 13: **Table S17.** Genomic regions identified as a selective sweep by nucleotide diversity (*π*) between the landraces and varieties. (XLSX 36 kb)

Additional file 14: **Table S20.** Overlapping regions between QTLs and putative selective sweeps at domestication period. (XLSX 21 kb)

Additional file 15: **Table S21.** Overlapping regions between QTLs and putative selective sweeps at improvement period. (XLSX 22 kb)

Additional file 16: Review history. (DOCX 478 kb)

## Data Availability

The raw reads including whole-genome resequencing and RNA sequencing in this study are publicly available at the NCBI Sequence Read Archive under accession code PRJNA476679 [66]. The sequence reads of Chinese Spring (CS) and an *Ae. tauschii* accession (A1) analyzed during the study were reported previously [19–21]. The NCBI accessions are PRJNA329335 (SRR5170323, SRR5184282, and SRR5184283) and PRJNA392179 (SRR5815659, SRR5817288, SRR5817289, and SRR5817290), respectively. The sequence reads of 16 download variety accessions are available from the website https://downloads-qcif.bioplatforms.com/bpa/wheat_cultivars/cultivars/. The exome capture data of landrace accessions were downloaded from NCBI Short Read Archive (SRP032974). The exome capture SNP dataset in the VCF format for wild and domesticated emmer is available from the website https://www.dropbox.com/sh/3dm05grokhl0nbv/AABe6yrr2FVXdFasJUYEW12ca/Allelic%20diversity?dl=0&preview=all_emmer_filtered_variants_header_to_SAMN04448013.vcf.

## References

[1] Jared D (2002). Evolution, consequences and future of plant and animal domestication. Nature.

[2] Kilian B (2006). Independent wheat B and G genome origins in outcrossing Aegilops progenitor haplotypes. Mol Biol Evol.

[3] Tanno K-I, Willcox G (2006). How fast was wild wheat domesticated?. Science.

[4] Luo MC (2007). The structure of wild and domesticated emmer wheat populations, gene flow between them, and the site of emmer domestication. Theor Appl Genet.

[5] Cavanagh CR (2013). Genome-wide comparative diversity uncovers multiple targets of selection for improvement in hexaploid wheat landraces and cultivars. Proc Natl Acad Sci U S A.

[6] Jordan KW (2015). A haplotype map of allohexaploid wheat reveals distinct patterns of selection on homoeologous genomes. Genome Biol.

[7] Wang S (2014). Characterization of polyploid wheat genomic diversity using a high-density 90 000 single nucleotide polymorphism array. Plant Biotechnol J.

[8] Akhunov ED (2010). Nucleotide diversity maps reveal variation in diversity among wheat genomes and chromosomes. BMC Genomics.

[9] Wicker T (2018). Impact of transposable elements on genome structure and evolution in bread wheat. Genome Biol.

[10] Thomas M (2014). Ancient hybridizations among the ancestral genomes of bread wheat. Science.

[11] Feldman M, Kislev ME (2007). Domestication of emmer wheat and evolution of free-threshing tetraploid wheat. Israel J Plant Sci.

[12] Özkan H (2010). Geographic distribution and domestication of wild emmer wheat (Triticum dicoccoides). Genet Resour Crop Evol.

[13] Ozkan H (2005). A reconsideration of the domestication geography of tetraploid wheats. Theor Appl Genet.

[14] Mori N, Ishi T, Ishido T, et al. Origins of domesticated emmer and common wheat inferred from chloroplast DNA fingerprinting. In: Pogna NE, Romano M, Pogna EA, Galterio G(eds) Proceedings of the 10th International Wheat Genetics Symposium, Paestum, Italy. Rome: Istituto Sperimentale per la Cerealicoltura; 2003. pp 25–28.

[15] Dvorak J (1998). The structure of the Aegilops tauschii genepool and the evolution of hexaploid wheat. Theor Appl Genet.

[16] Jirui W (2013). Aegilops tauschii single nucleotide polymorphisms shed light on the origins of wheat D-genome genetic diversity and pinpoint the geographic origin of hexaploid wheat. New Phytol.

[17] Hedden P (2003). The genes of the Green Revolution. Trends Genet.

[18] Molnár-Láng M, Ceoloni C, Doležel J. Alien introgression in wheat. Springer International Publishing Switzerland; 2015.

[19] Appels R (2018). Shifting the limits in wheat research and breeding using a fully annotated reference genome. Science.

[20] Jia J (2013). Aegilops tauschii draft genome sequence reveals a gene repertoire for wheat adaptation. Nature.

[21] Montenegro JD (2017). The pangenome of hexaploid bread wheat. Plant J Cell Mol Biol.

[22] Avni R (2017). Wild emmer genome architecture and diversity elucidate wheat evolution and domestication. Science.

[23] Beissinger TM (2016). Recent demography drives changes in linked selection across the maize genome. Nature plants.

[24] Zimin AV, et al. The first near-complete assembly of the hexaploid bread wheat genome, Triticum aestivum. Gigascience. 2017;6(11):1–7. 10.1093/gigascience/gix097 PMC5691383 29069494

[25] Ormoli L (2015). Diversity trends in bread wheat in Italy during the 20th century assessed by traditional and multivariate approaches. Sci Rep.

[26] Ai H (2015). Adaptation and possible ancient interspecies introgression in pigs identified by whole-genome sequencing. Nat Genet.

[27] Ozkan H (2002). AFLP analysis of a collection of tetraploid wheats indicates the origin of emmer and hard wheat domestication in southeast Turkey. Mol Biol Evol.

[28] Kronstad WE. Agricultural development and wheat breeding in the 20th century, in wheat: prospects for global improvement, pp. 1–10. Dordrecht/Boston/London: Kluwer Academic Publishers.

[29] Dutheil JY (2009). Ancestral population genomics: the coalescent hidden Markov model approach. Genetics.

[30] Shaoxing H (2002). Genes encoding plastid acetyl-CoA carboxylase and 3-phosphoglycerate kinase of the Triticum/Aegilops complex and the evolutionary history of polyploid wheat. Proc Natl Acad Sci U S A.

[31] Dvorak J, Akhunov ED (2005). Tempos of gene locus deletions and duplications and their relationship to recombination rate during diploid and polyploid evolution in the Aegilops-Triticum alliance. Genetics.

[32] Rong JK (2000). A new powdery mildew resistance gene: introgression from wild emmer into common wheat and RFLP-based mapping. Euphytica.

[33] Hegde SG, Waines JG (2004). Hybridization and introgression between bread wheat and wild and weedy relatives in North America. Crop Sci.

[34] Niu Z (2011). Targeted introgression of a wheat stem rust resistance gene by DNA marker-assisted chromosome engineering. Genetics.

[35] Thind AK (2018). Chromosome-scale comparative sequence analysis unravels molecular mechanisms of genome dynamics between two wheat cultivars. Genome Biol.

[36] Mccracken KJ (2008). Lack of relationship between either specific weight or presence of the 1B1R gene and nutritive value of wheat in broiler diets. Br Poult Sci.

[37] Villareal R (1995). The 1BL/1RS chromosome translocation effect on yield characteristics in a Triticum aestivum L. cross. Plant Breed.

[38] Aoun, M., et al., Genome-wide association mapping of leaf rust response in a durum wheat worldwide germplasm collection*.* Plant Genome, 2016. 9(3). 10.3835/plantgenome2016.01.0008 27902791

[39] Smolen GA (2002). Dominant alleles of the basic helix-loop-helix transcription factor ATR2 activate stress-responsive genes in Arabidopsis. Genetics.

[40] Cui F (2017). Utilization of a Wheat660K SNP array-derived high-density genetic map for high-resolution mapping of a major QTL for kernel number. Sci Rep.

[41] Peleg Z (2011). Genetic analysis of wheat domestication and evolution under domestication. J Exp Bot.

[42] Guan P, et al. Global QTL Analysis Identifies Genomic Regions on Chromosomes 4A and 4B Harboring Stable Loci for Yield-Related Traits Across Different Environments in Wheat (Triticum aestivum L.). Front Plant Sci. 9:529. 10.3389/fpls.2018.00529. 10.3389/fpls.2018.00529 PMC5996883 29922302

[43] Zhang J, et al. Identification and validation of QTL for grain yield and plant water status under contrasting water treatments in fall-sown spring wheats. Theor Appl Genet. 2018;131(8):1741–59. 10.1007/s00122-018-3111-9 PMC6061171 29767279

[44] Ramya P (2010). QTL mapping of 1000-kernel weight, kernel length, and kernel width in bread wheat (Triticum aestivum L.). J Appl Genet.

[45] Abbas N (2014). Arabidopsis CAM7 and HY5 physically interact and directly bind to the HY5 promoter to regulate its expression and thereby promote photomorphogenesis. Plant Cell.

[46] Abbas N, Chattopadhyay S (2014). CAM7 and HY5 genetically interact to regulate root growth and abscisic acid responses. Plant Signal Behav.

[47] Evenson RE, Gollin D (2003). Assessing the impact of the Green Revolution, 1960 to 2000. Science.

[48] Murray MG, Thompson WF (1980). Rapid isolation of high molecular weight plant DNA. Nucleic Acids Res.

[49] Bolger AM, Lohse M, Usadel B (2014). Trimmomatic: a flexible trimmer for Illumina sequence data. Bioinformatics.

[50] Li H, Durbin R (2009). Fast and accurate short read alignment with Burrows-Wheeler transform. Bioinformatics.

[51] Wysokar, A., et al., Picard: a set of tools for working with next generation sequencing data in BAM format*.* Retrieved Aug 2014 from http://broadinstitute.github.io/picard, 2014.

[52] Barnett DW (2011). BamTools: a C++ API and toolkit for analyzing and managing BAM files. Bioinformatics.

[53] Sand KT, Anders A, Rasmus N (2014). ANGSD: analysis of next generation sequencing data. BMC Bioinformatics.

[54] McKenna A, et al. The Genome Analysis Toolkit: a MapReduce framework for analyzing next-generation DNA sequencing data. Genome Res. 2010;20(9):1297–303. 10.1101/gr.107524.110 PMC2928508 20644199

[55] Cingolani P, et al. A program for annotating and predicting the effects of single nucleotide polymorphisms, SnpEff: SNPs in the genome of Drosophila melanogaster strain w1118; iso-2; iso-3. Fly. 2012;6(2):80–92. 10.4161/fly.19695 PMC3679285 22728672

[56] Wang X (2017). CNVcaller: highly efficient and widely applicable software for detecting copy number variations in large populations. Gigascience.

[57] Felsenstein, J. PHYLIP: Phylogeny Inference Package, University of Washington, Seattle, WA. 1993.

[58] Letunic I, Bork P (2006). Interactive Tree Of Life (iTOL): an online tool for phylogenetic tree display and annotation. Bioinformatics.

[59] Price AL (2006). Principal components analysis corrects for stratification in genome-wide association studies. Nat Genet.

[60] Alexander DH, Novembre J, Lange K. Fast model-based estimation of ancestry in unrelated individuals. Genome Res. 2009;19(9):1655–64. 10.1101/gr.094052.109 PMC2752134 19648217

[61] Purcell S (2007). PLINK: a tool set for whole-genome association and population-based linkage analyses. Am J Hum Genet.

[62] Andrews S (2010). FastQC: a quality control tool for high throughput sequence data.

[63] Kim D, Langmead B, Salzberg S (2017). HISAT2: graph-based alignment of next-generation sequencing reads to a population of genomes.

[64] Pertea M (2015). StringTie enables improved reconstruction of a transcriptome from RNA-seq reads. Nat Biotechnol.

[65] Xie C (2011). KOBAS 2.0: a web server for annotation and identification of enriched pathways and diseases. Nucleic Acids Res.

[66] Cheng H, Liu J, Wen J, Xu L, Chen N , Li Z, Nie X, Wang Q , Zheng Z , Li M, et al. Frequent intra- and inter-species introgression shape the landscape of genetic variation in bread wheat Data sets. NCBI. https://www.ncbi.nlm.nih.gov/bioproject/PRJNA476679 (2018). 10.1186/s13059-019-1744-x PMC6624984 31300020

