# Supplementary material for: Frequent intra- and inter-species introgression shapes the landscape of genetic variation in bread wheat
Source: Genome Biol. 2019 Jul 12;20:136. doi: 10.1186/s13059-019-1744-x (PMC6624984; doi:10.1186/s13059-019-1744-x)
Supplement: Supplementary file 1 — Venn diagram represents the population-specific and overlapped SNPs among different populations. Figure S2. The frequency spectrum of SNPs in the A, B, and D subgenomes. Figure S3. The relative diversity of A and B subgenomes for wild emmer, durum, landrace and variety wheat. Figure S4. Nucleotide diversity (π) of the A subgenome of wild emmer, durum, the landraces, and the varieties. Figure S5. Nucleotide diversity (π) of the B subgenome of wild emmer, durum, the landraces, and the varieties. Figure S6. Nucleotide diversity (π) of the D subgenome of Ae. tauschii, the landraces, and the varieties. Figure S7. Neighbour-joining (NJ) trees of 93 accessions on the A, B and D subgenomes. Figure S8. Neighbour-joining (NJ) trees of 152 accessions on the A and B subgenomes. Figure S9. Principal component analysis of all accessions in the A, B, and D subgenomes. Figure S10. Population structure of AB and D subgenomes using ADMIXTURE. Figure S11. Absolute sequence divergence dxy value (the number of pairwise differences per site) between each pairwise accessions on A and B subgenomes. Figure S12. Principal component analysis (PCA) plots based on the first two principal components of 63 bread wheat accessions on A, B, and D subgenomes. Figure S13. ADMIXTURE analysis of 93 wheat accessions combined with the exon capture data of 26 landraces. Figure S14. Population divergence(Fst) of A, B and D subgenome. Figure S15. Nucleotide diversity (π) and population divergence (Fst) across five or four groups. Figure S16. Haplotype patterns and phylogenetic analysis of regions which length larger than 10Mb. Figure S17. Neighbour-joining phylogenetic tree of the longest haploblock on chromosome 4A. Figure S18. Divergence time of the different haplotypes of the longest haploblocks on chromosome 4A. Figure S19. Haplotype patterns of chromosome 4A in diverse populations. Figure S20. Boxplots of the sequence identity between reads of C46 mapped to chromosome 1BS and the sequence of outgroups. [file 13059_2019_1744_MOESM1_ESM.docx]

Supplementary Materials for

**Frequent intra- and inter-species introgression shape the landscape of genetic variation in bread wheat**

This file includes:

Figures S1 to S20

**
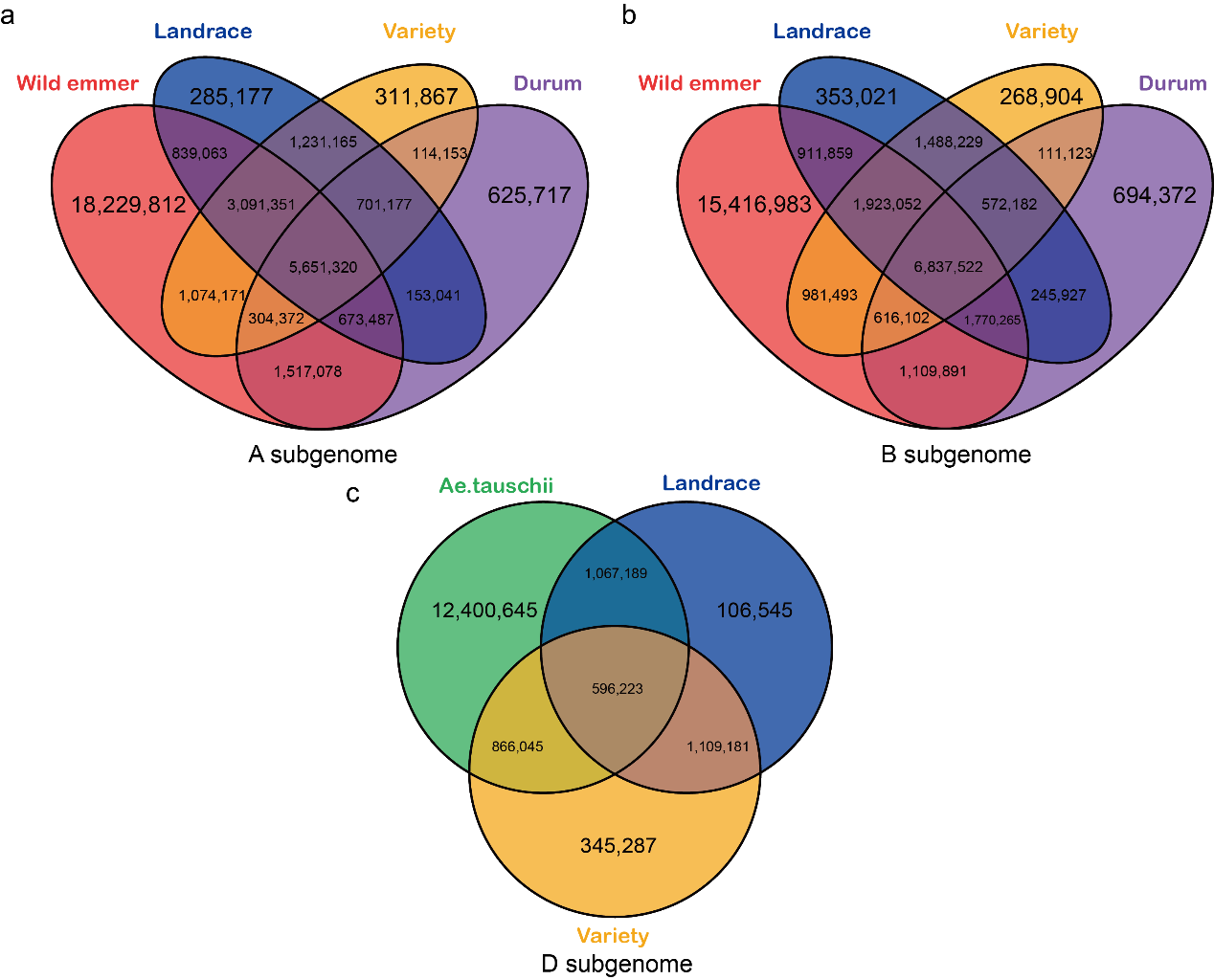
**

**Figure S1: Venn diagram represents the population-specific and overlapped SNPs among different populations.** A total of 18,229,812 and 15,416,983 SNPs were specific to wild emmer in A and B subgenomes respectively, and 12,400,645 SNPs specific to *Ae. tauschii* in D subgenome.


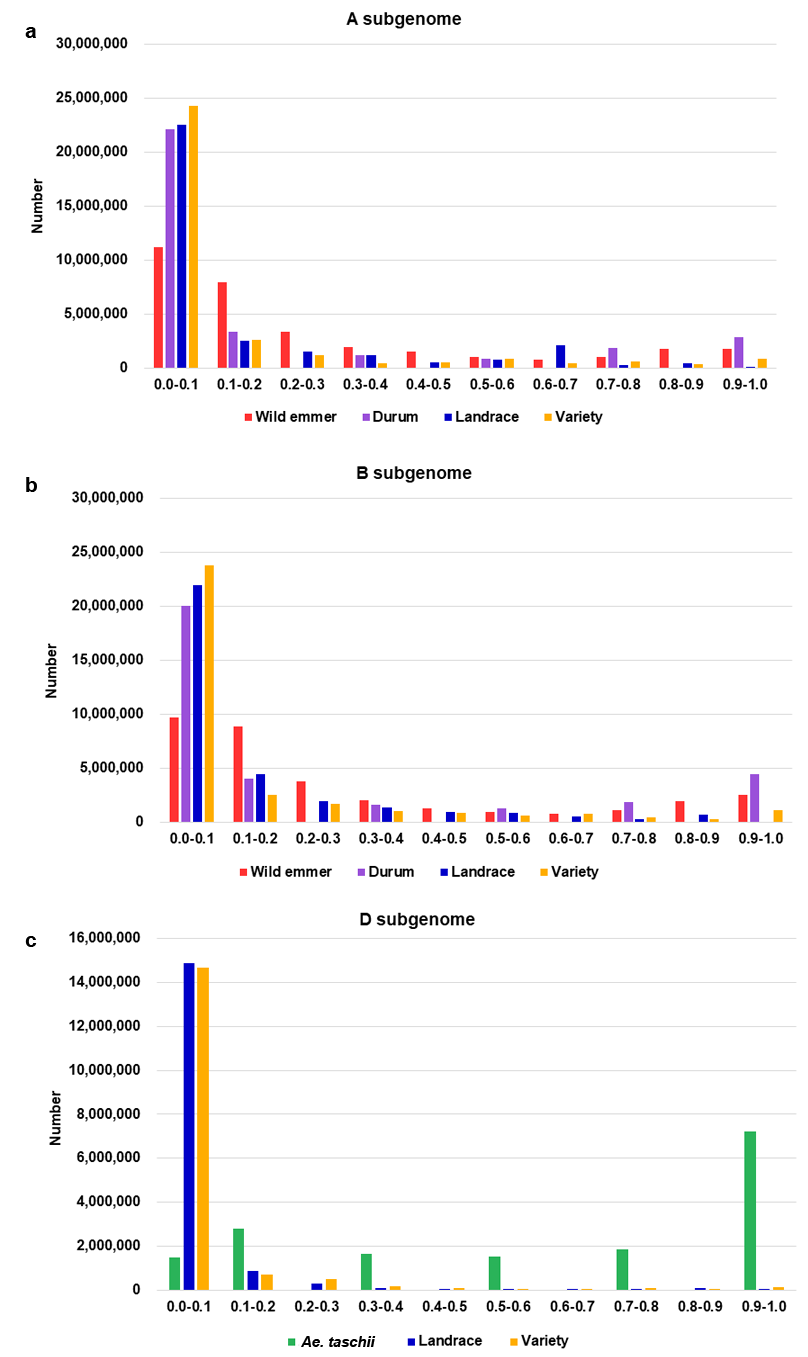


**Figure S2: The frequency spectrum of SNPs in the A, B, and D subgenomes.** The number of SNPs with different frequencies is shown, with different colours indicating different populations. The proportion of SNPs with a low frequency (≤ 0.1) accounts for more than 80% of the SNPs in the D subgenome in the landraces and varieties, which is approximately 10-percent higher than those in the A and B subgenomes.


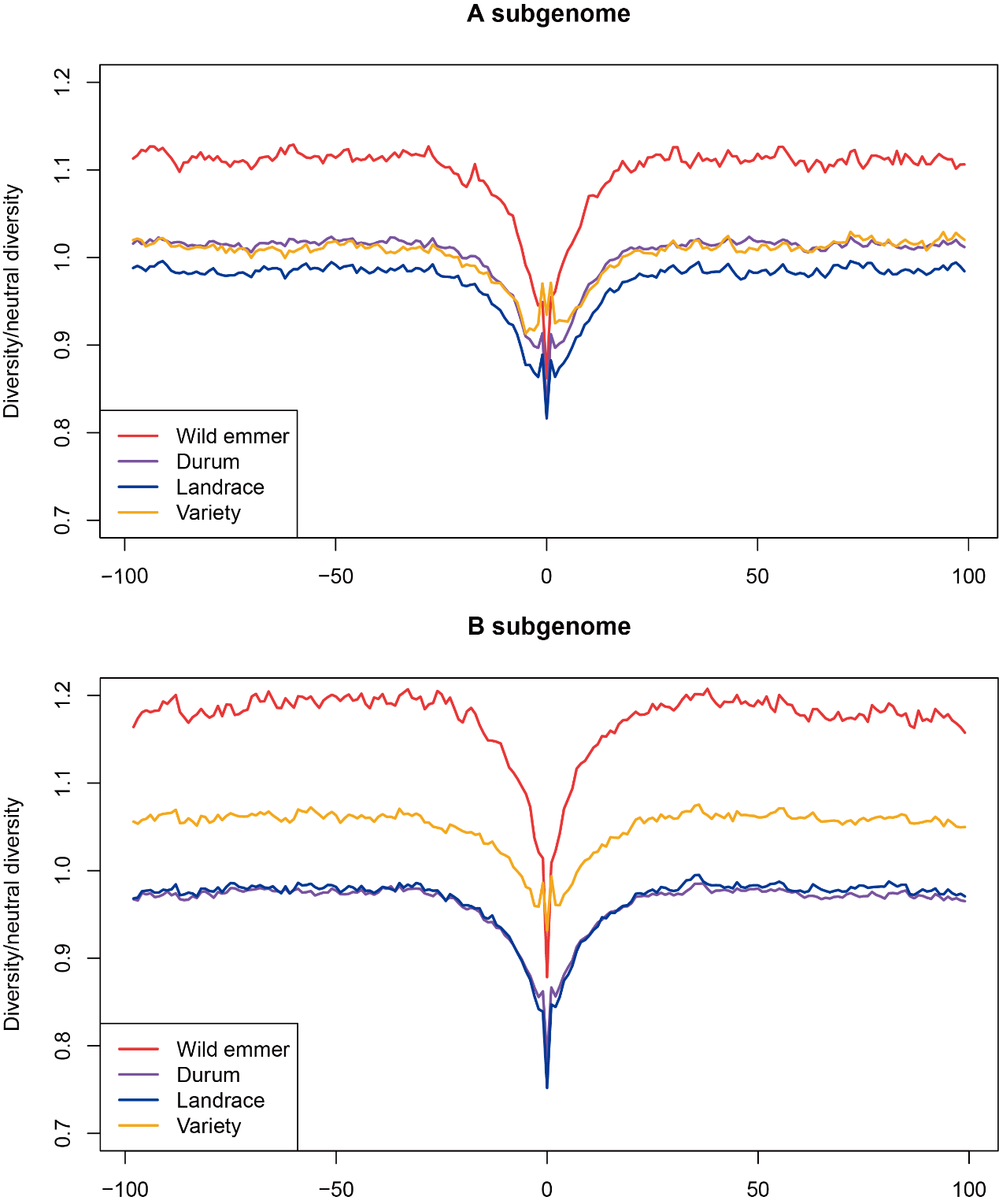
**Figure S3: The relative diversity of A and B subgenomes for wild emmer, durum, landrace and variety wheat.** The diversity was calculated in non-overlapped 1kb window. Relative diversity is calculated compared to the mean diversity of windows with distance >50kb from the nearest gene


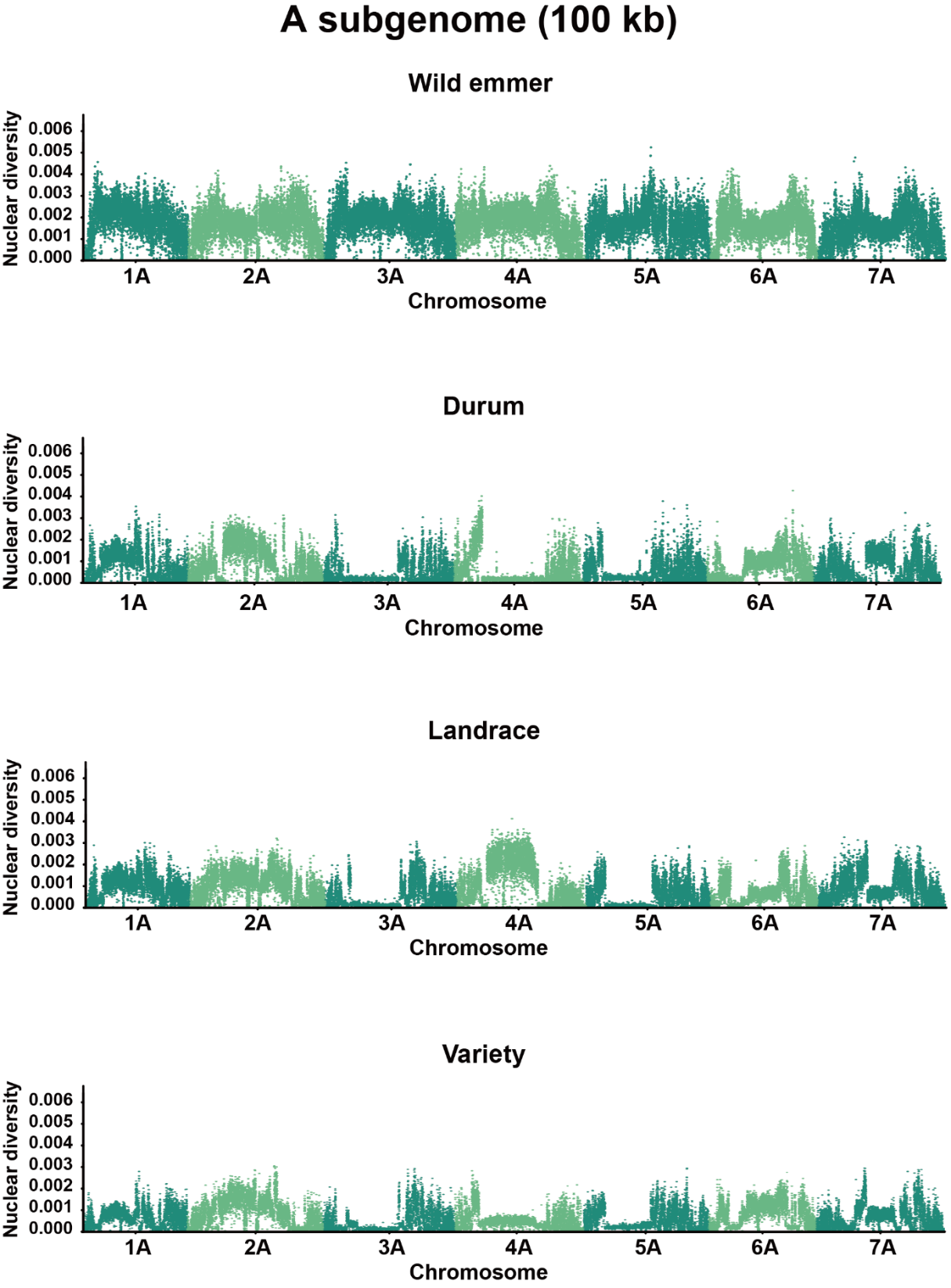


**Figure S4: Nucleotide diversity (π) of the A subgenome of wild emmer, durum, the landraces, and the varieties.** The π value were calculated in non-overlapping 100 kb window.


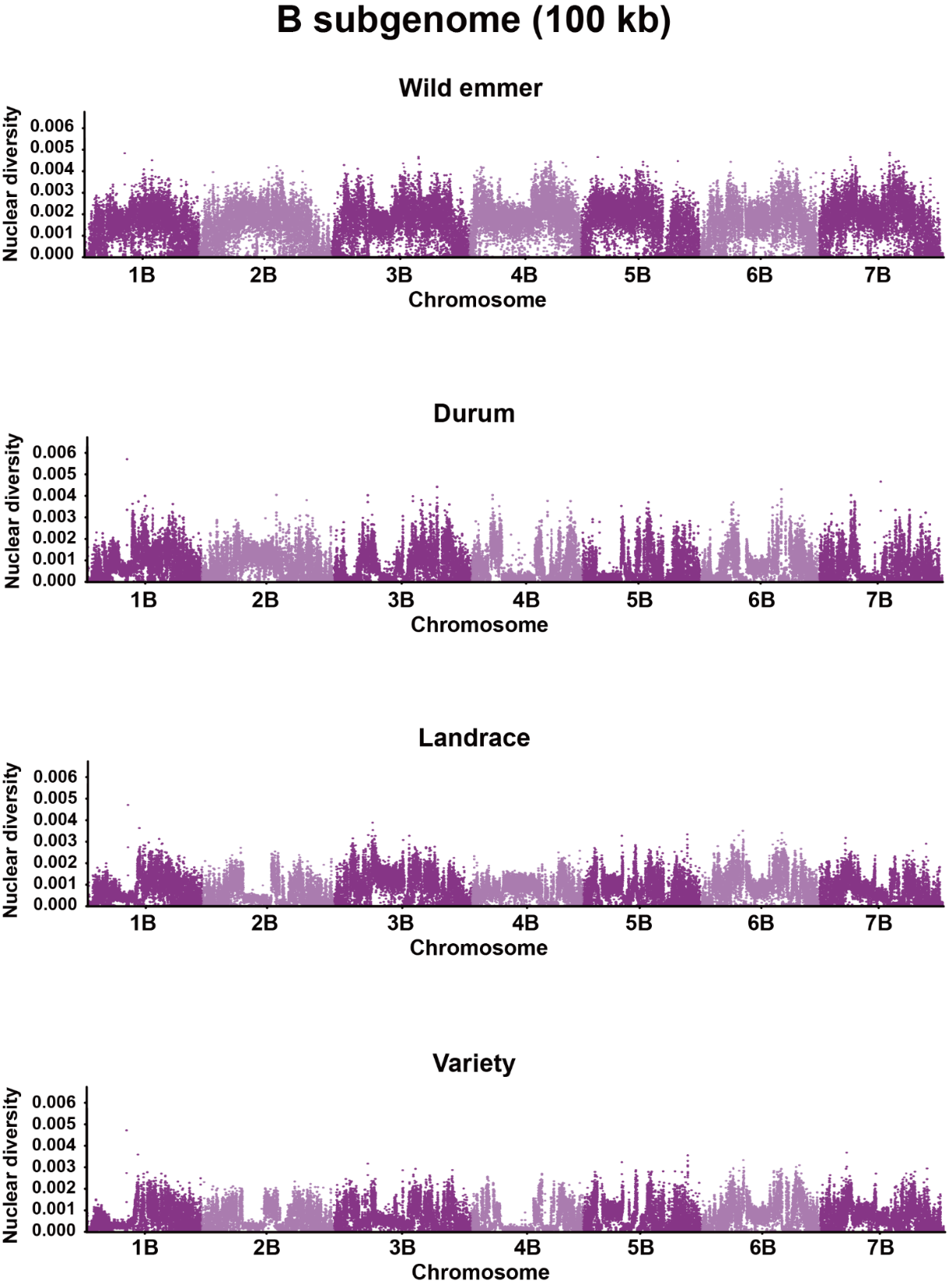


**Figure S5: Nucleotide diversity (π) of the B subgenome of wild emmer, durum, the landraces, and the varieties.** The π value were calculated in non-overlapping 100 kb window.


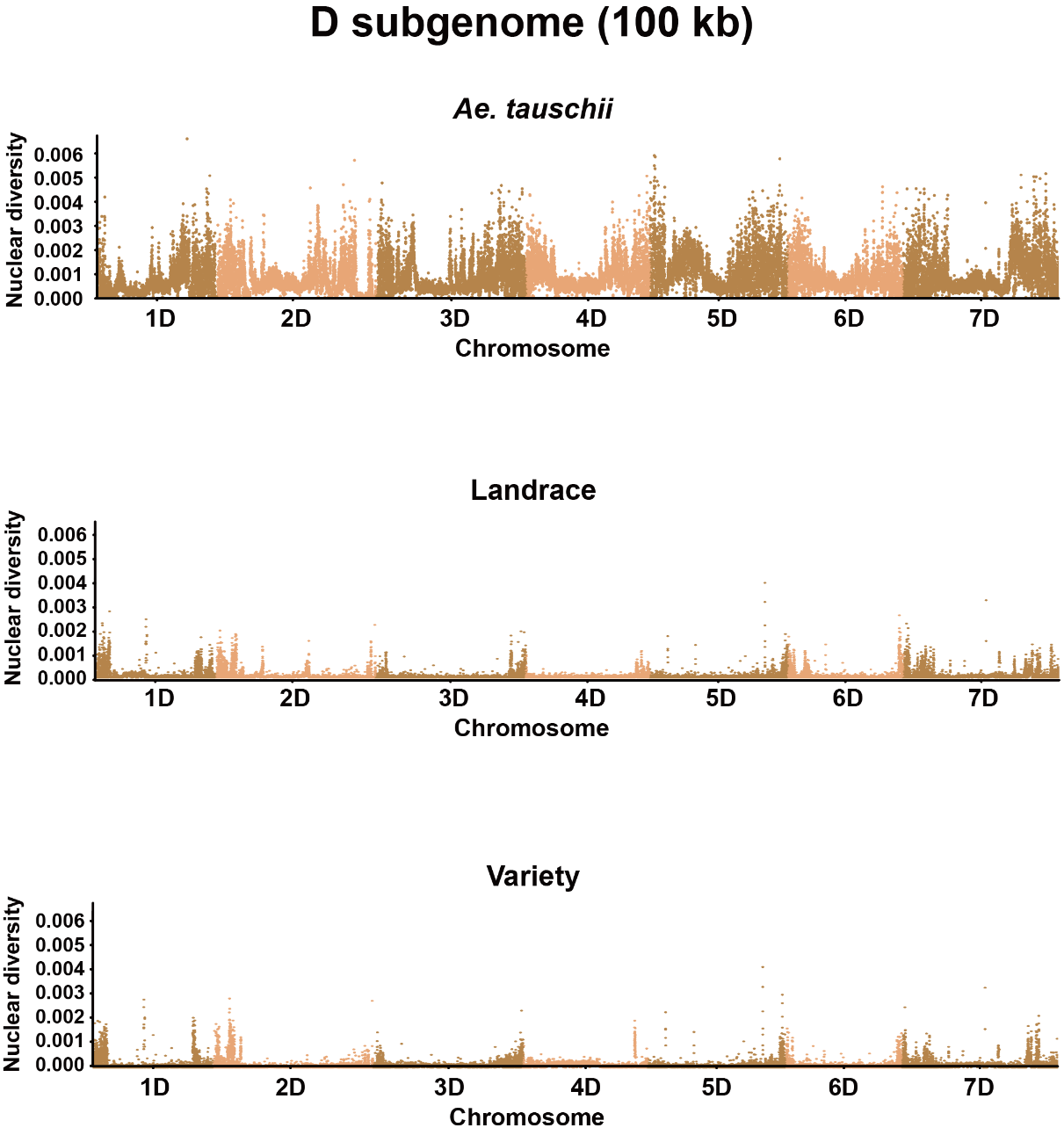


**Figure S6: Nucleotide diversity (π) of the D subgenome of *Ae. tauschii*, the landraces, and the varieties.** The diveristy of D subgenome are extremely low in landrace and variety.


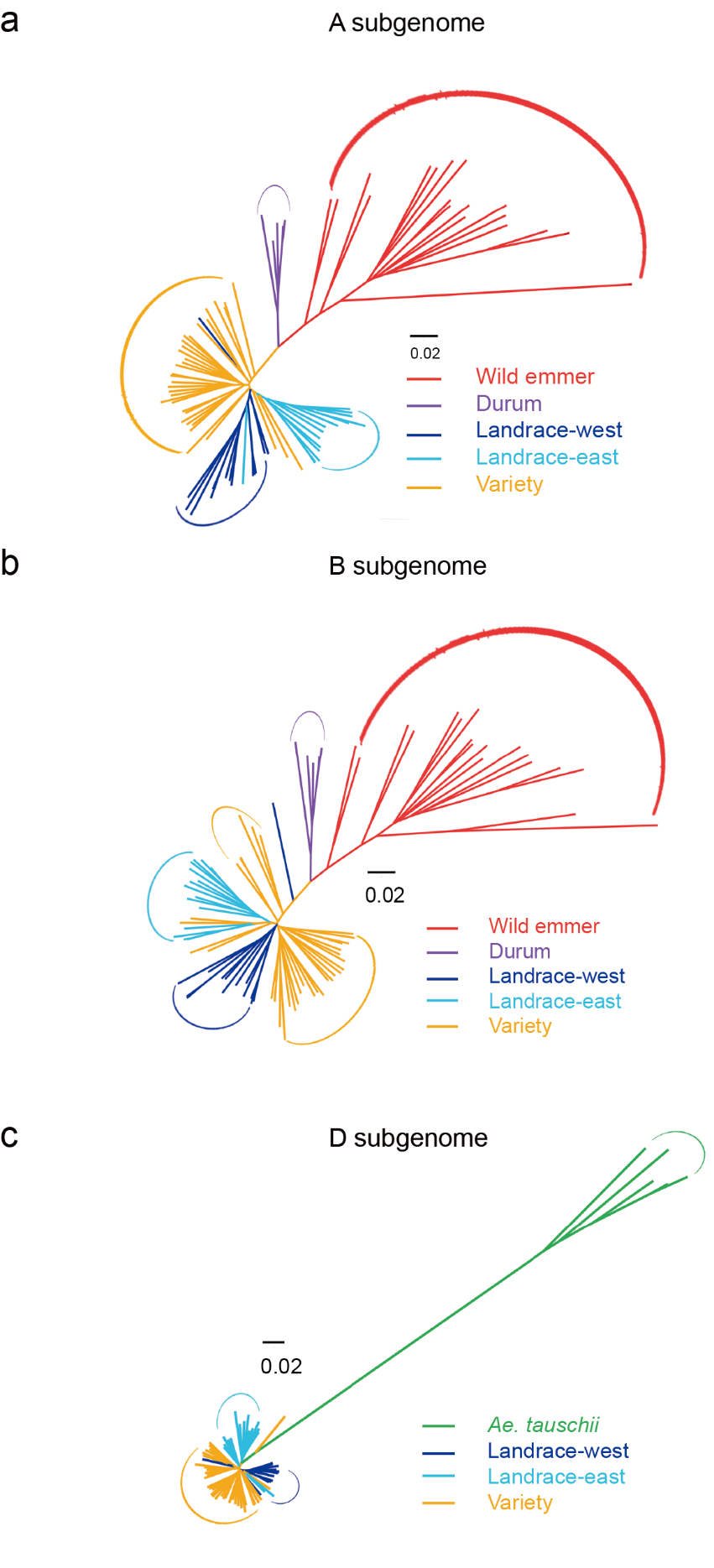


**Figure S7: Neighbour-joining (NJ) trees of 93 accessions on the A, B and D subgenomes.** Branch colours reflect different populations.

**
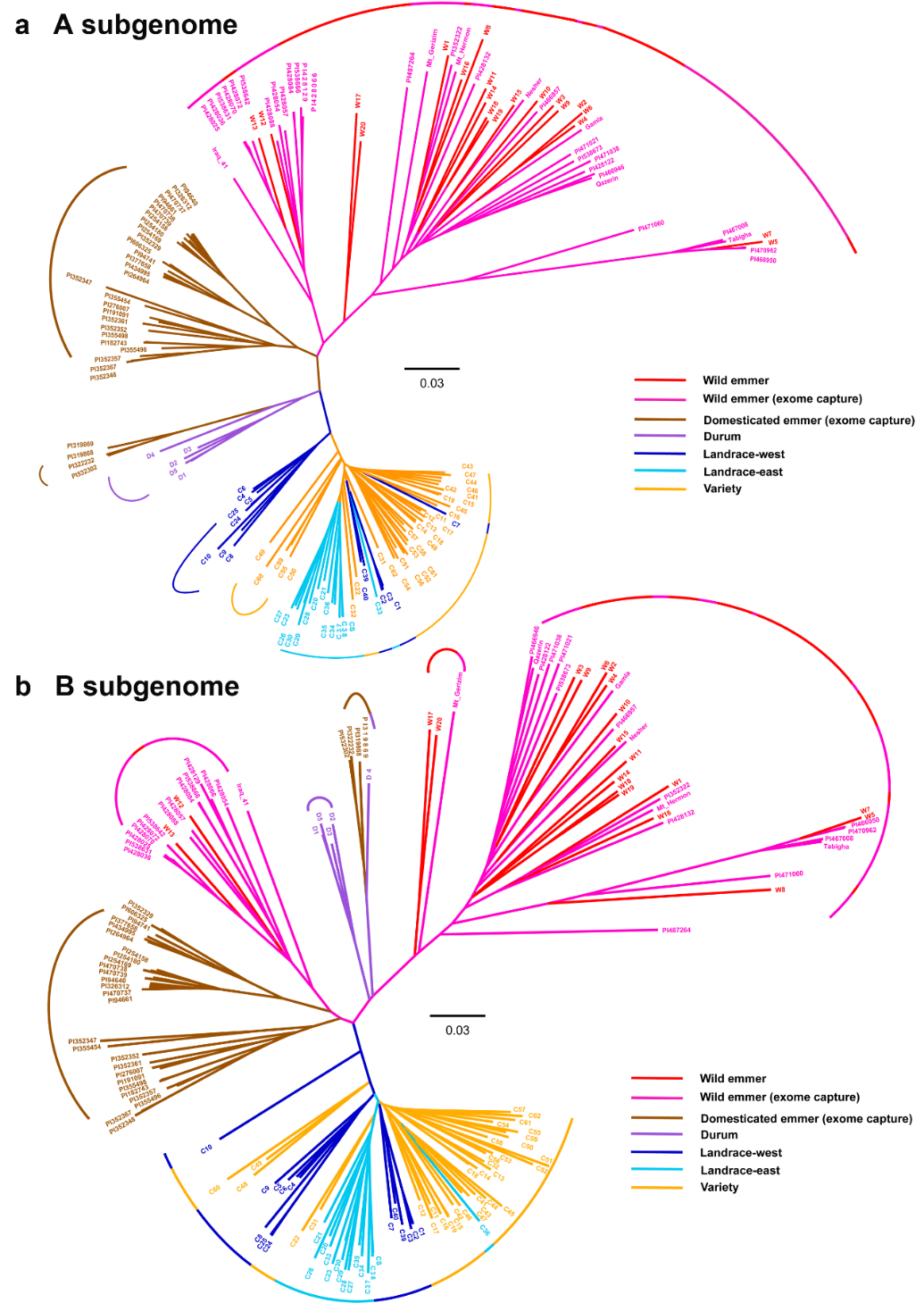
**

**Figure S****8: Neighbour-joining (NJ) trees of 152 accessions on the A and B subgenomes.** Among 152 accessions, 33 wild emmer and 31 domesticated emmer accessions were generated from exome capture by previous study [[1](#_ENREF_1)]. A total of 151,672 and 161,280 SNPs were used to construct the trees for A and B subgenomes, respectively. Branch colours reflect the populations. Wild emmer clustered into four clades in both A and B subgenomes, as shown in (a) and (b), respectively, indicating the internal population structure within wild emmer. Population information is provided in Table S1 and table S5.


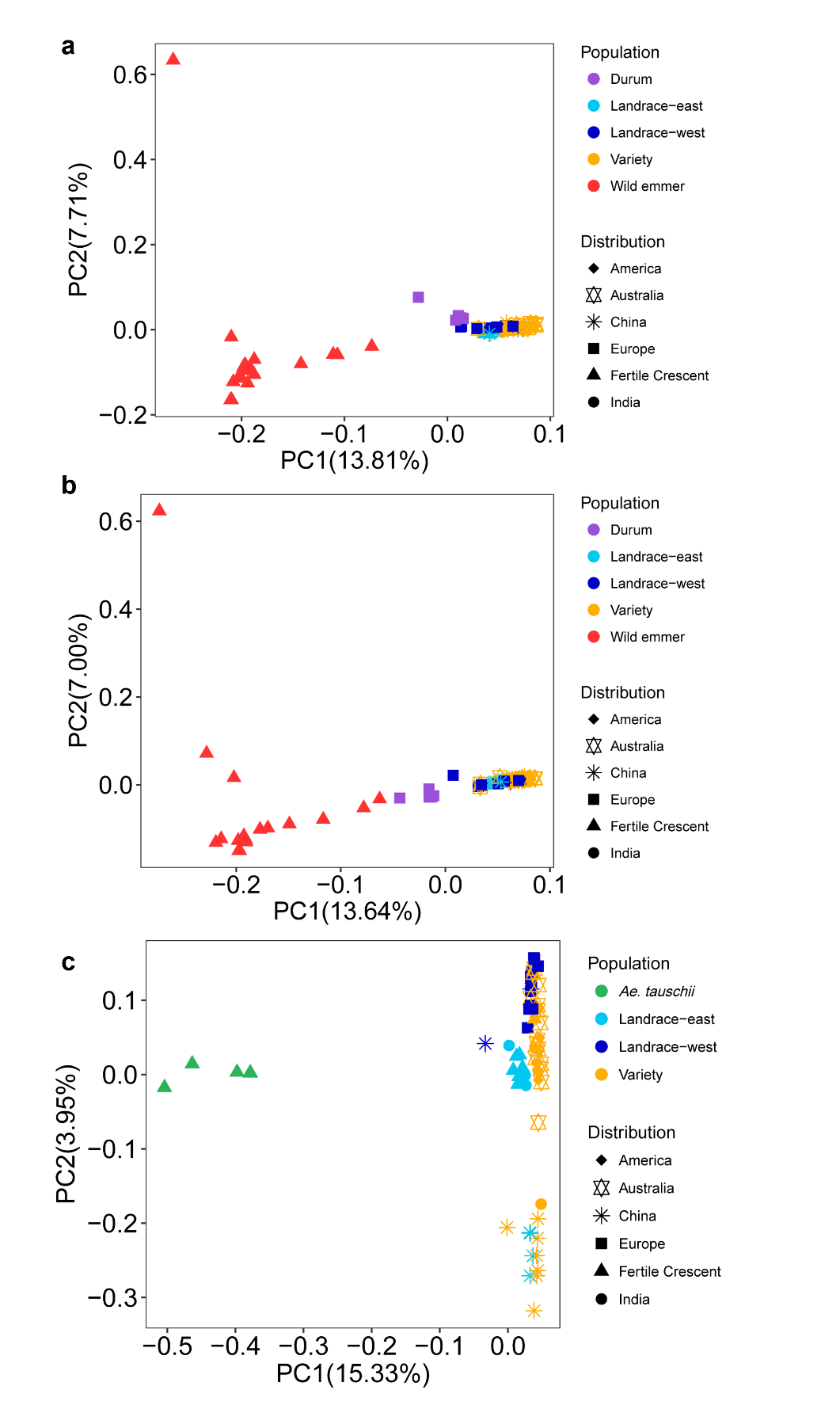


**Figure S9: Principal component analysis of all accessions in the A, B, and D subgenomes.** Different shapes and colours represent different geographical locations and populations, respectively. The first component was driven by the difference among poulations in all the three subgenomes (a, b and c). In D subgenome, a clear separation was found between the Chinese variety and the landraces and other accessions along the second component (c).


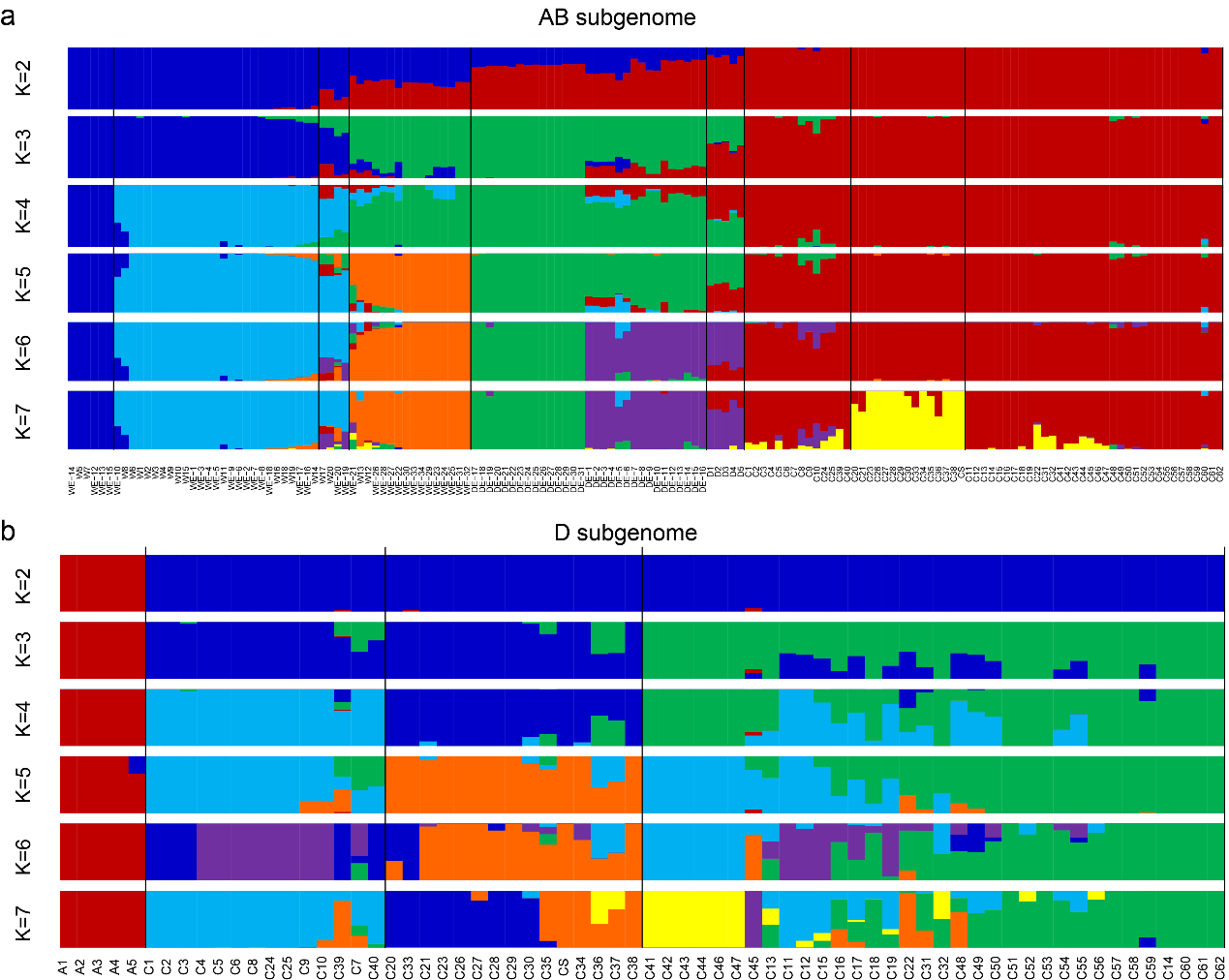


**Figure S10: Population structure of AB and D subgenomes using ADMIXTURE.** Each colour represents an ancestral population. The length of each segment in each vertical bar represents the proportion contributed by ancestral populations. The K (the number of ancestral populations) is set from 2 to 7 in the AB and D subgenomes in (a) and (b), respectively.


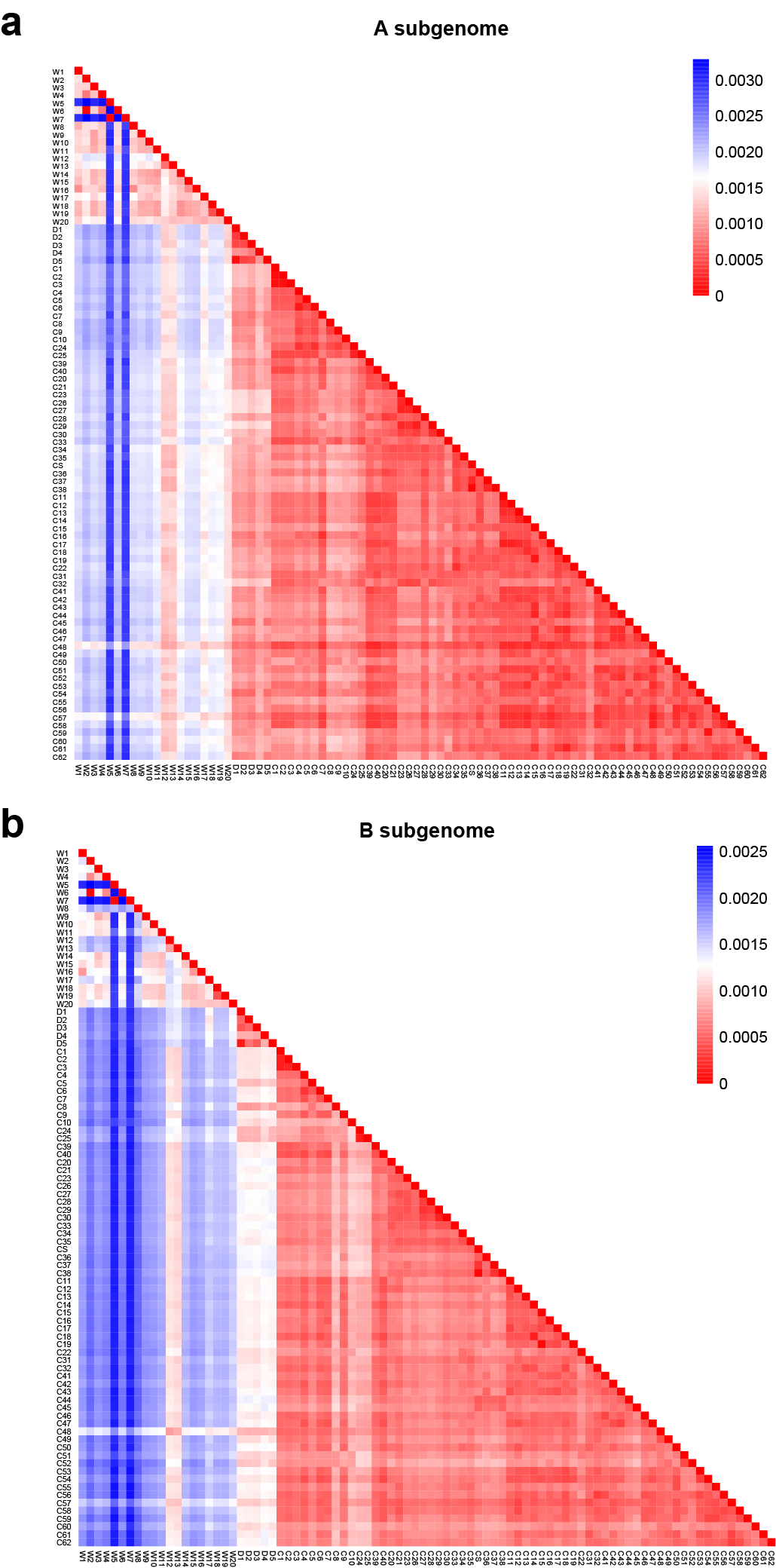


**Figure S11: Absolute sequence divergence dxy value (the number of pairwise differences per site) between each pairwise accessions on A and B subgenomes.**


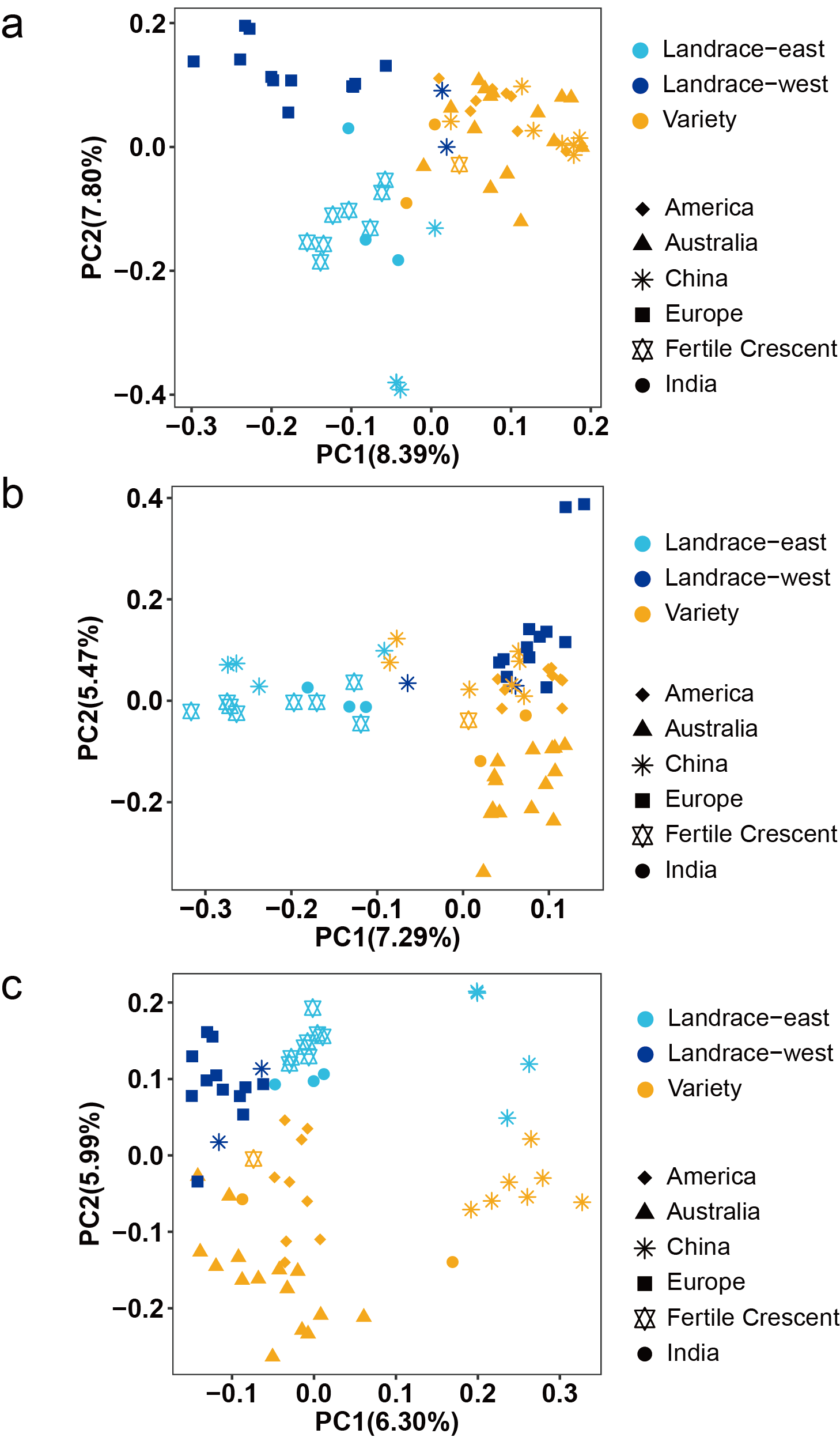


**Figure S12: Principal component analysis (PCA) plots based on the first two principal components of 63 bread wheat accessions on A, B, and D subgenomes.**


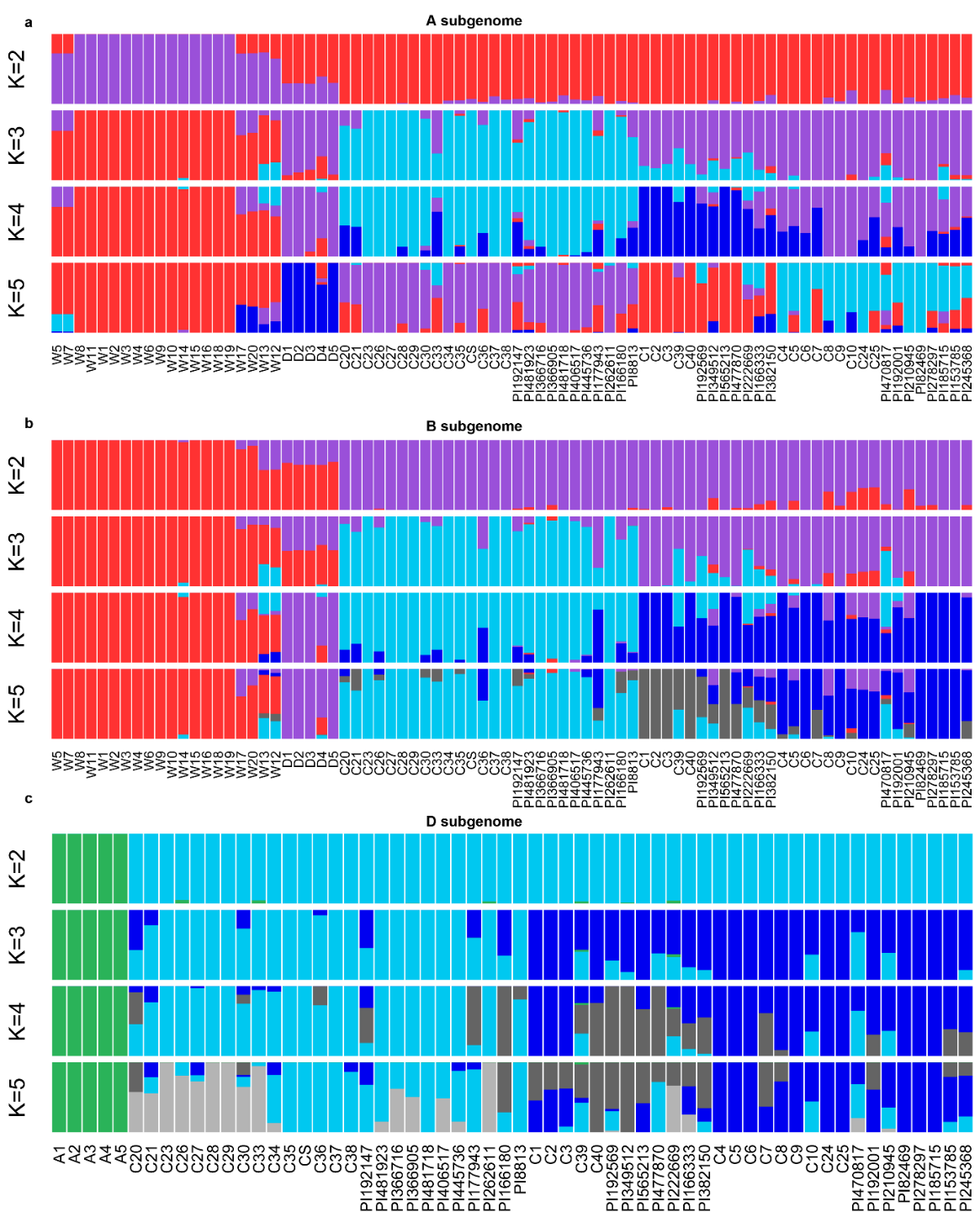


**Figure S13: ADMIXTURE analysis of 93 wheat accessions combined with the exon capture data of 26 landraces.** Each colour represents an ancestral population. The length of each segment in each vertical bar represents the proportion contributed by ancestral populations. The K (the number of ancestral populations) is set from 2 to 5 in the A, B, and D subgenomes in (a), (b) and (c), respectively. When K = 2, wild emmer and bread wheat are separated in the A and B subgenomes. When K = 3 and 4, the landraces is divided into two subgroups. The A, B, and D subgenome using an overlapping total of 40612, 53616, 12027 SNPs with exome capture data, respectively.


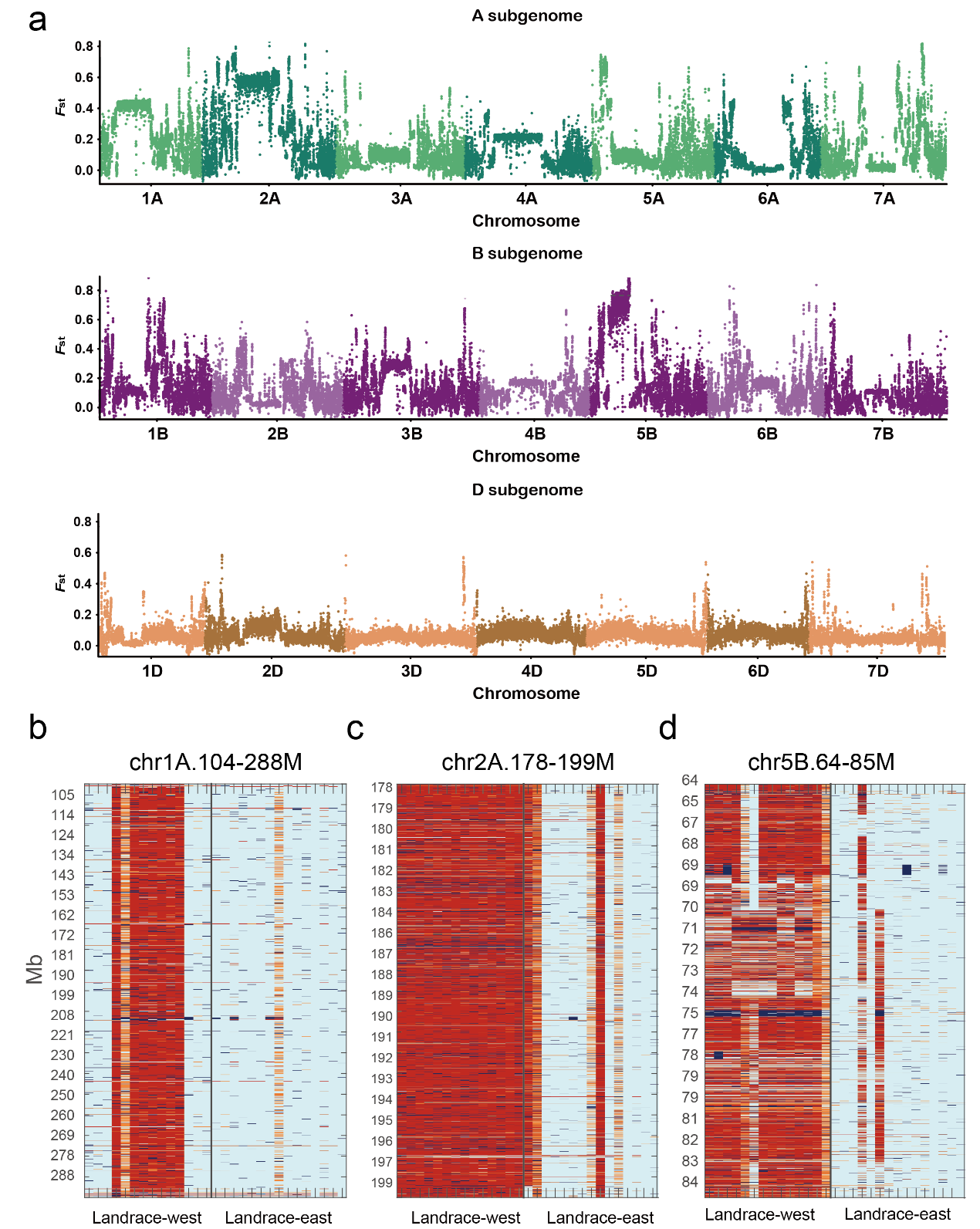


**Figure S14: (a) Population divergence(Fst) of A, B and D subgenome.** **(c), (d) and (e) represents the patterns of SNPs of the high regions with long stretches of elevated Fst on chromosome 1A, 2A, and 5B.** Each column is an accession, each row is SNP site. Different colors donate the genotypes of SNPs, light blue for reference homozygous sites, red for homozygous nonreference sites, orange for heterozygous site, and navy for missing.


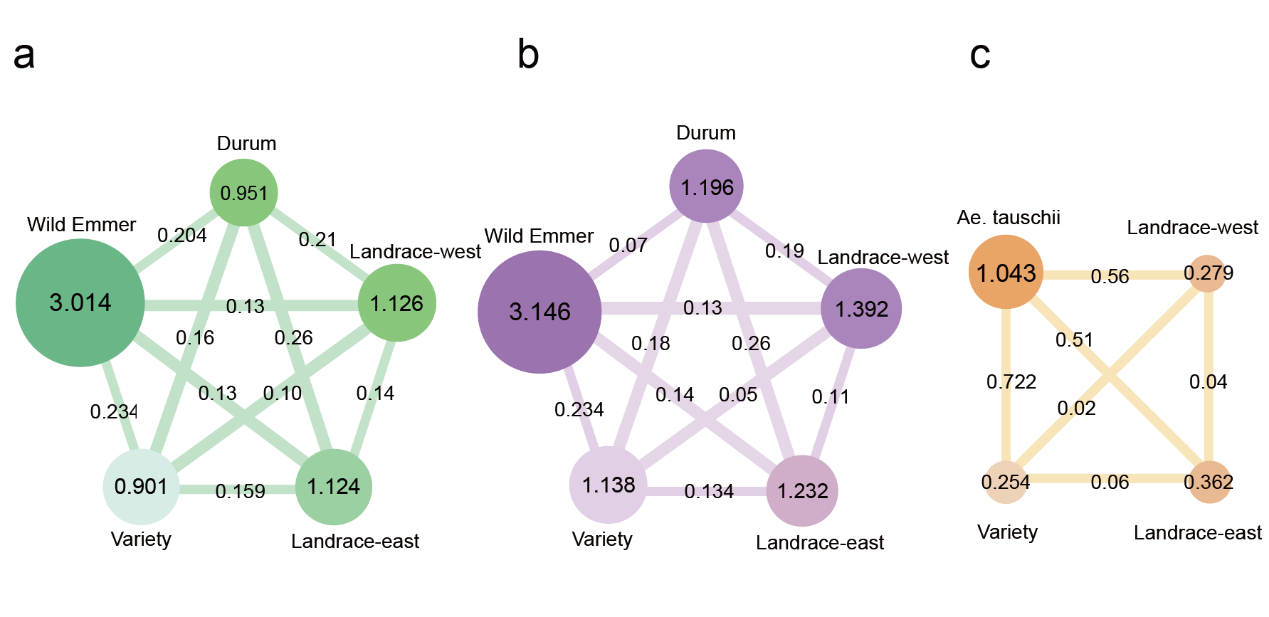
**Figure S15: Nucleotide diversity (π) and population divergence (*F*_ST_) across five or four groups.** The value in each circle represents the measure of nucleotide diversity (π×10^3^), and the value on each line indicates population divergence between two groups.


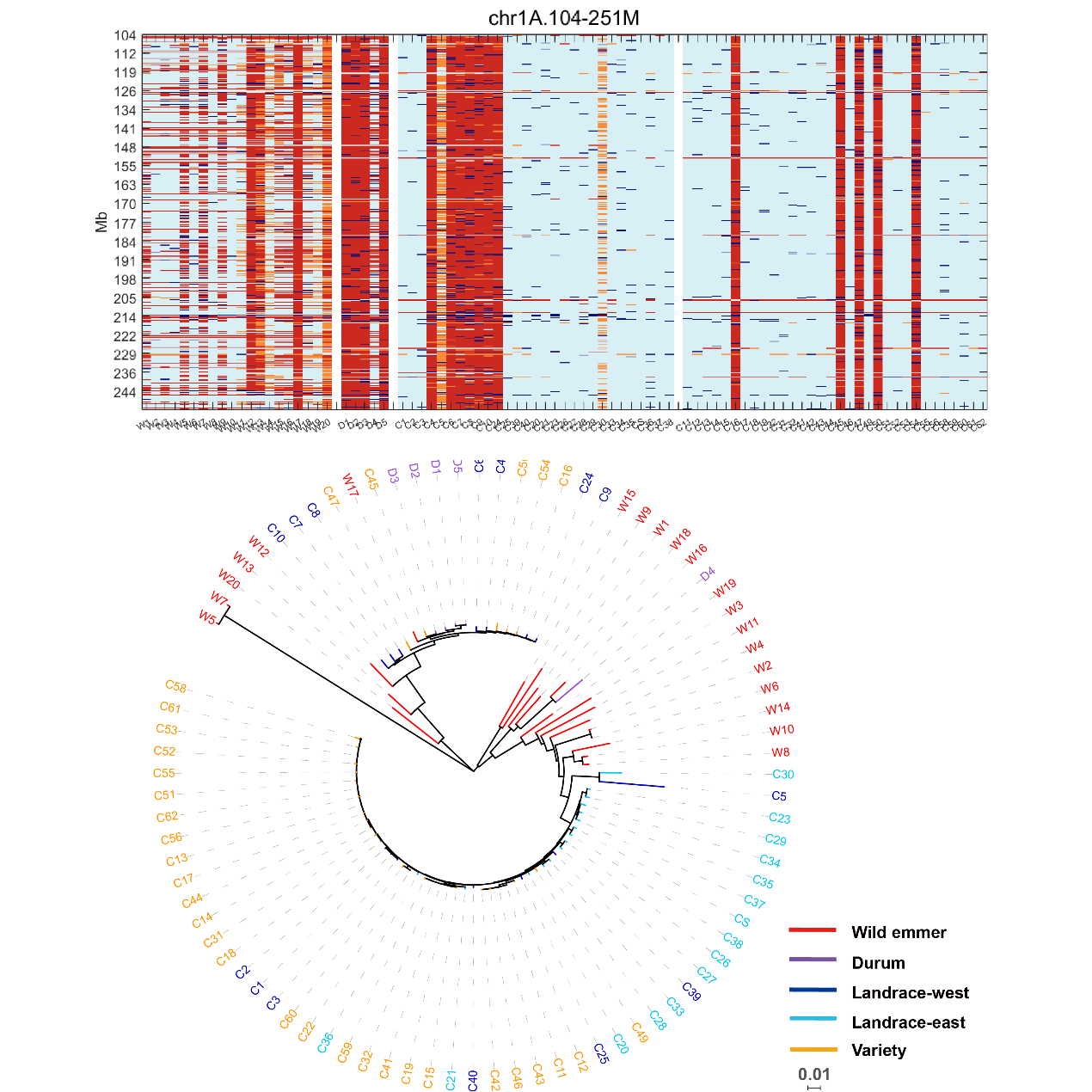


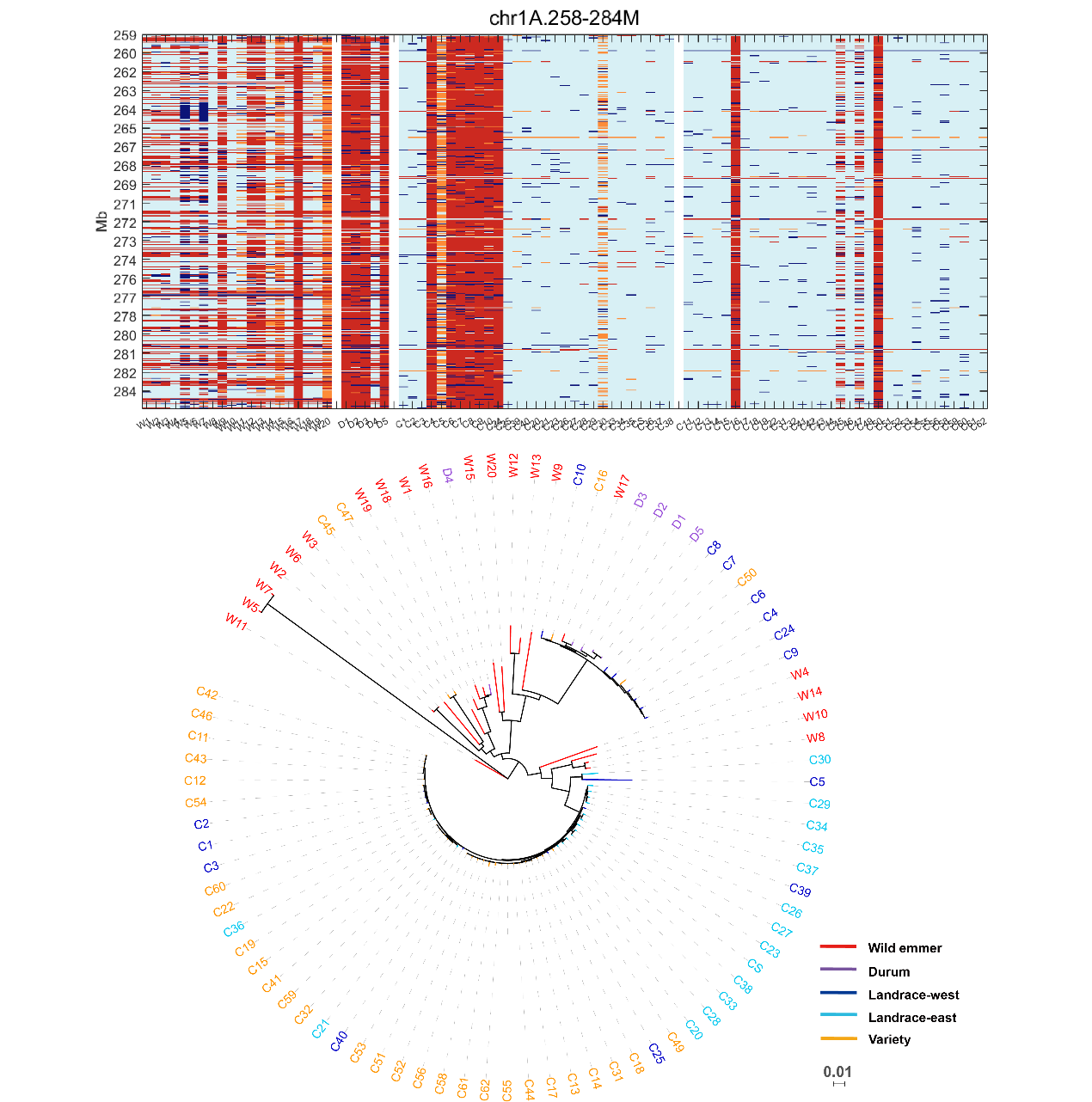


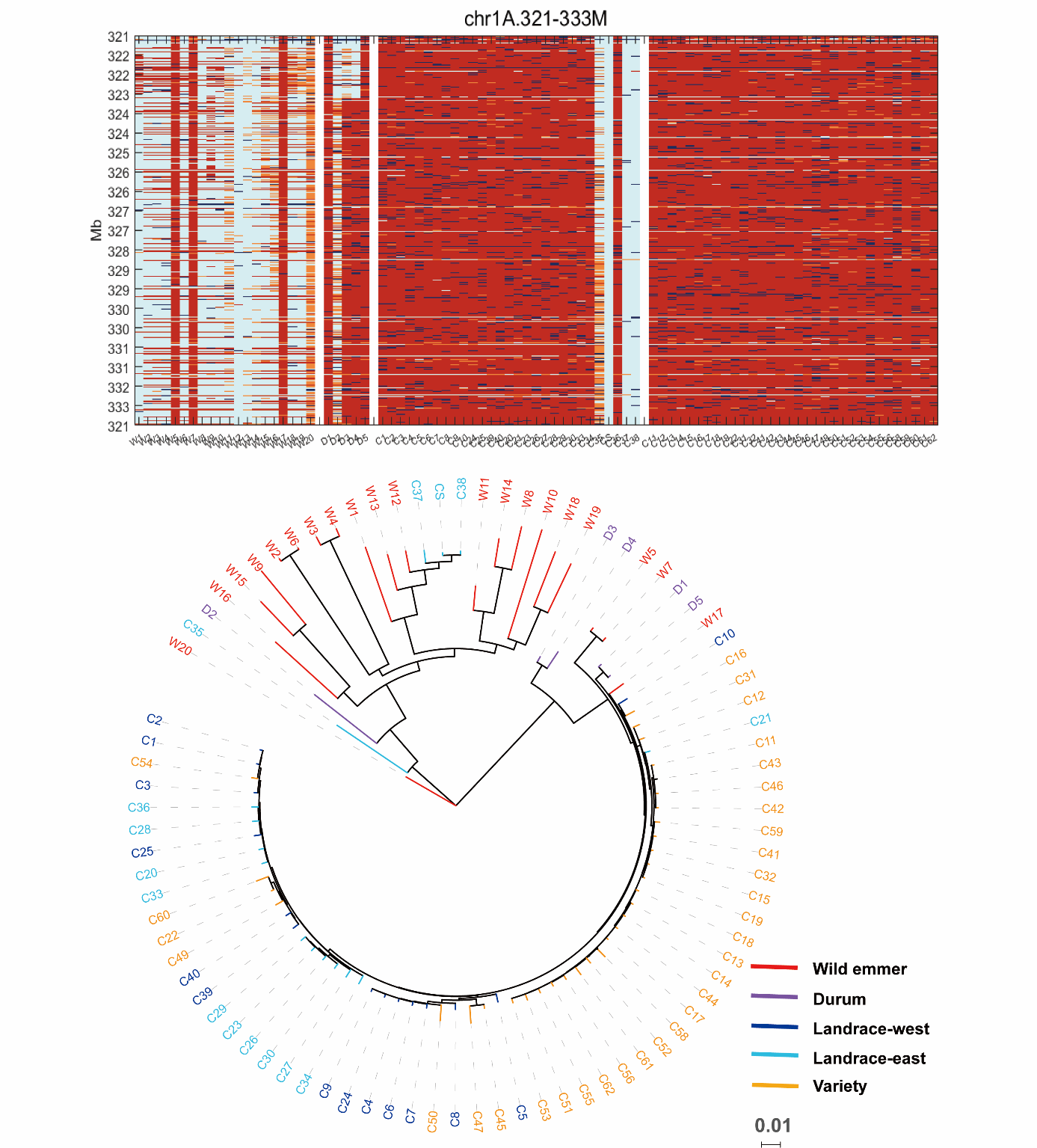


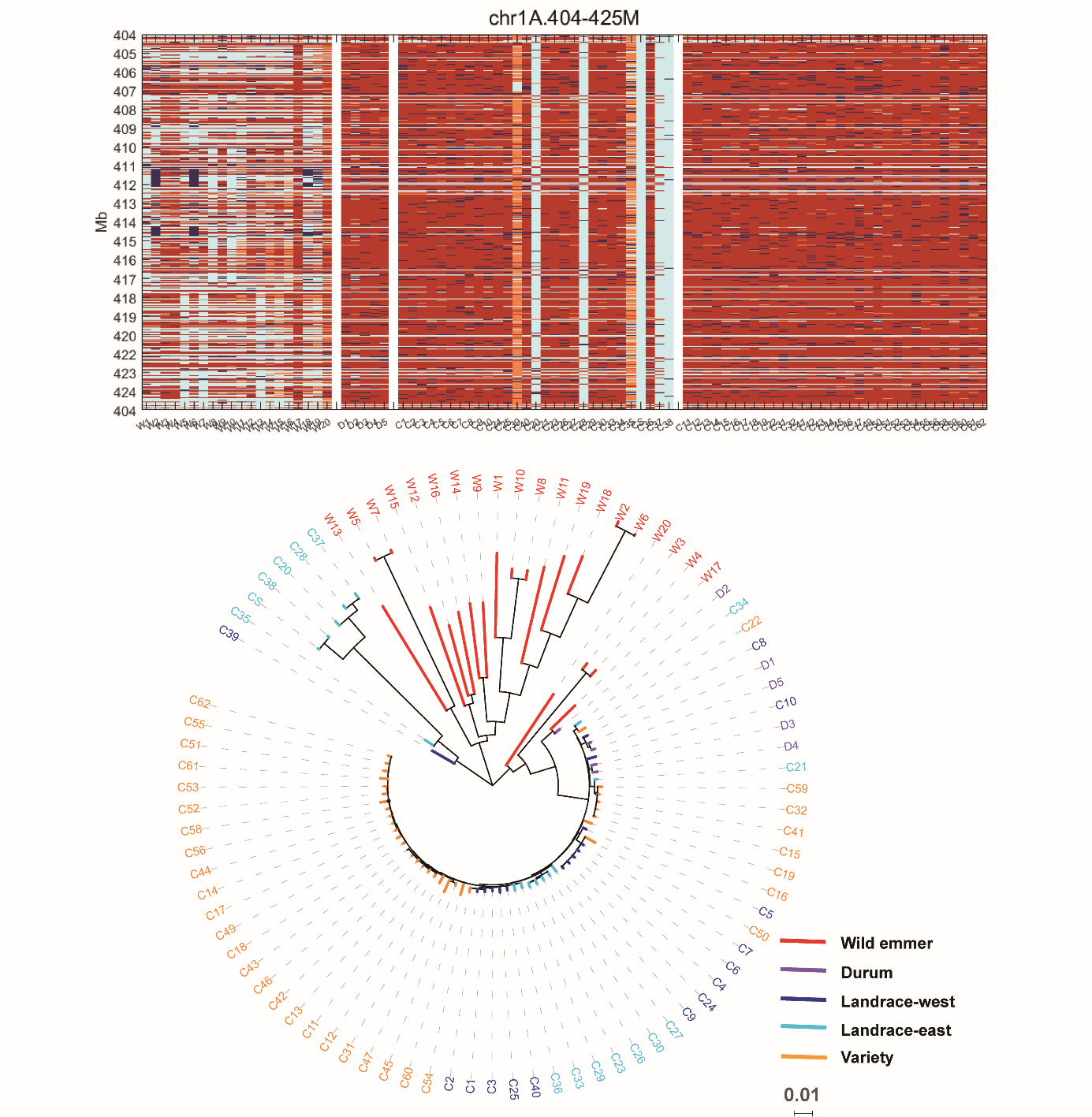


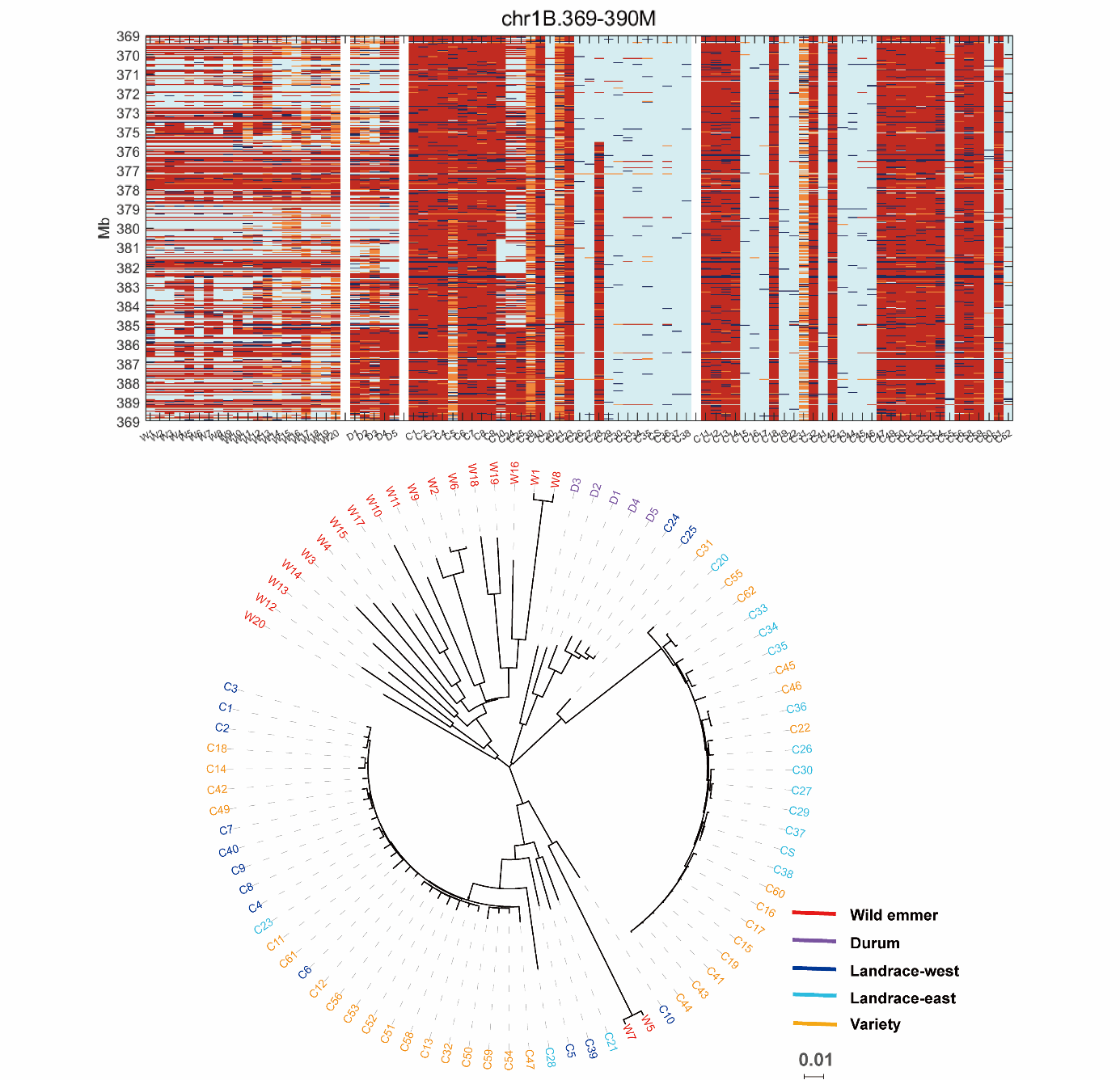


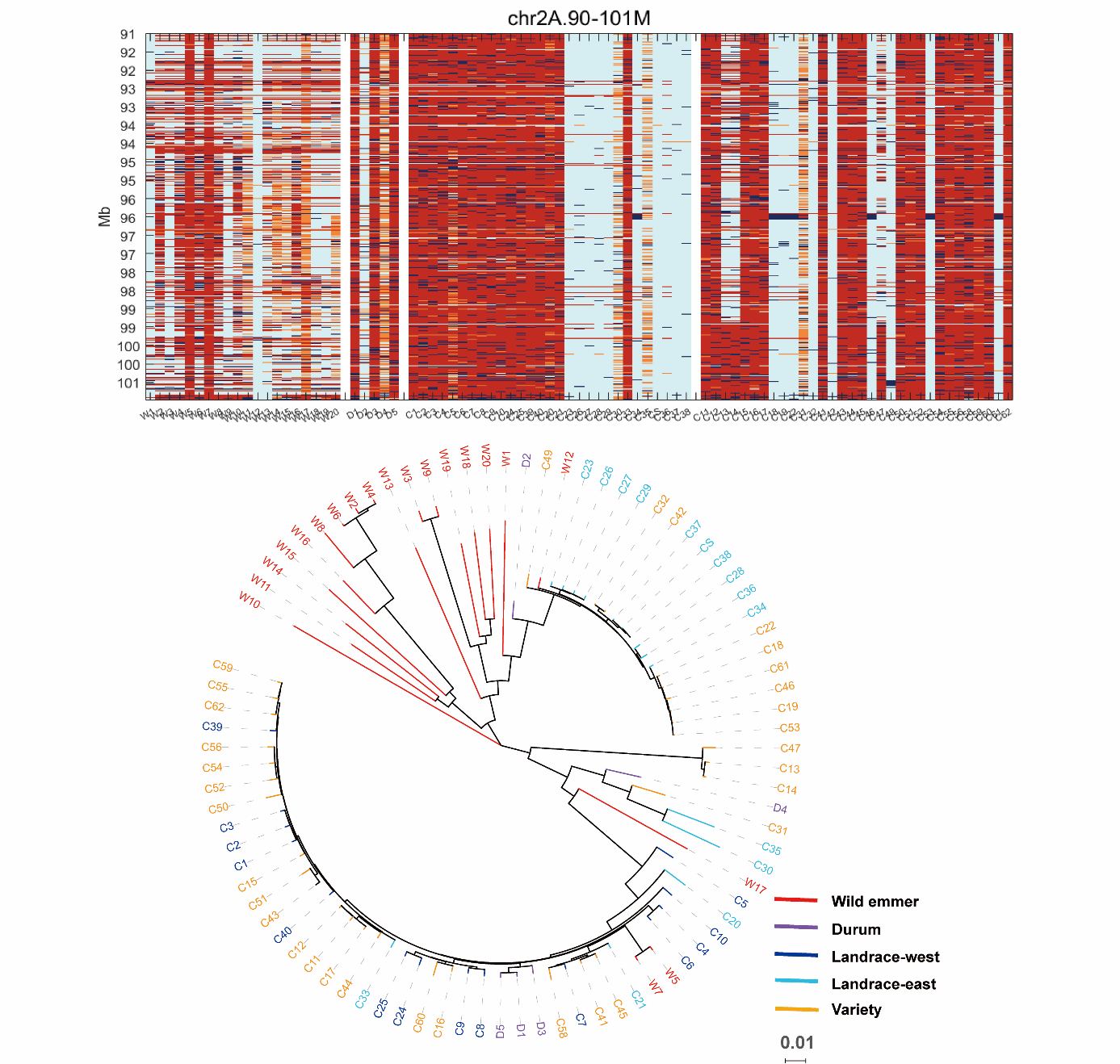


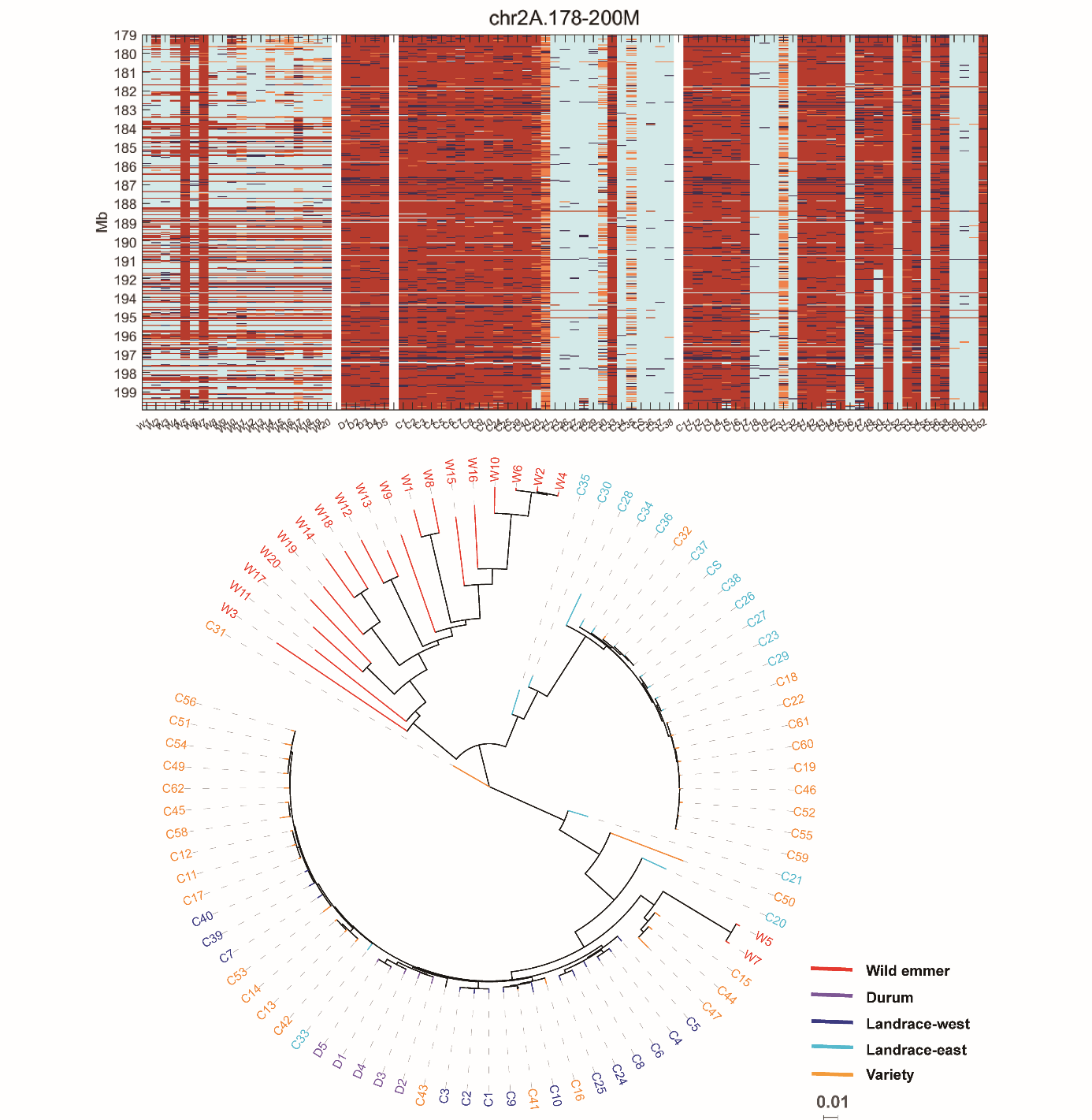


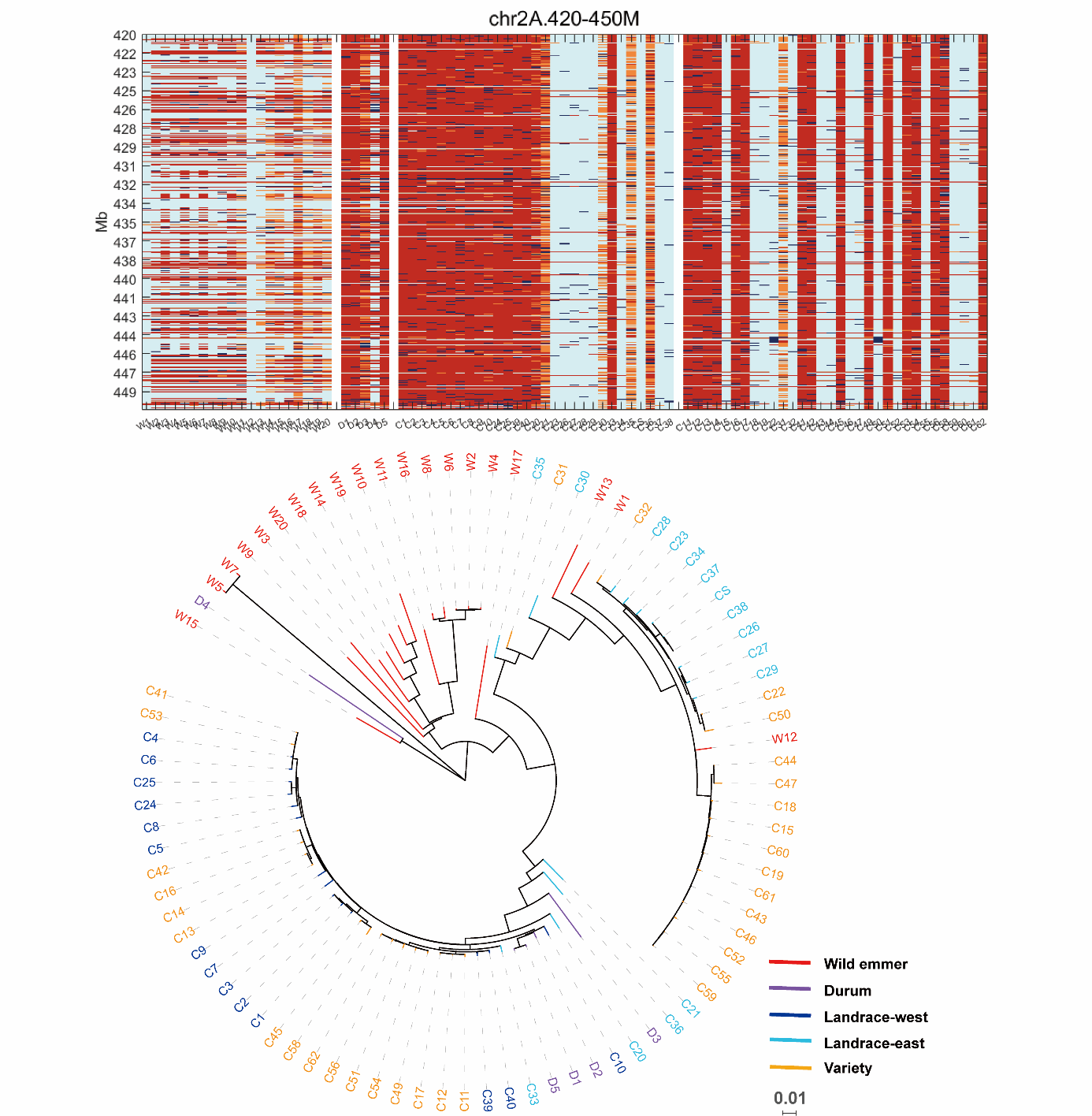


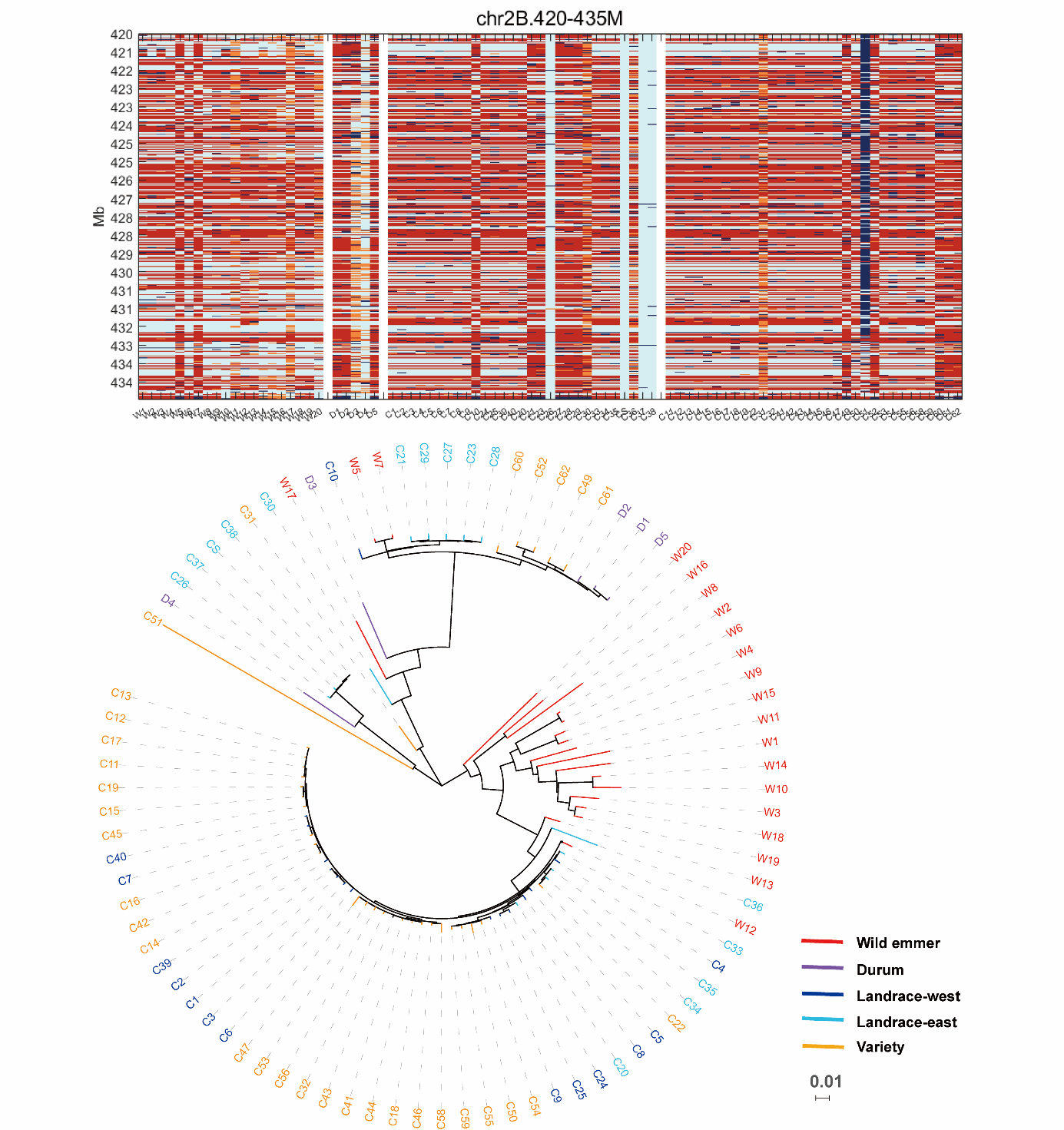


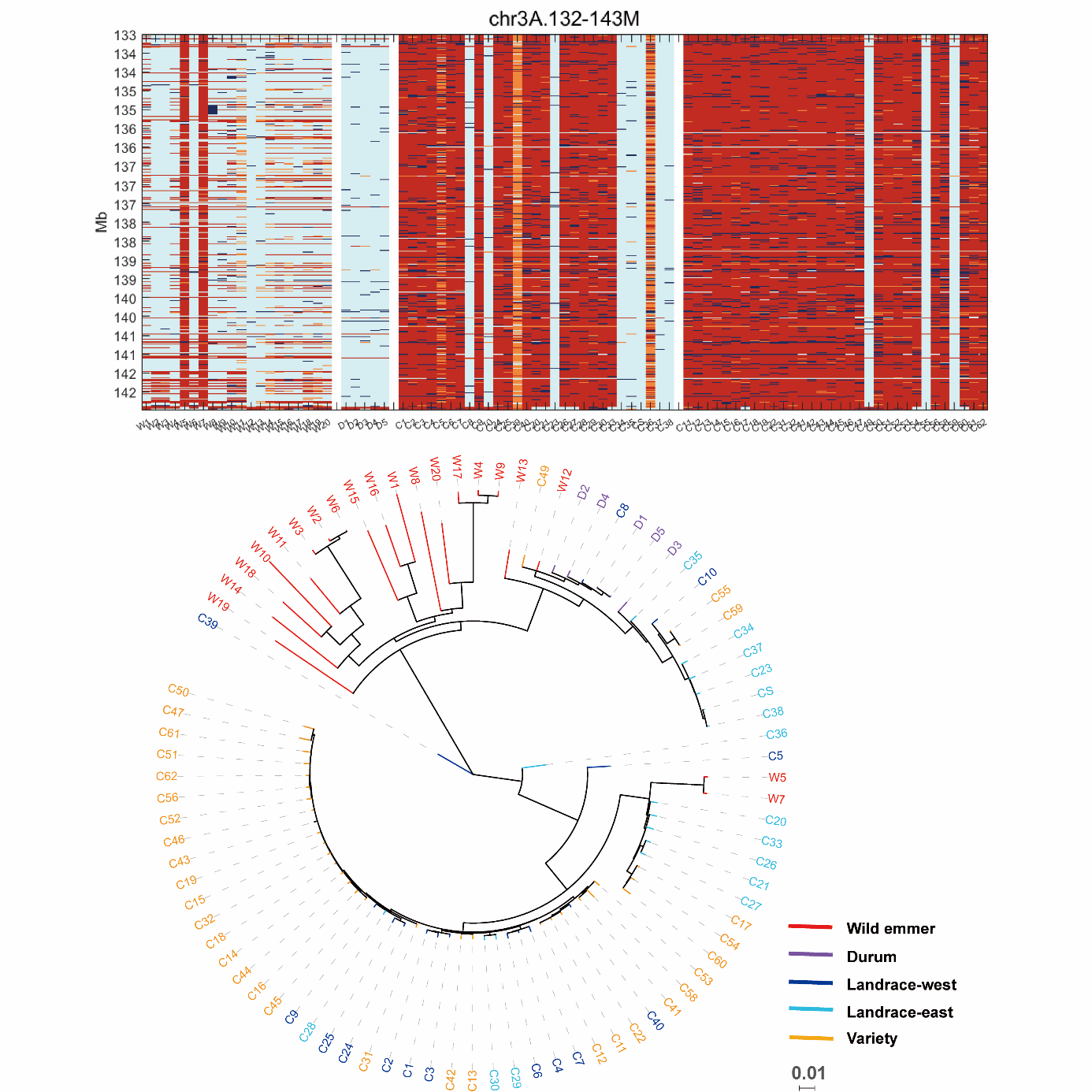


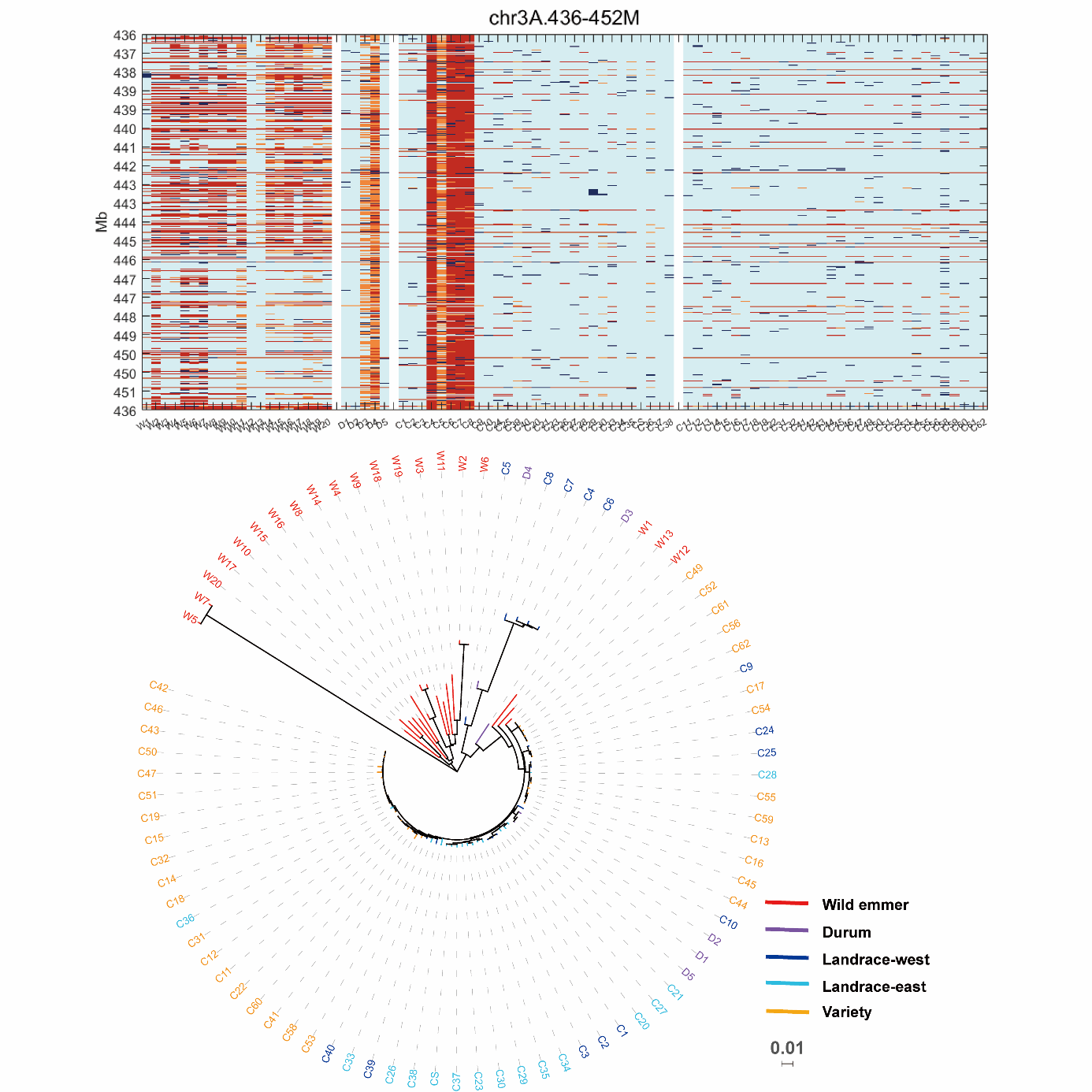


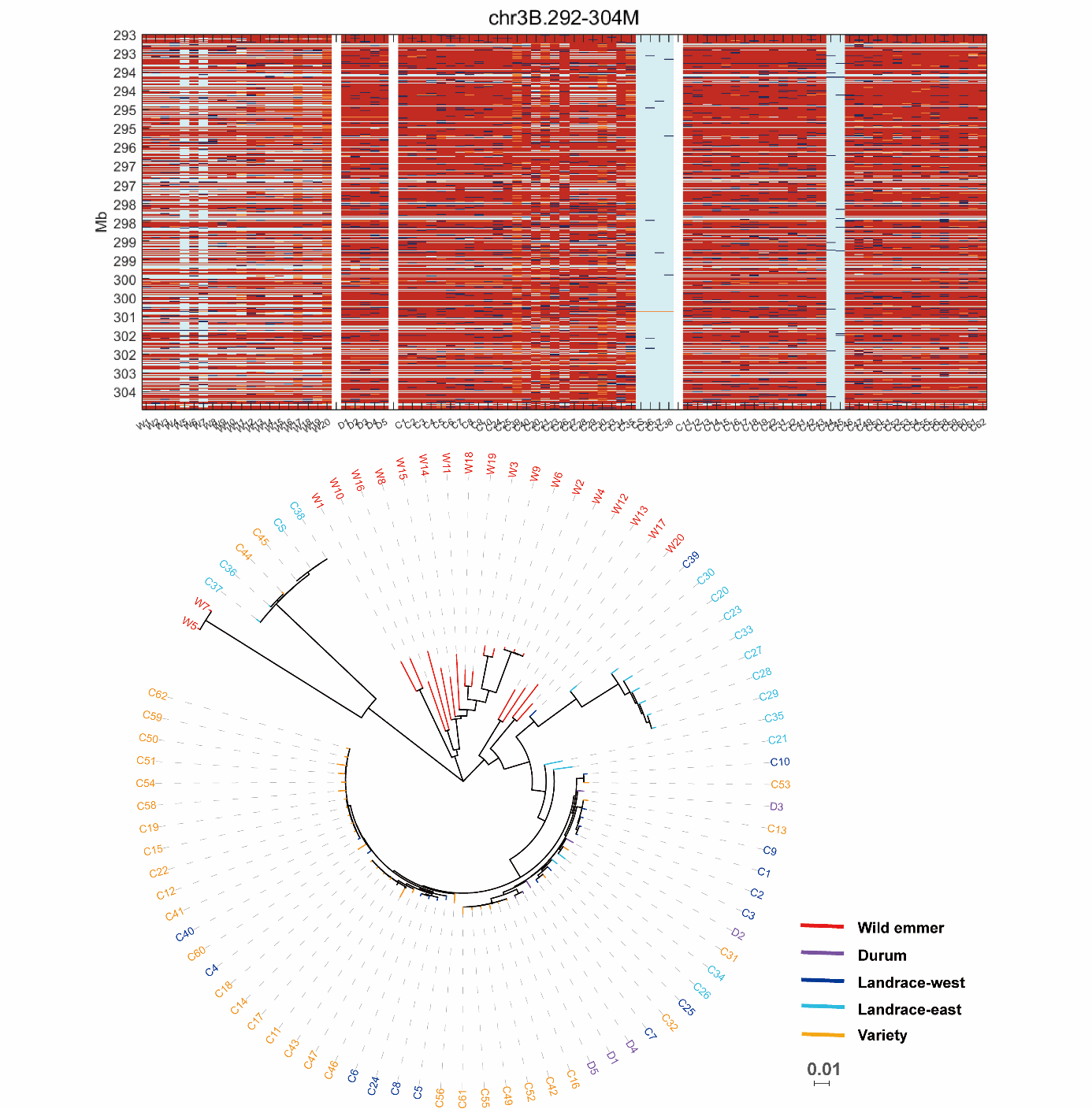


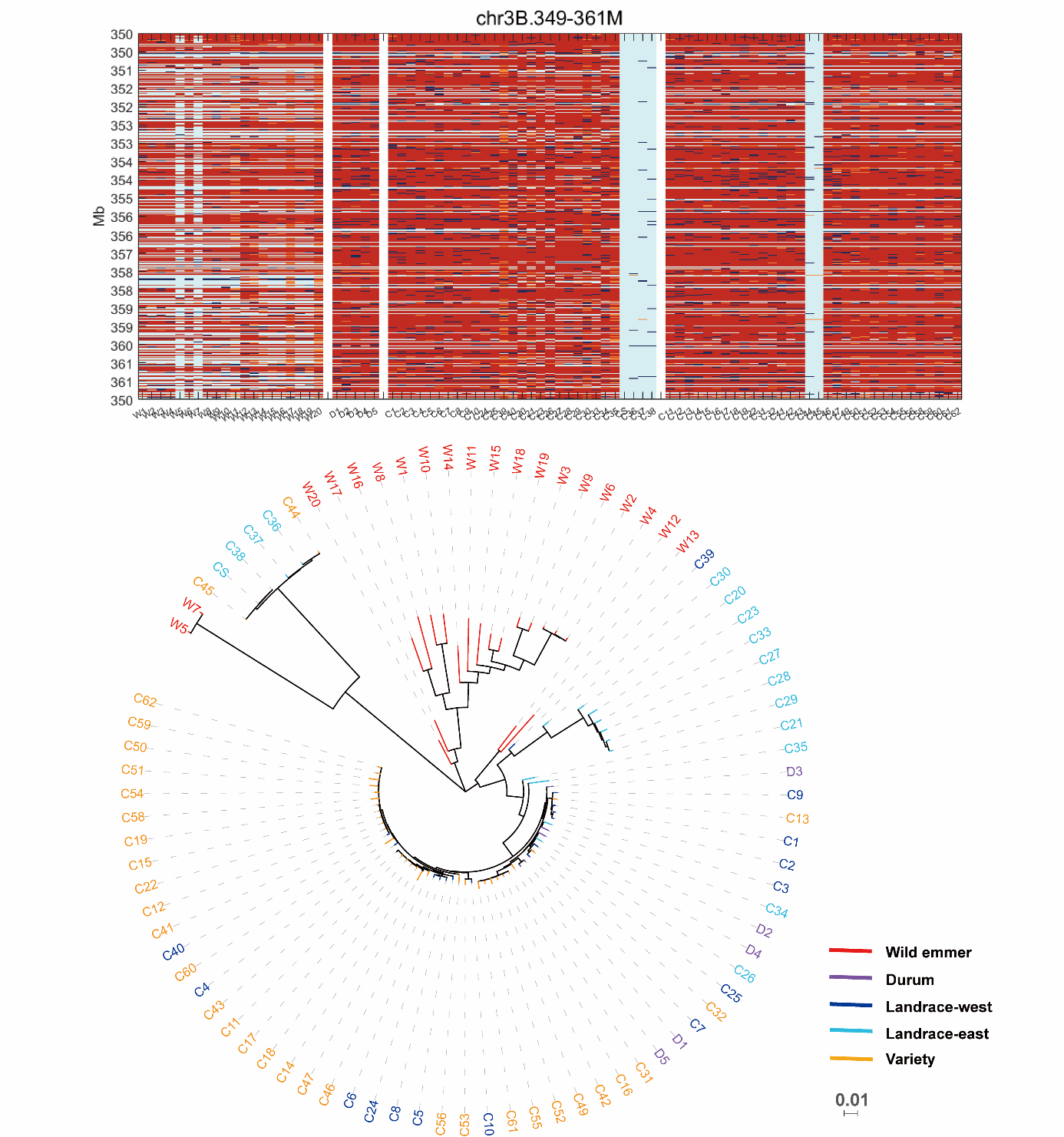


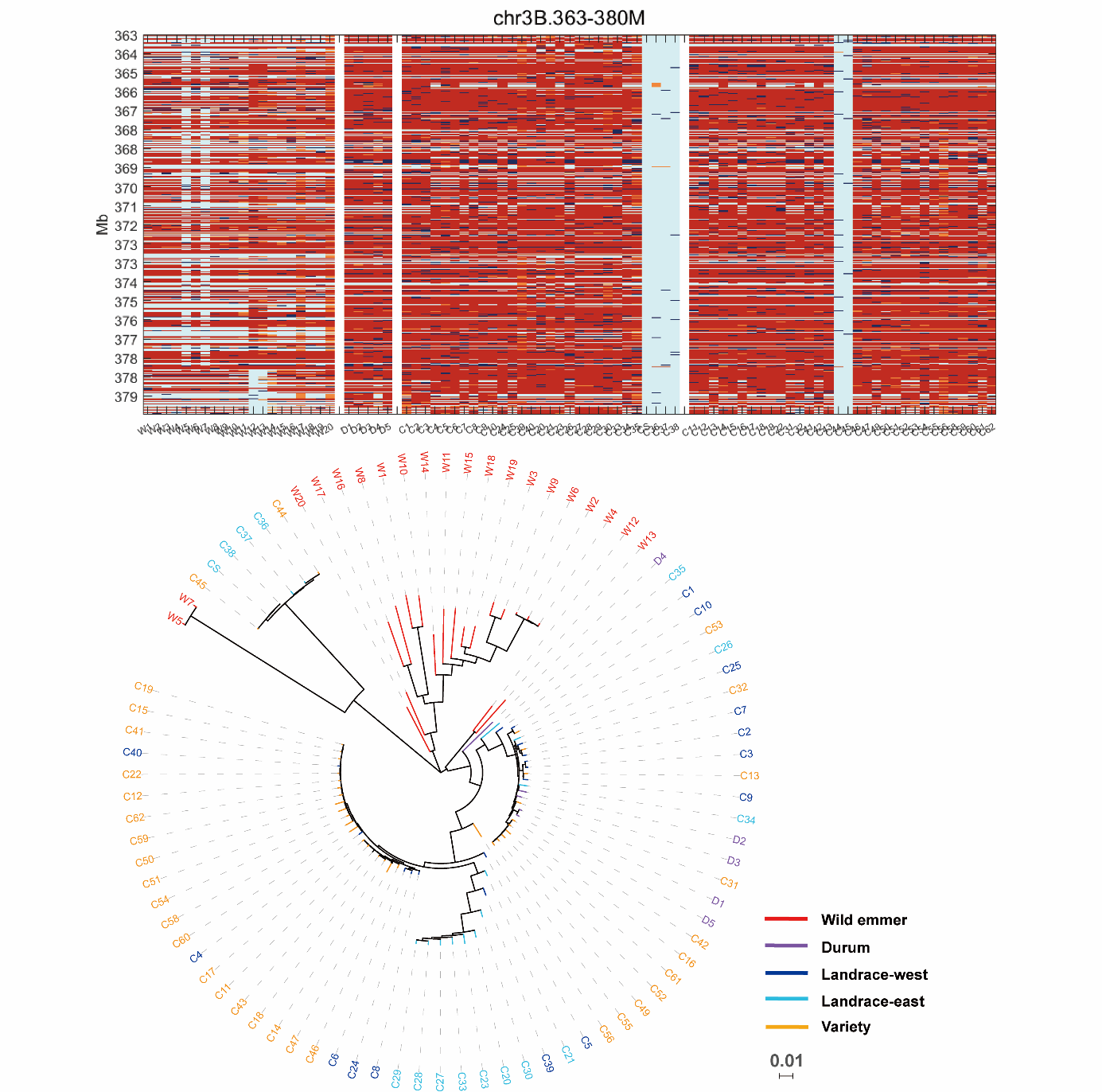


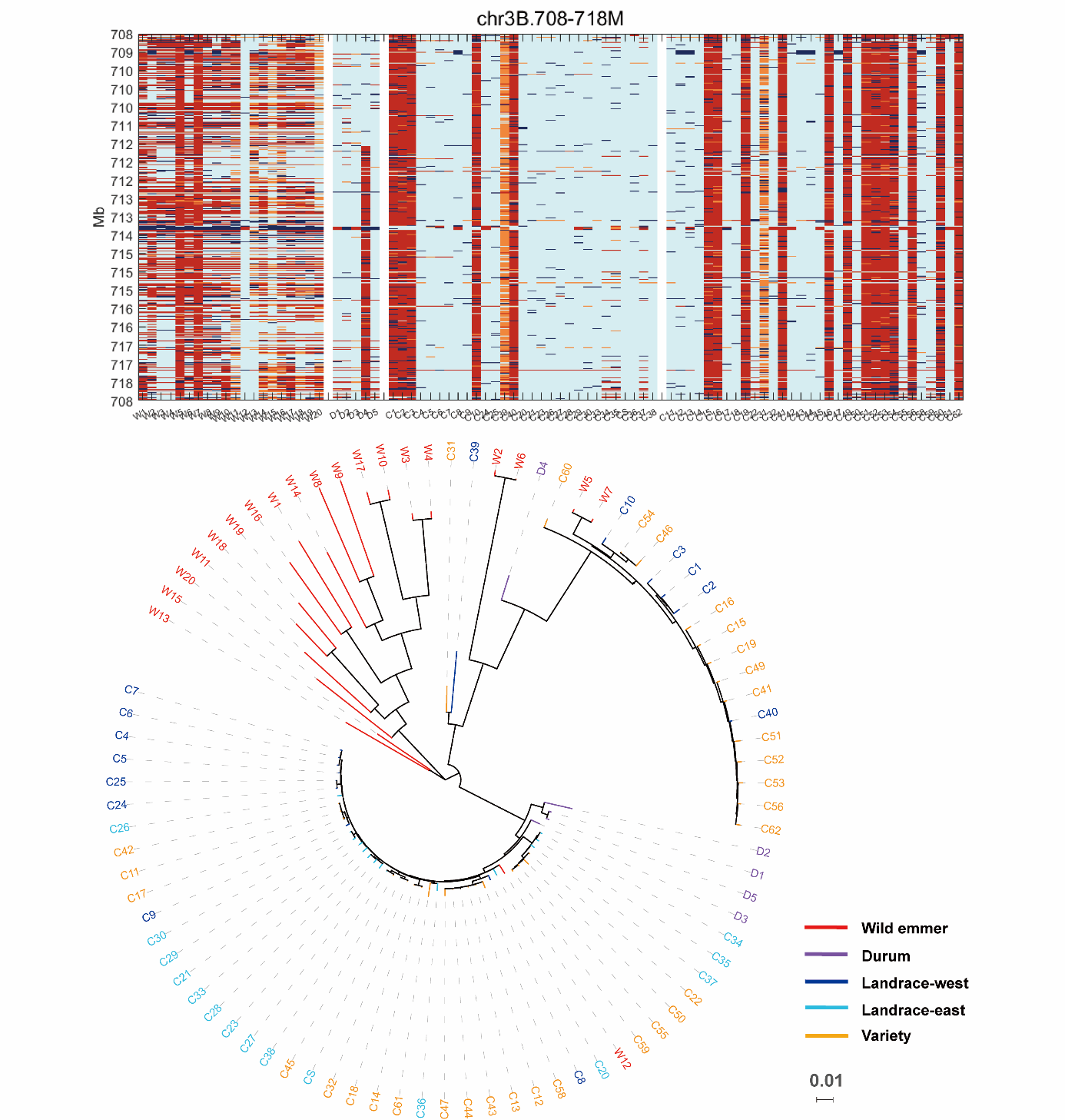


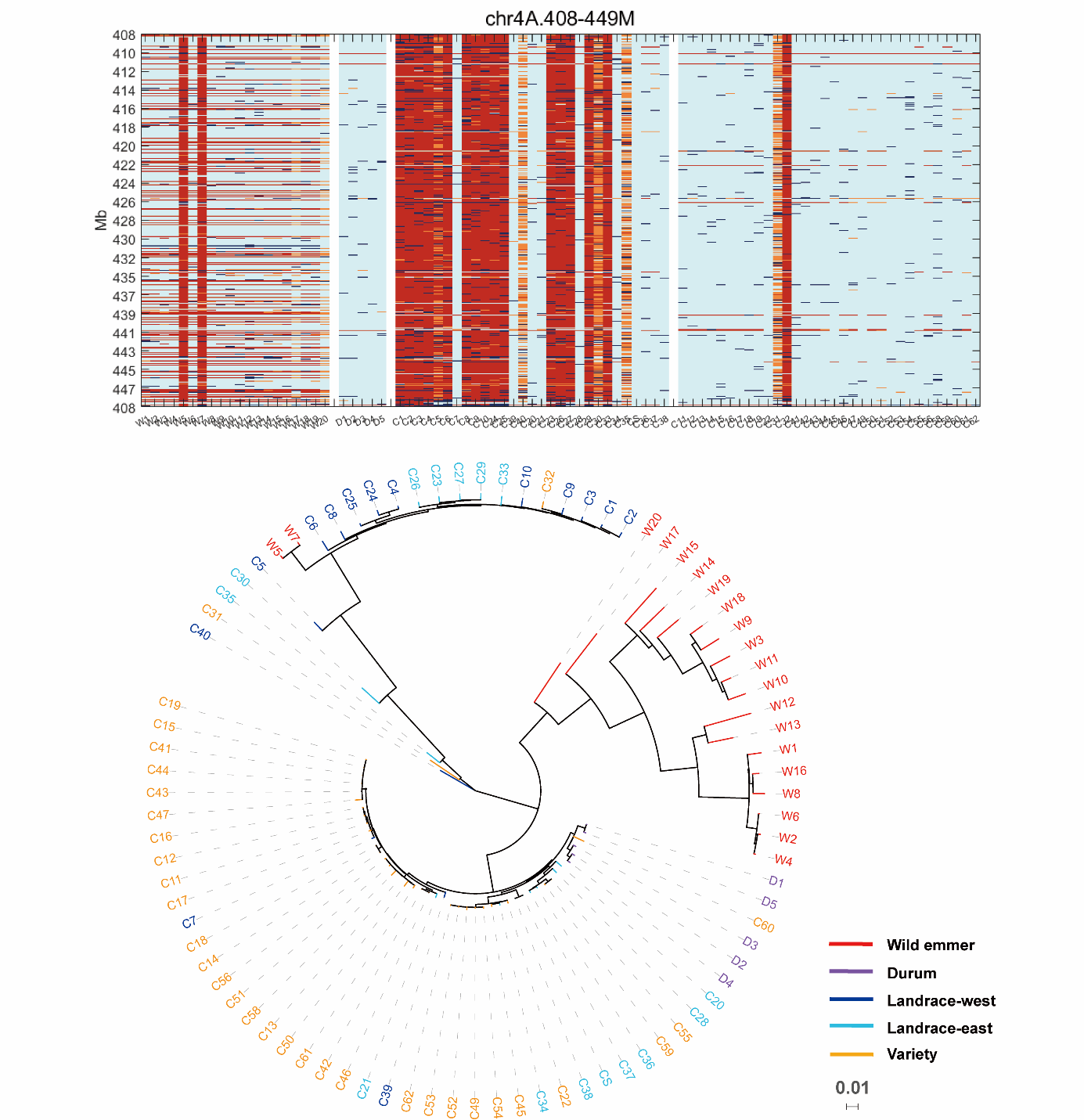


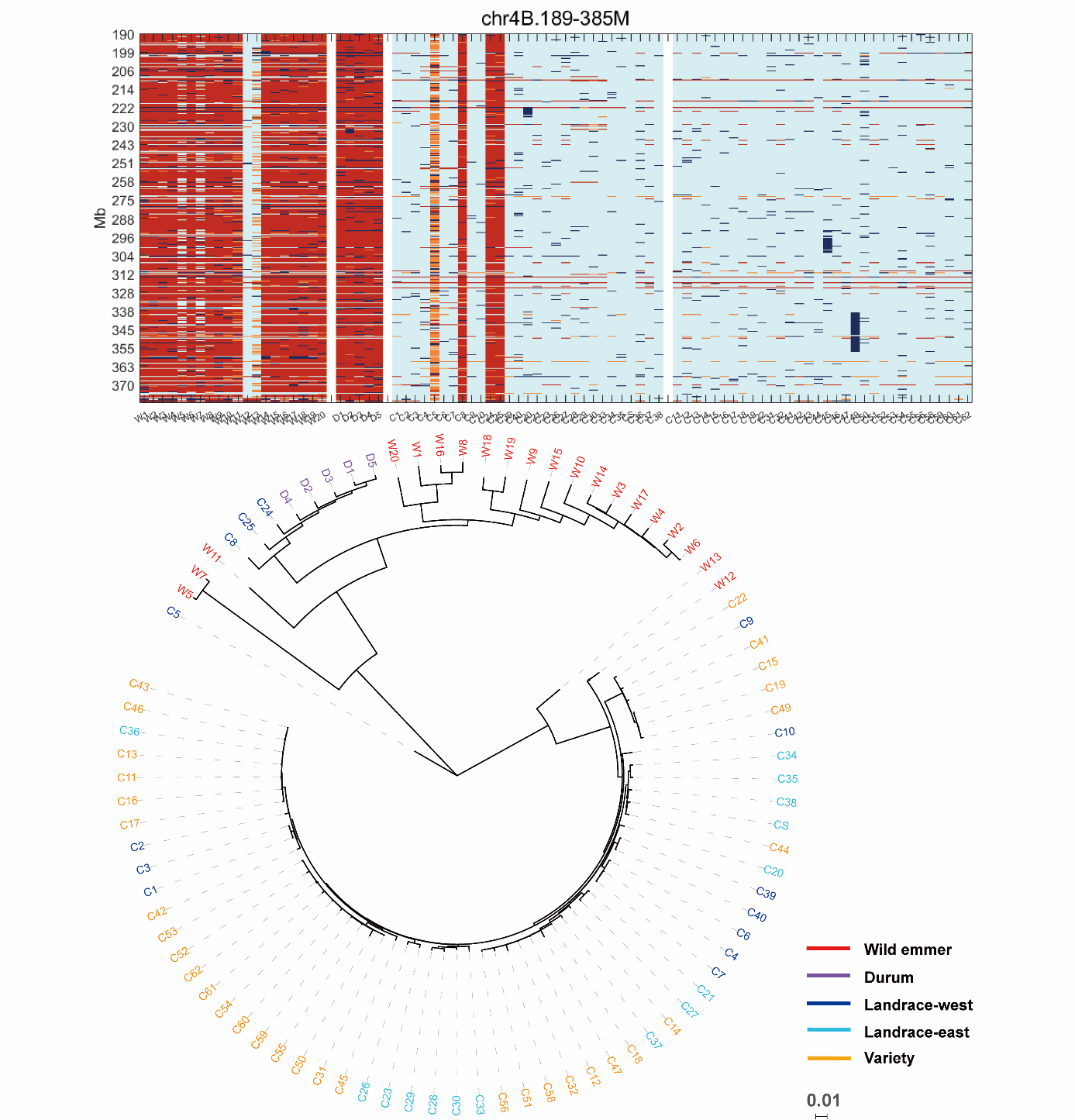


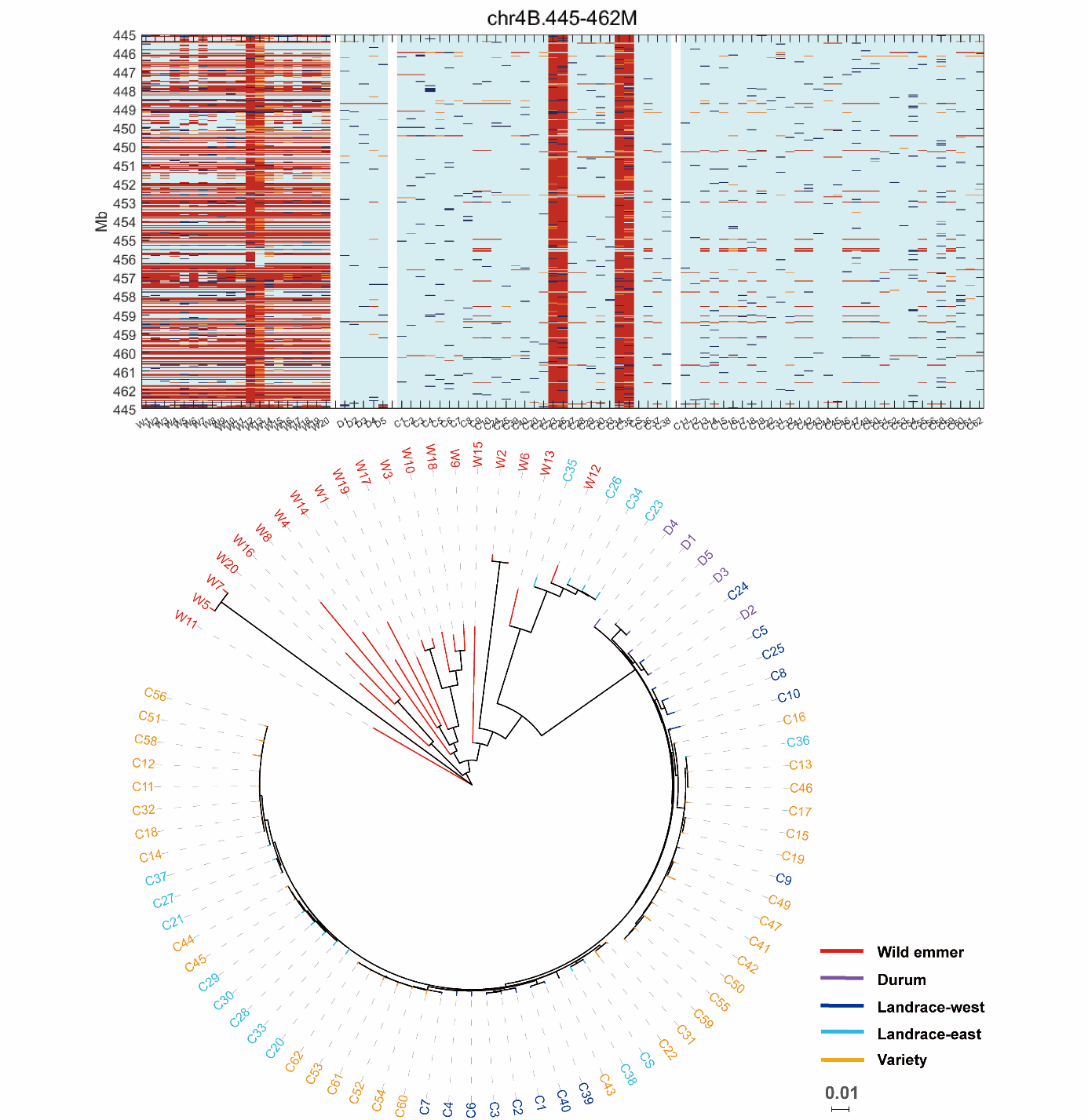


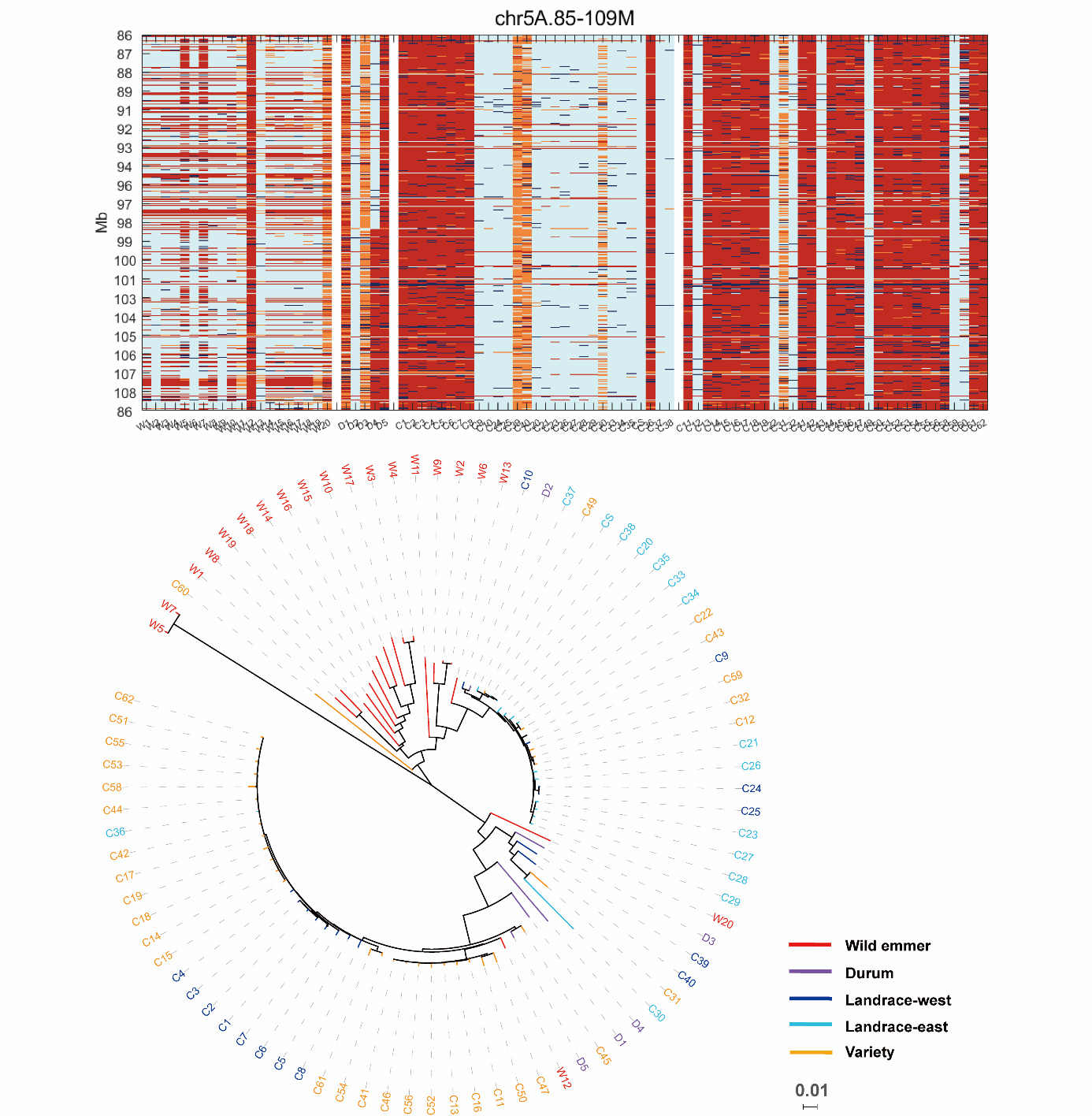


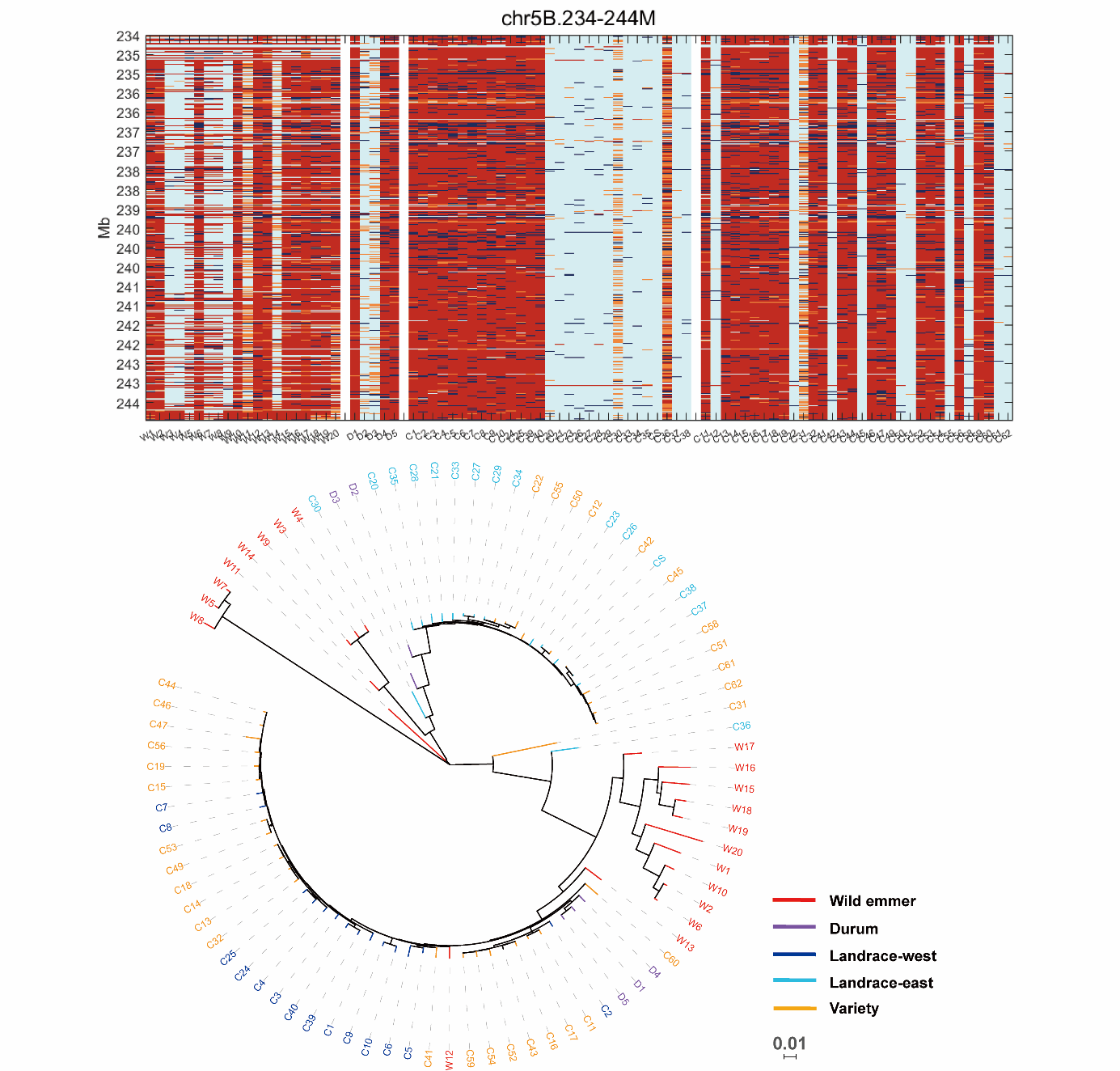


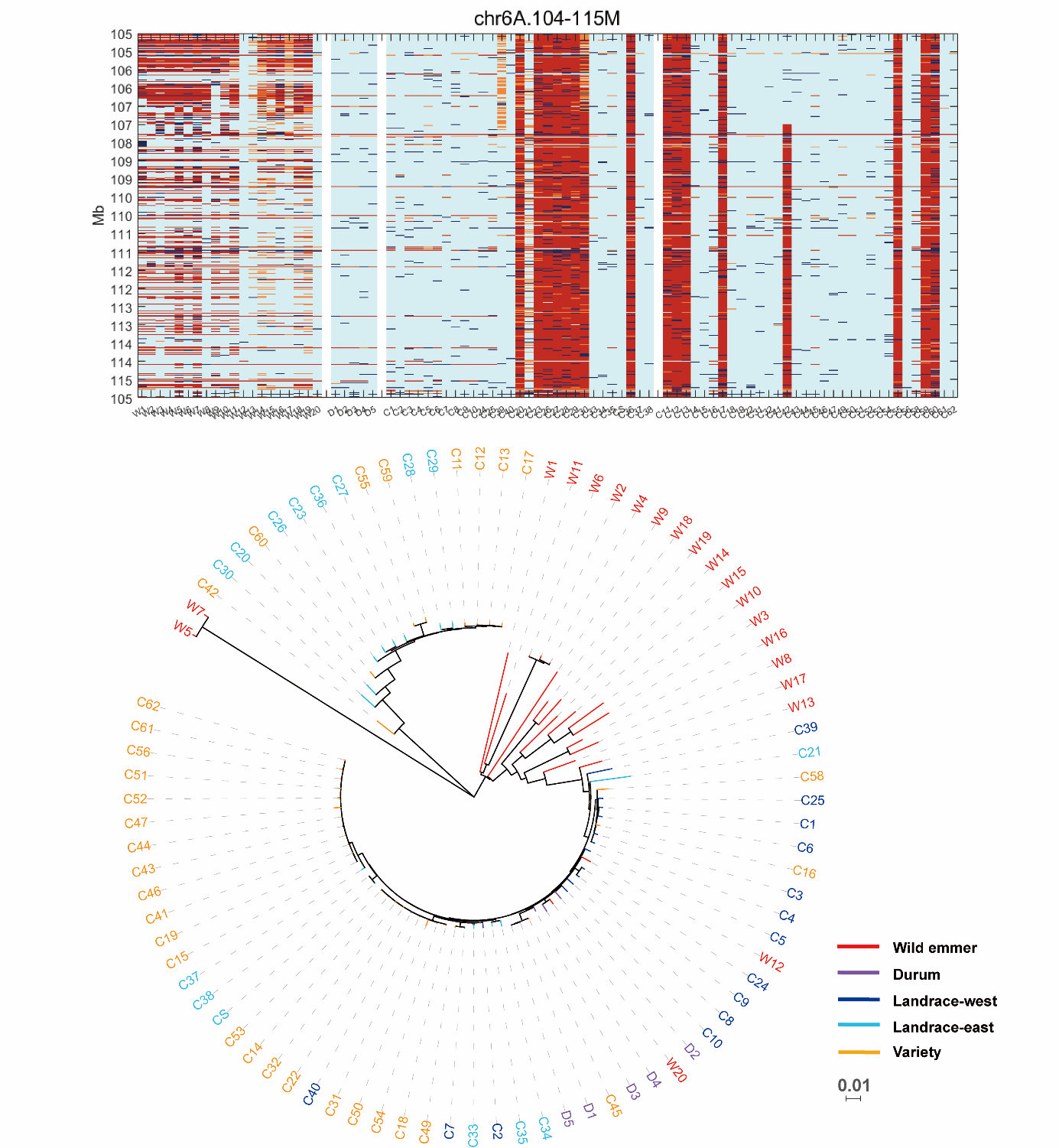


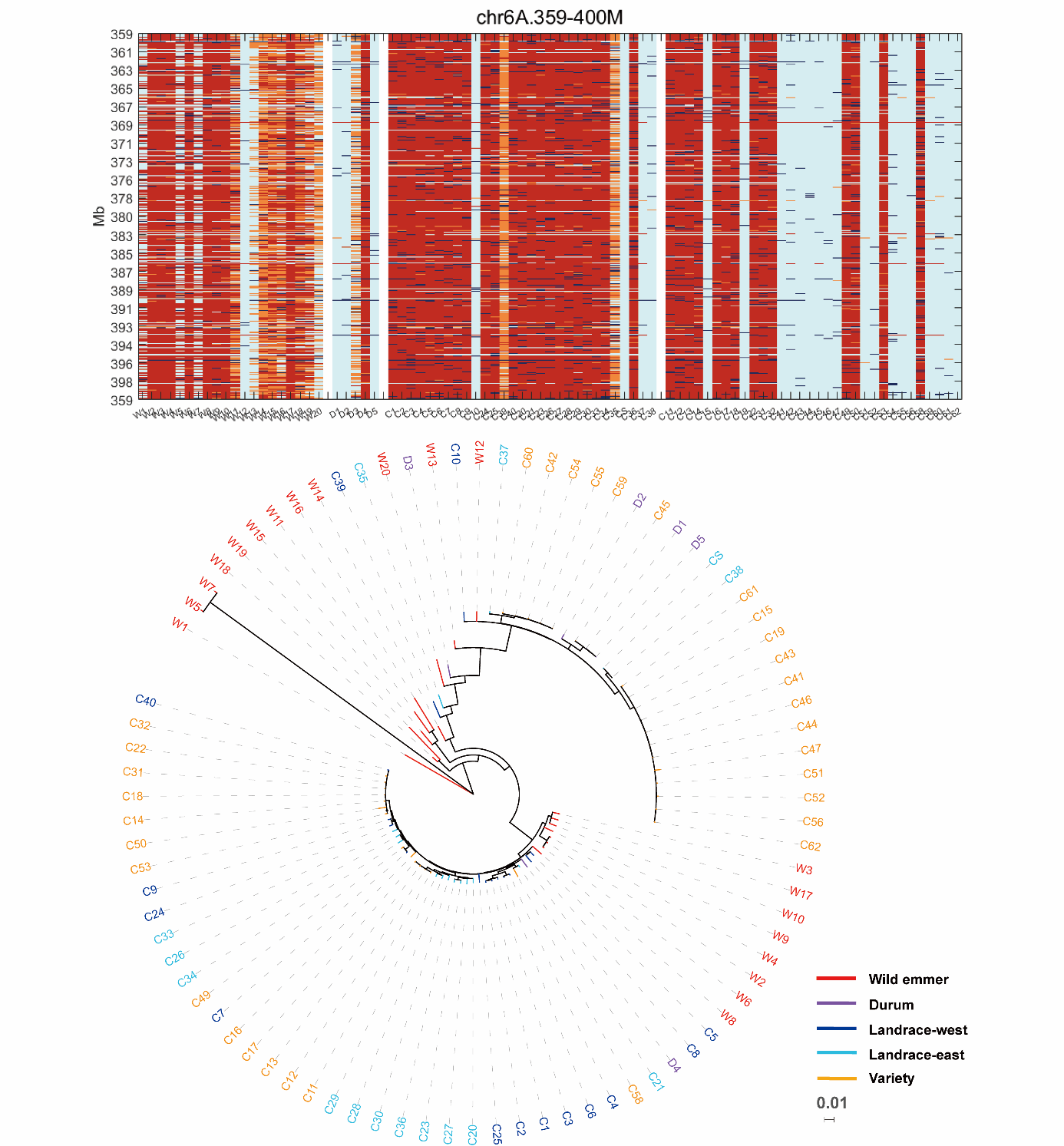


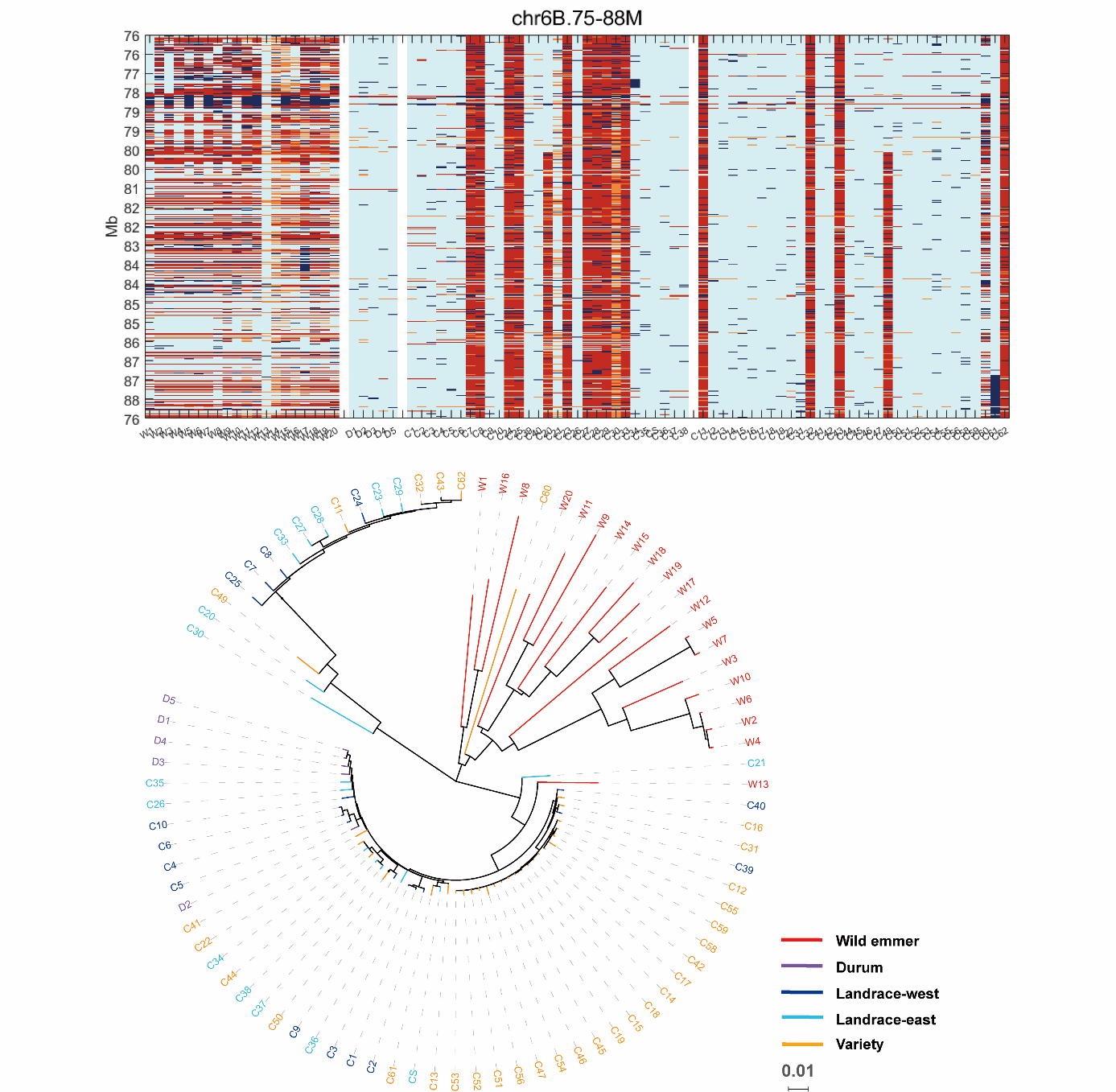


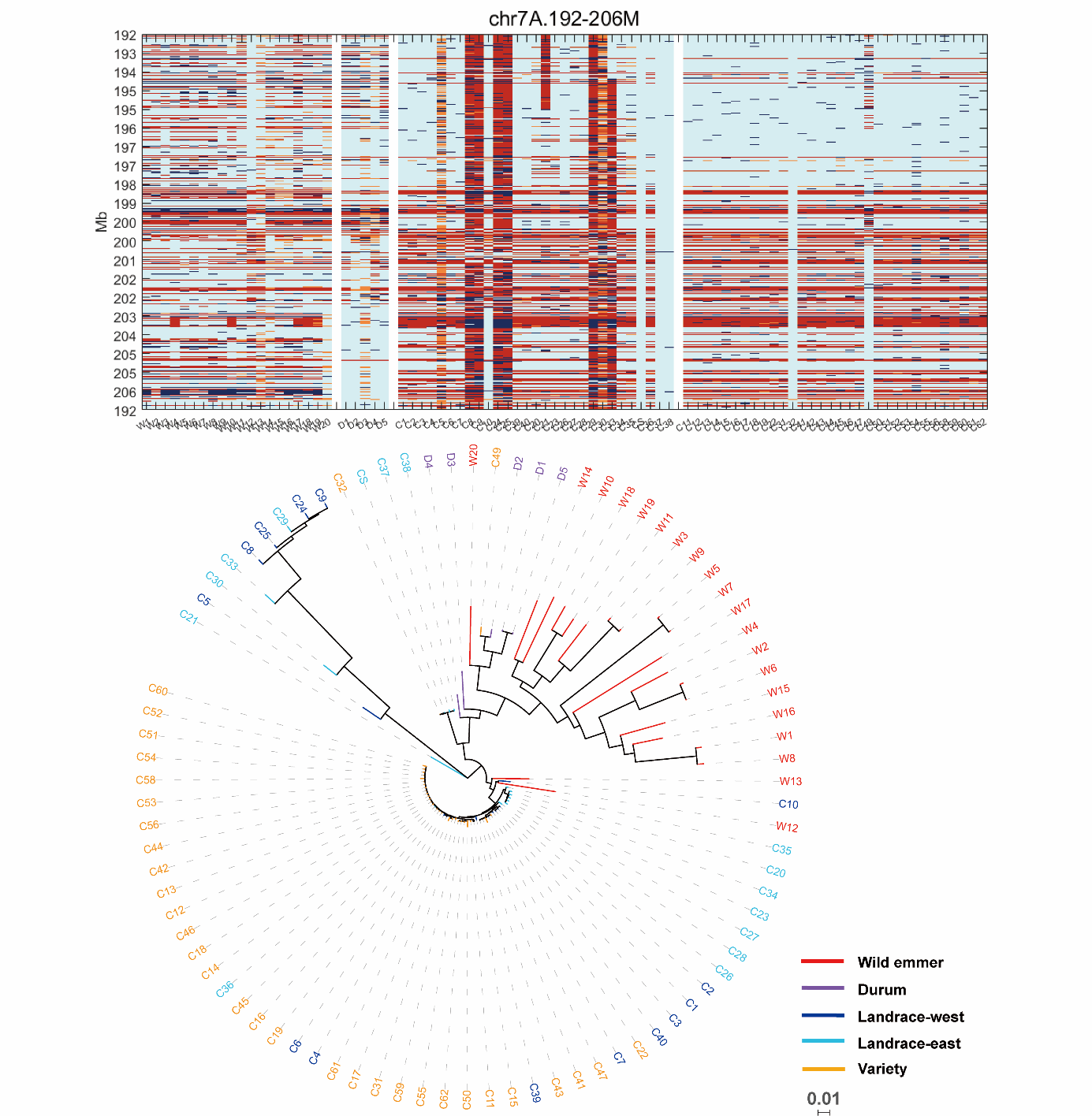


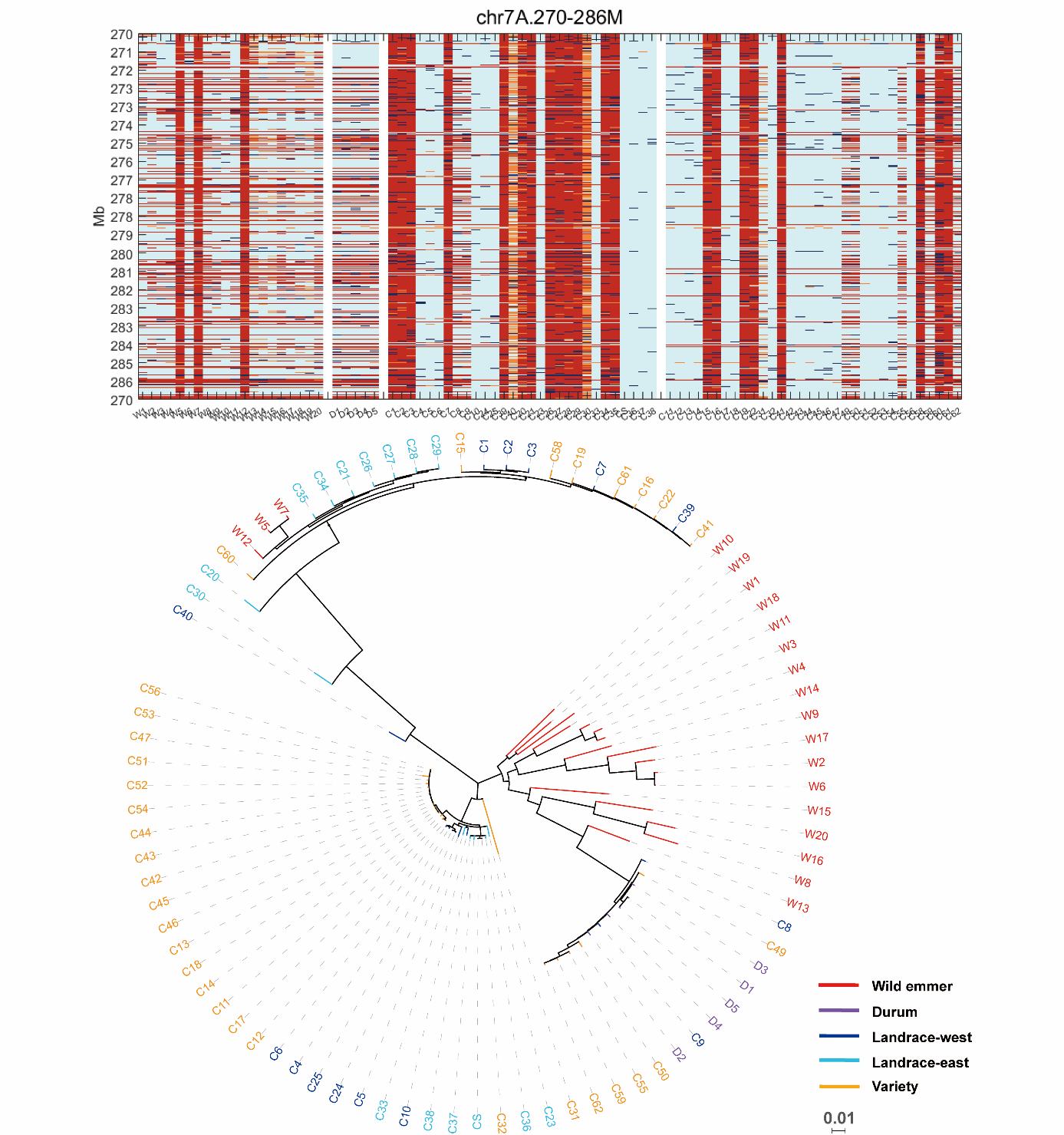


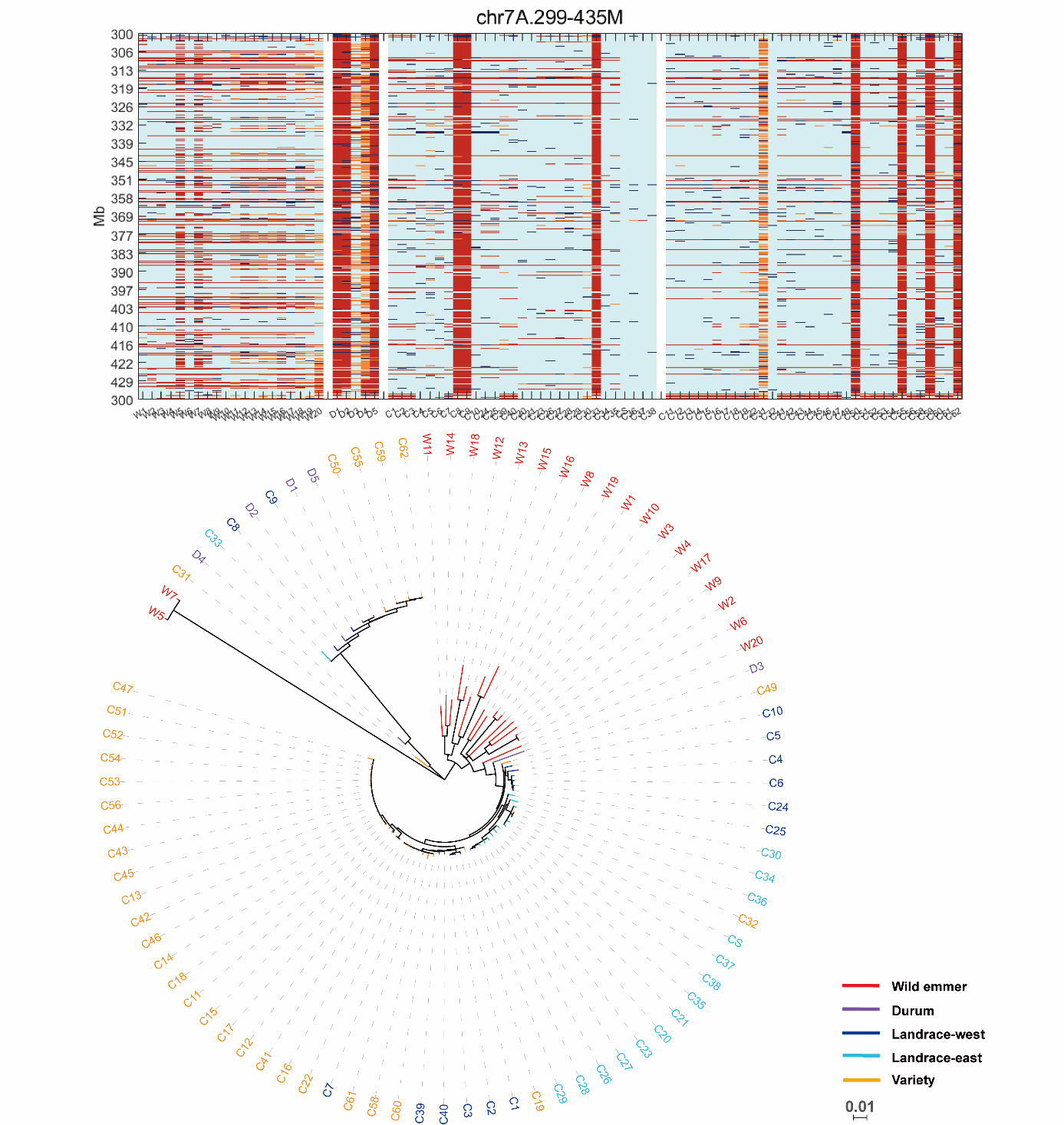


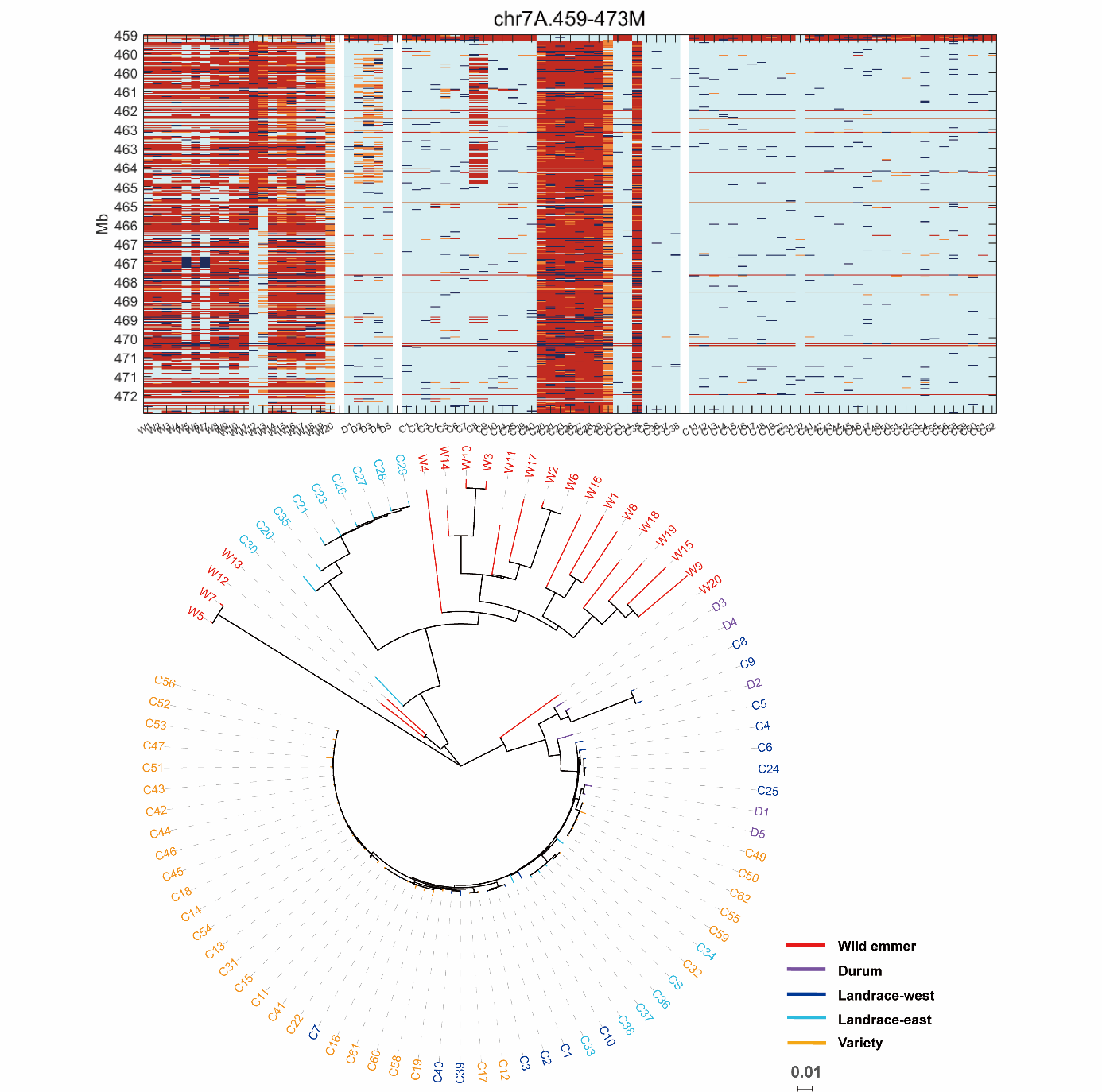


**Figure S16: Haplotype patterns and phylogenetic analysis of regions which length larger than 10Mb.**


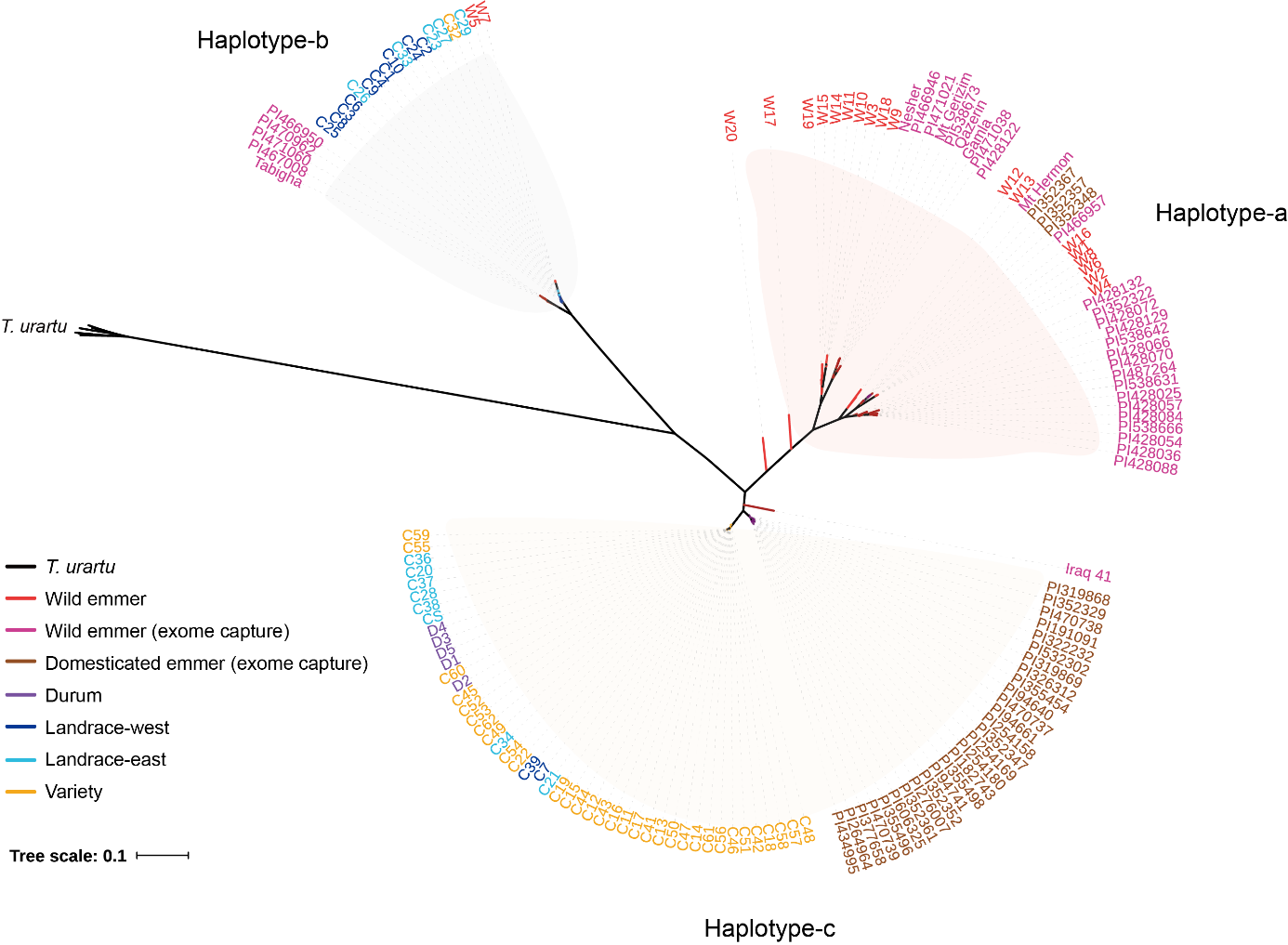


**Figure S17: Neighbour-joining phylogenetic tree of the longest haploblock on chromosome 4A.** The colors of branches indicate different populations. The NJ tree was constructed based on the SNPs in the protein coding region because the SNPs of 9 *T. urartu* accessions were obtained by RNA-seq data. All accessions clustered into three major haplotypes, named haplotype-a, -b, and -c. Wild emmer accessions were found in all three haplotypes. Only one wild emmer (Iraq 41) clustered with most of the domesticated emmer, durum, and cultivated bread wheat accessions. The extreme divergence of the haplotypes in wild emmer likely originated from genetically or geographically different populations of wild emmer.


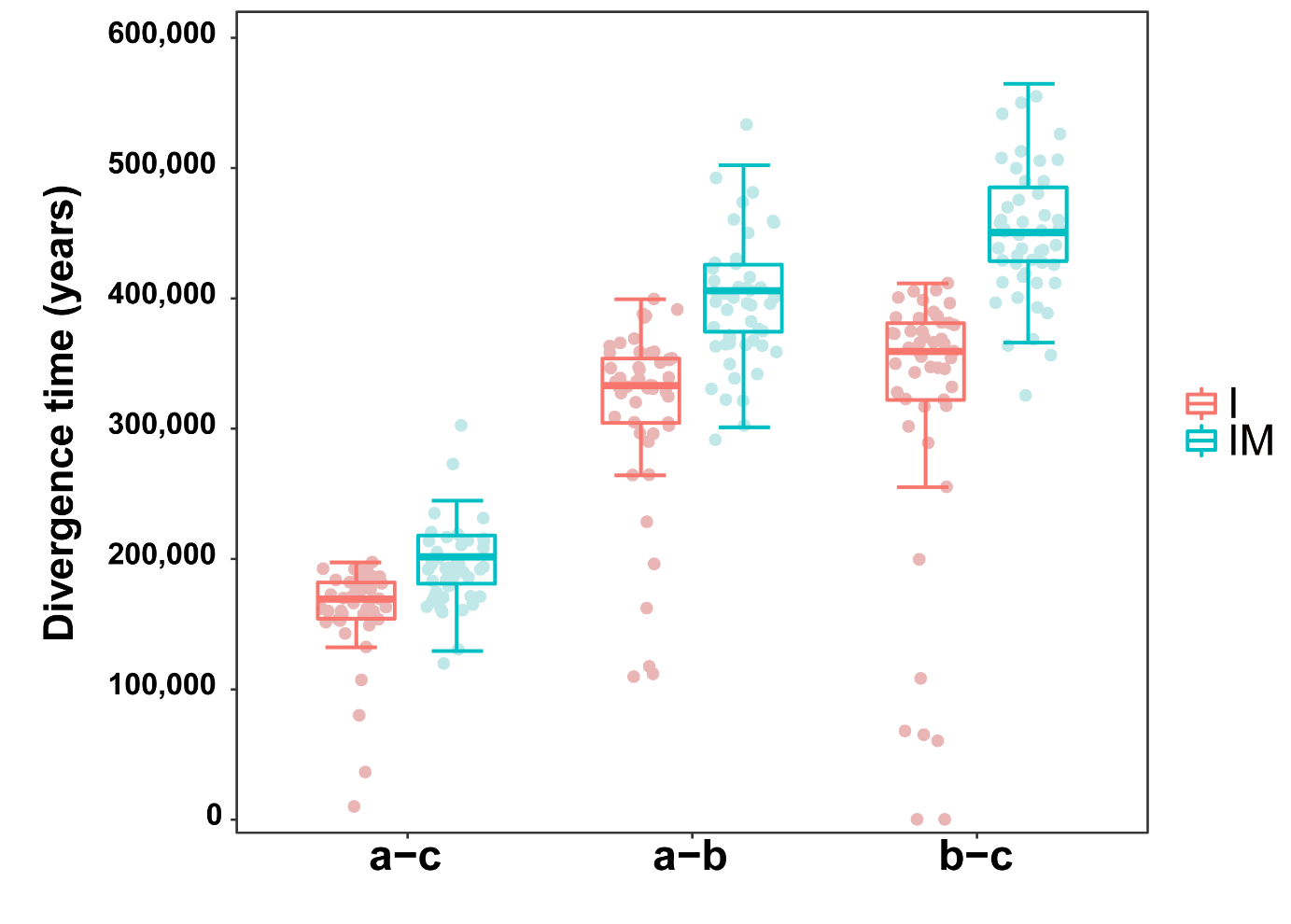


**Figure S18: Divergence time of the different haplotypes of the longest haploblocks on chromosome 4A.** Two models, isolation (I) and isolation-with-migration (IM) were used to estimate the divergence time of three haplotypes.


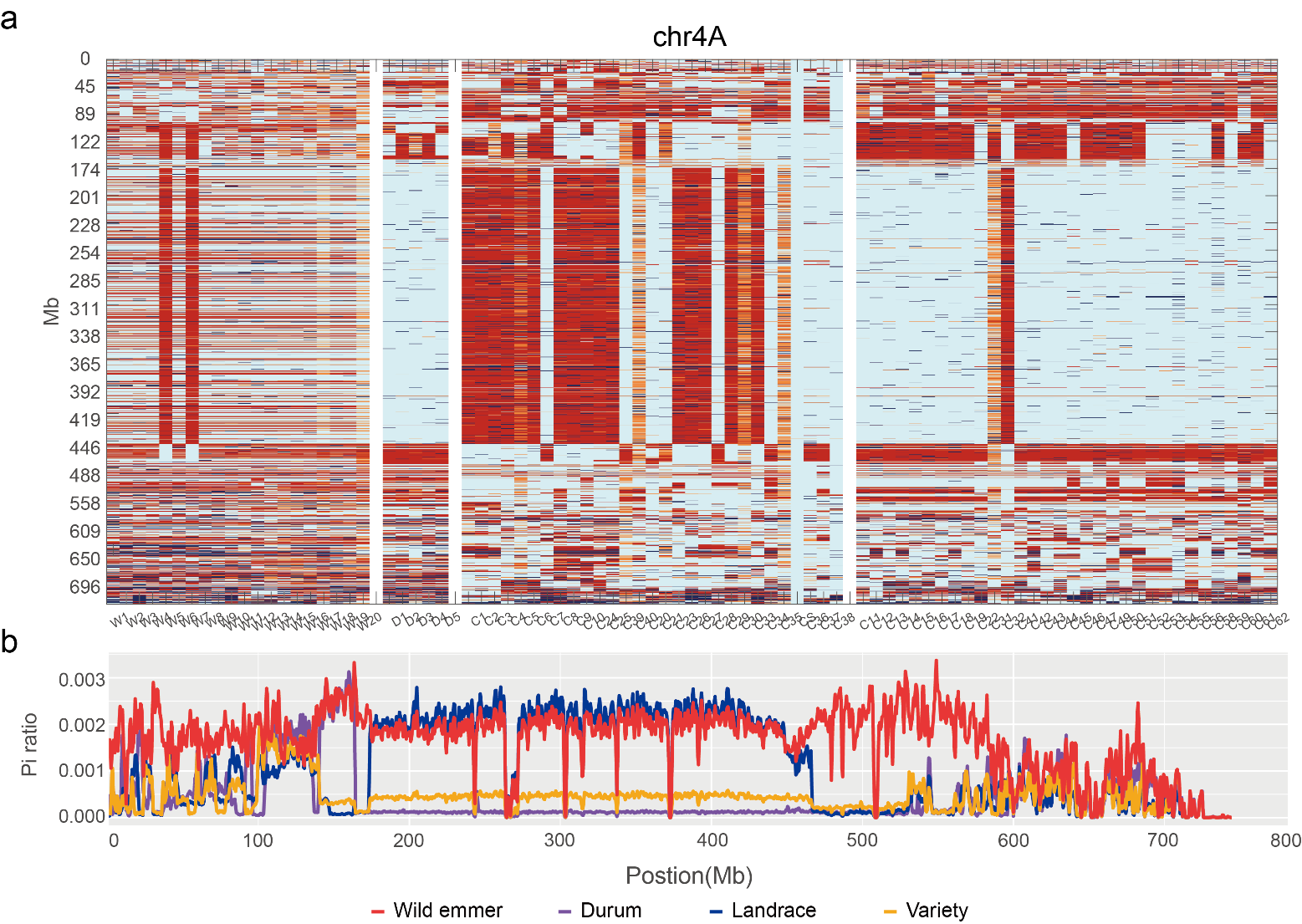
**Figure S19: (a) Haplotype patterns of chromosome 4A in diverse populations.** The haplotypes were constructed for each accession using all the SNPs on chromosome 4A. Different colors donate the genotypes of SNPs, light blue for reference homozygous sites, red for homozygous nonreference sites, orange for heterozygous site, and navy for missing. **(b) The diversity of multiple haplotype in different populations.** Wild emmer and landrace have higher haplotype diversity than Durum and Variety on 170Mb to 440Mb.


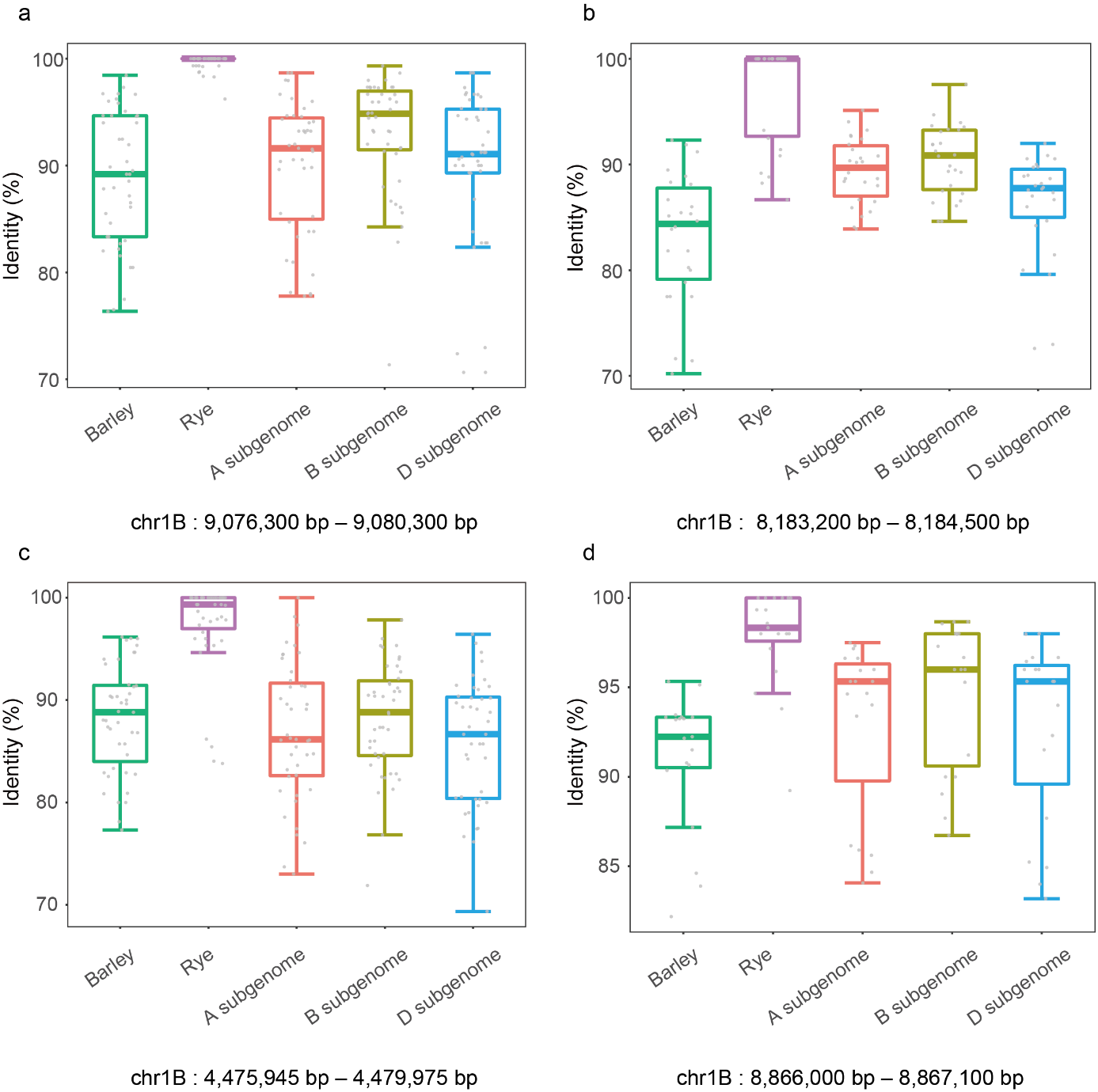


**Figure S20: Boxplots of the sequence identity between reads of C46 mapped to chromosome 1BS and the sequence of outgroups.** The results show that the resequencing reads of C46 that mapped to chromosome 1BS has higher identity with the rye than the other species, such as barley. Four regions (a) 9,076,300 bp to 9,080,300 bp, (b) 8,183,200 bp to 8,184,500 bp, (c) 4,475,945 bp to 4,475,975 bp, and (d) 8,866,000 bp to 8,867,100 bp within 1BS are represented to confirm this result.
