# Supplementary material for: Frequent intra- and inter-species introgression shapes the landscape of genetic variation in bread wheat
Source: Genome Biol. 2019 Jul 12;20:136. doi: 10.1186/s13059-019-1744-x (PMC6624984; doi:10.1186/s13059-019-1744-x)
Supplement: Supplementary file 2 — The maximum likelihood trees of the 1B/1R translocation region on chromosome 1B from 1 to 240 Mb. Figure S22. Absolute sequence divergence dxy value (the number of pairwise differences per site) between each pairwise accession. Figure S23. The pattern of haplotypes sharing in diverse populations on chromosome 1B. Figure S24. The mapping statistics of the resequencing data in introgressed regions visualized by Integrative Genomics Viewer (IGV). Figure S25. The length of the introgressions of each bread wheat accession. Figure S26. The length of the introgressions of A, B, and D subgenomes. Figure S27. The introgressied segments with accurate breakpoints identified in Chinese Spring. Figure S28. Single nucleotide polymorphism (SNP) density across chromosome 2D of the Chinese Spring. Figure S29. The maximum likelihood tree of a 33 Mb introgression fragment on chromosome 5B from ~ 497 Mb to 529 Mb (853000 SNP). Figure S30. The maximum likelihood tree of an introgression fragment from Ae.tauschii. Figure S31. The maximum likelihood tree of a introgression segment on chromosome 5D form 555, 981, 000 bp to 556, 438, 500 bp (70000 SNP). Figure S32. Absolute sequence divergence dxy value (the number of pairwise differences per site) between each pairwise accessions on chromosome 4D from 500, 862, 001 bp to 501, 999, 000 bp. Figure S33. Absolute sequence divergence dxy value (the number of pairwise differences per site) between each pairwise accessions on chromosome 5D form 555, 981, 000 bp to 556, 438, 500 bp. Figure S34. Comparison of mapping statistics and gene expression of TraesCS2B01G113800 in intro (introgression) and non-intro (non-introgression) accessions. Figure S35. Comparison of mapping statistics and gene expression of TraesCS2B01G534200 in intro (introgression) and non-intro (non-introgression) accessions. Figure S36. The frequency of haplotype-c. Figure S37. Relative frequency difference (RFD) of CNVs between landraces and varieties. Table S1. Detailed informa [file 13059_2019_1744_MOESM2_ESM.docx]

Supplementary Materials for

**Frequent intra- and inter-species introgression shape the landscape of genetic variation in bread wheat**

This file includes:

Figures S21 to S37

Tables S1 to S3

Tables S10 to S11

Table S14

Tables S18 to S19

**
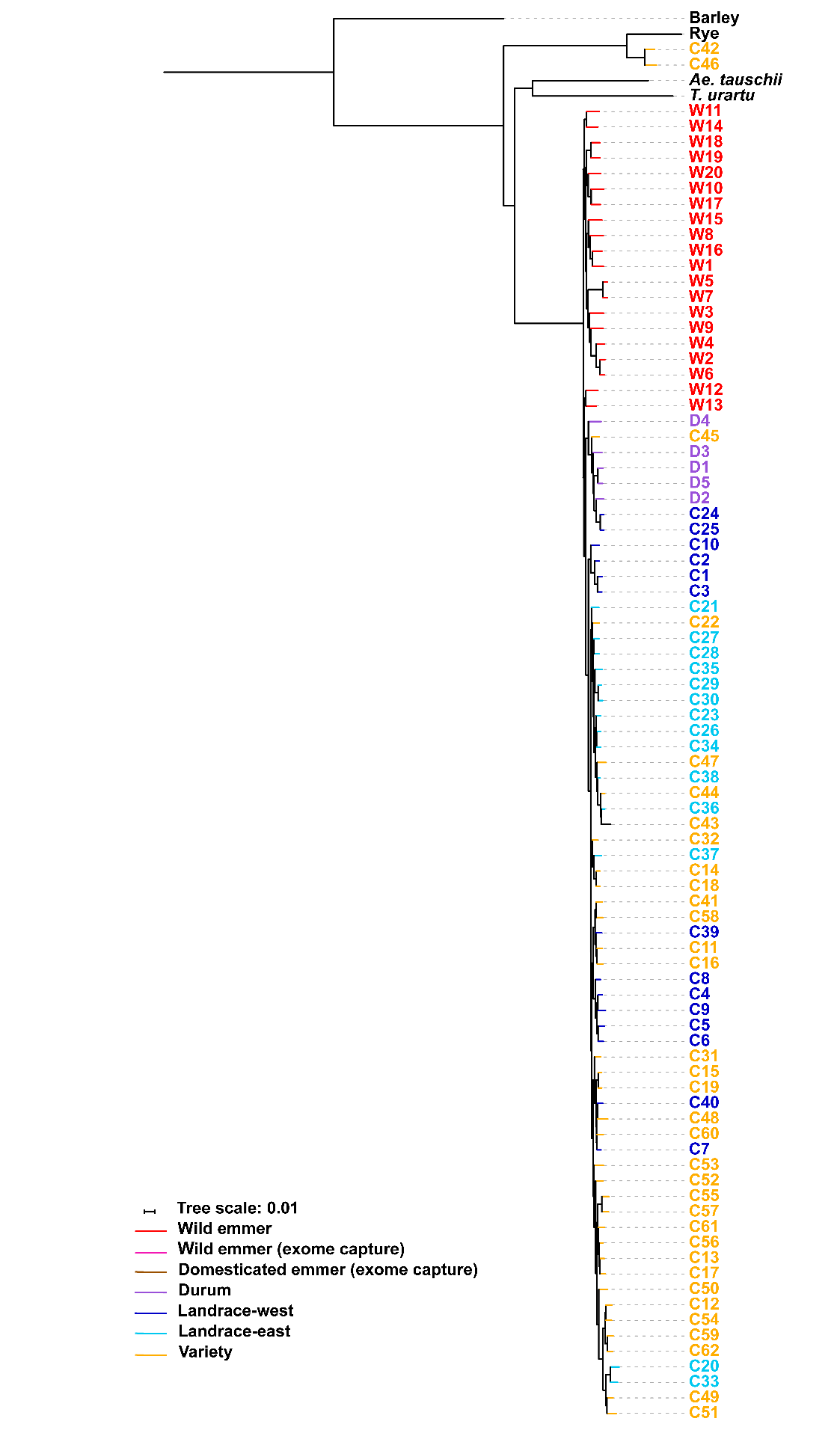
**

**Figure S21: The maximum likelihood trees of the 1B/1R translocation region on chromosome 1B from 1 to 240 Mb.** Two Chinese variety lines, C42 and C46 clustered with rye together, which confirmed the 1B/1R translocation in these two accessions.


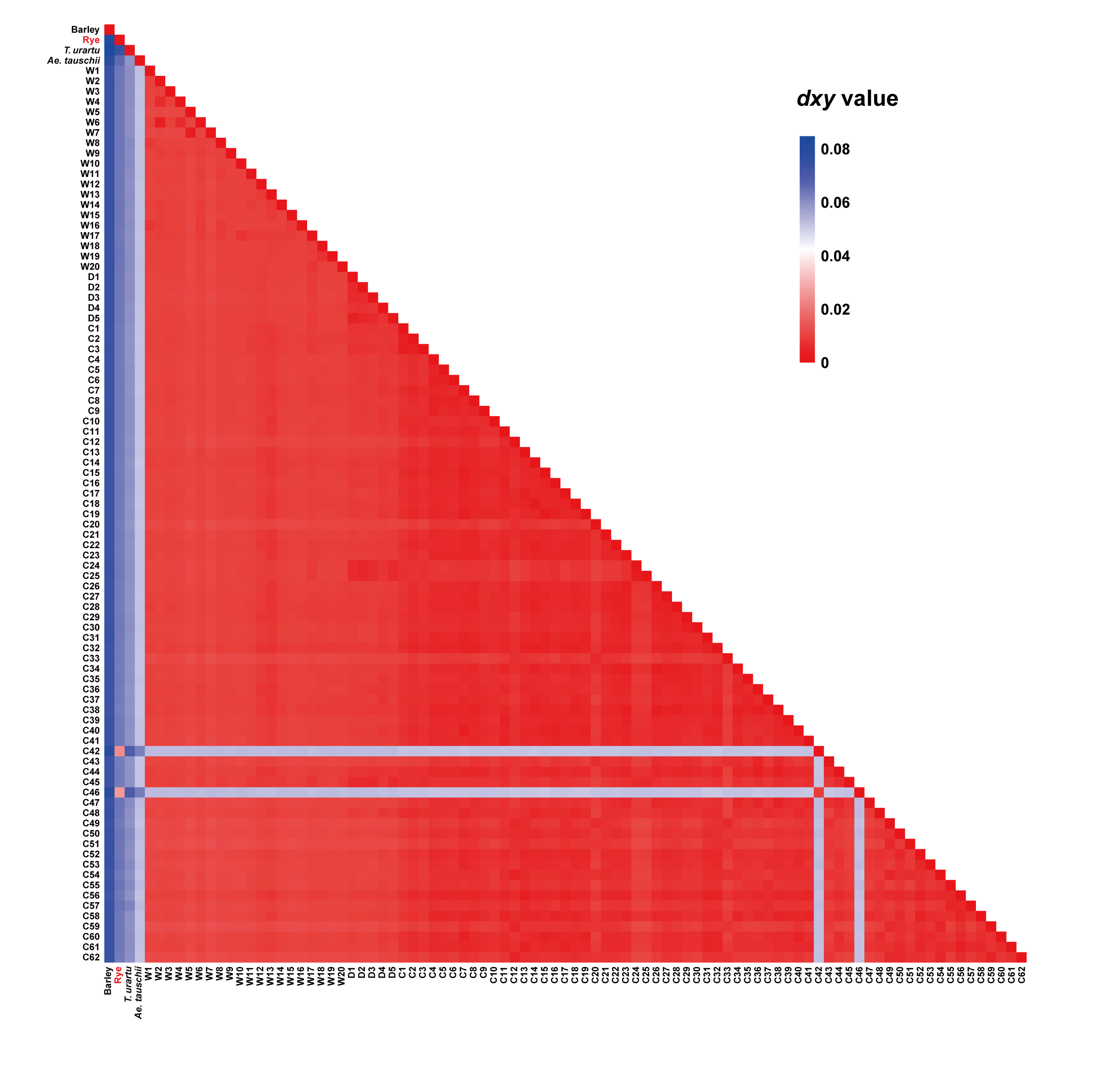


**Figure S22: Absolute sequence divergence** ***dxy* value (the number of pairwise differences per site) between each pairwise accession.** *Dxy* values are highlighted with different density colours. Two Chinese variety accessions (C42 and C46) have a lower *dxy* with rye, which validated the introgression from rye into the C42 and C46 accessions.

**
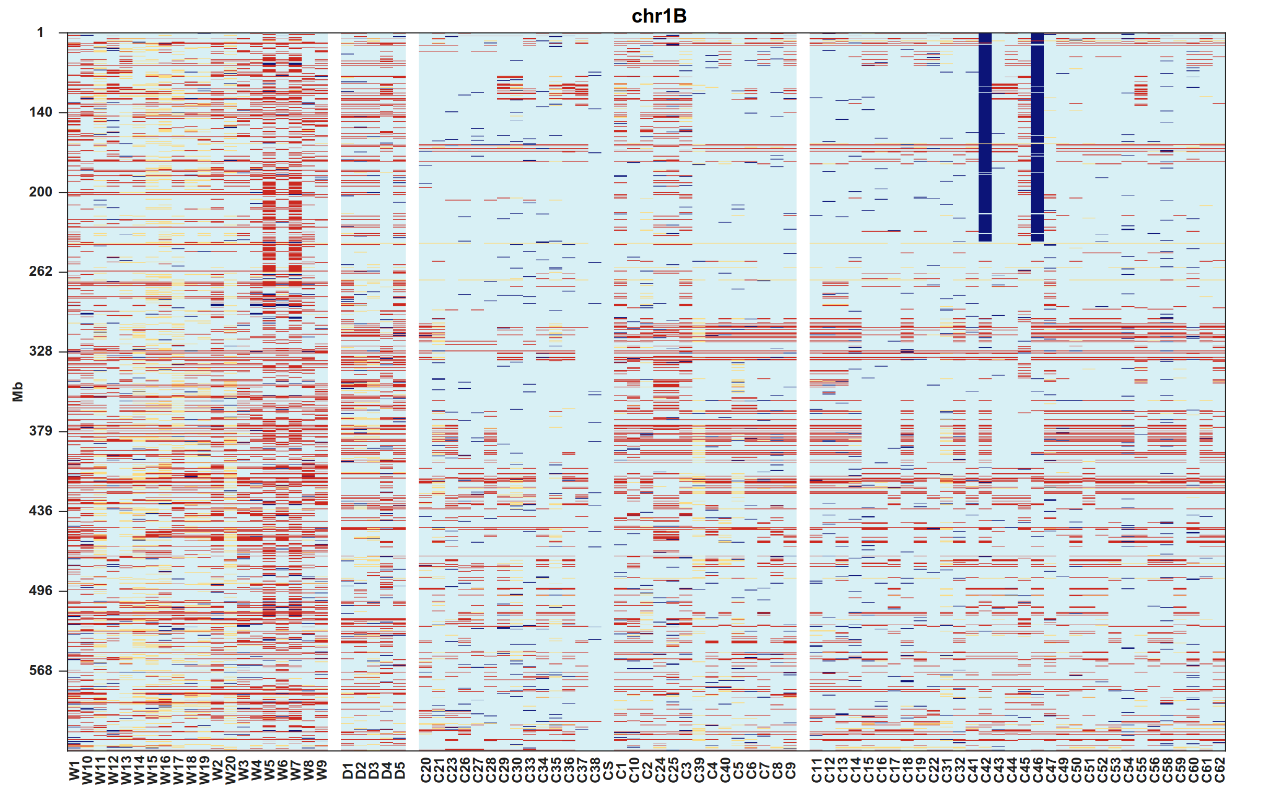
**

**Figure S23: The pattern of haplotypes sharing in diverse populations on chromosome 1B.** The haplotypes were constructed using all the SNPs (MAF > 0.05) in corresponding chromosomes. Lines and columns represent SNP sites and accessions, respectively. Alleles that are identical to or different from those in the IWGSC RefSeq v1.0 are indicated by 0 and 1, respectively. Different colours represent the genotype information (0/0, light blue; 0/1, yellow; 1/1, red; missing, dark blue). The continuous missing SNPs of a ~250-Mb region (0-250 Mb) in C42 and C46 on chromosome 1B were identical to the actual position of 1RS translocation from rye to bread wheat.


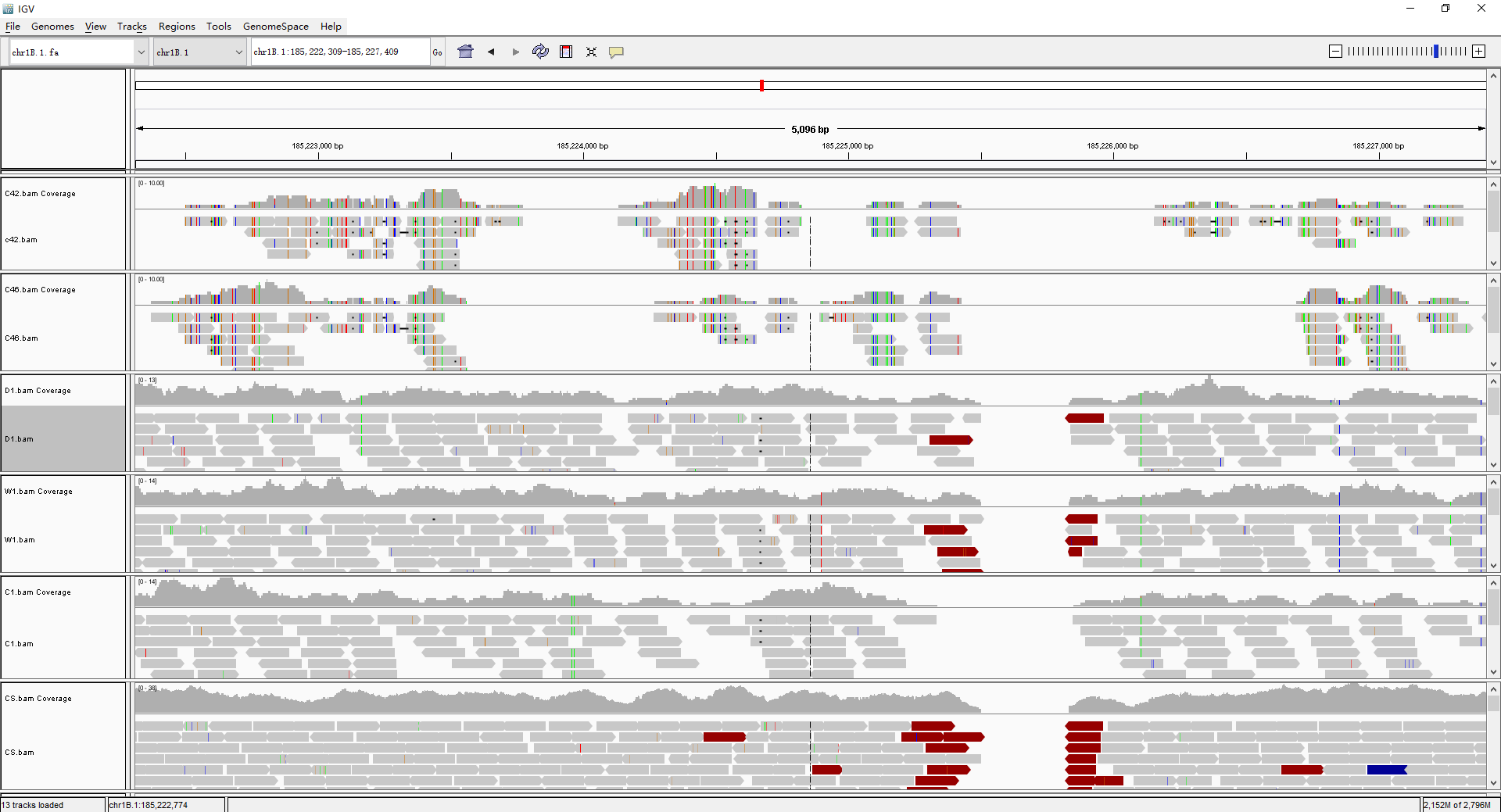

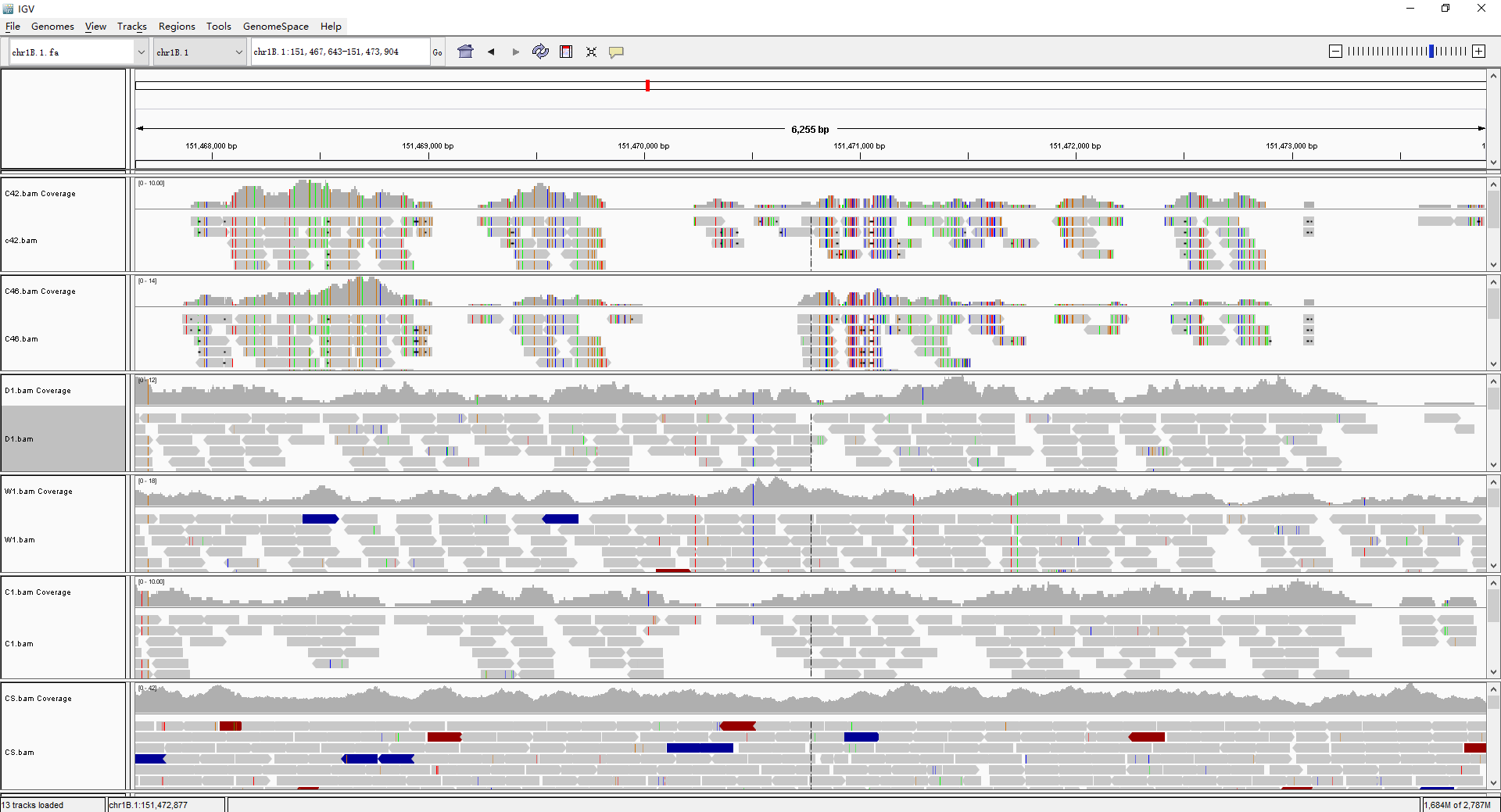


**Figure S24: The** **mapping statistics of the resequencing data in introgressed regions visualized by Integrative Genomics Viewer (IGV).** The IGV shows that the mapped reads of two Chinese varieties (C42 and C46) with 1RS translocation from rye resulted in excessive mismatched sites in the compared with the four other accessions without this translocation. The location of the two regions are chromosome 1B: 151,467,643 to 151,473,904 bp and chromosome 1B: 185,222,309 to 185,227,409 bp.


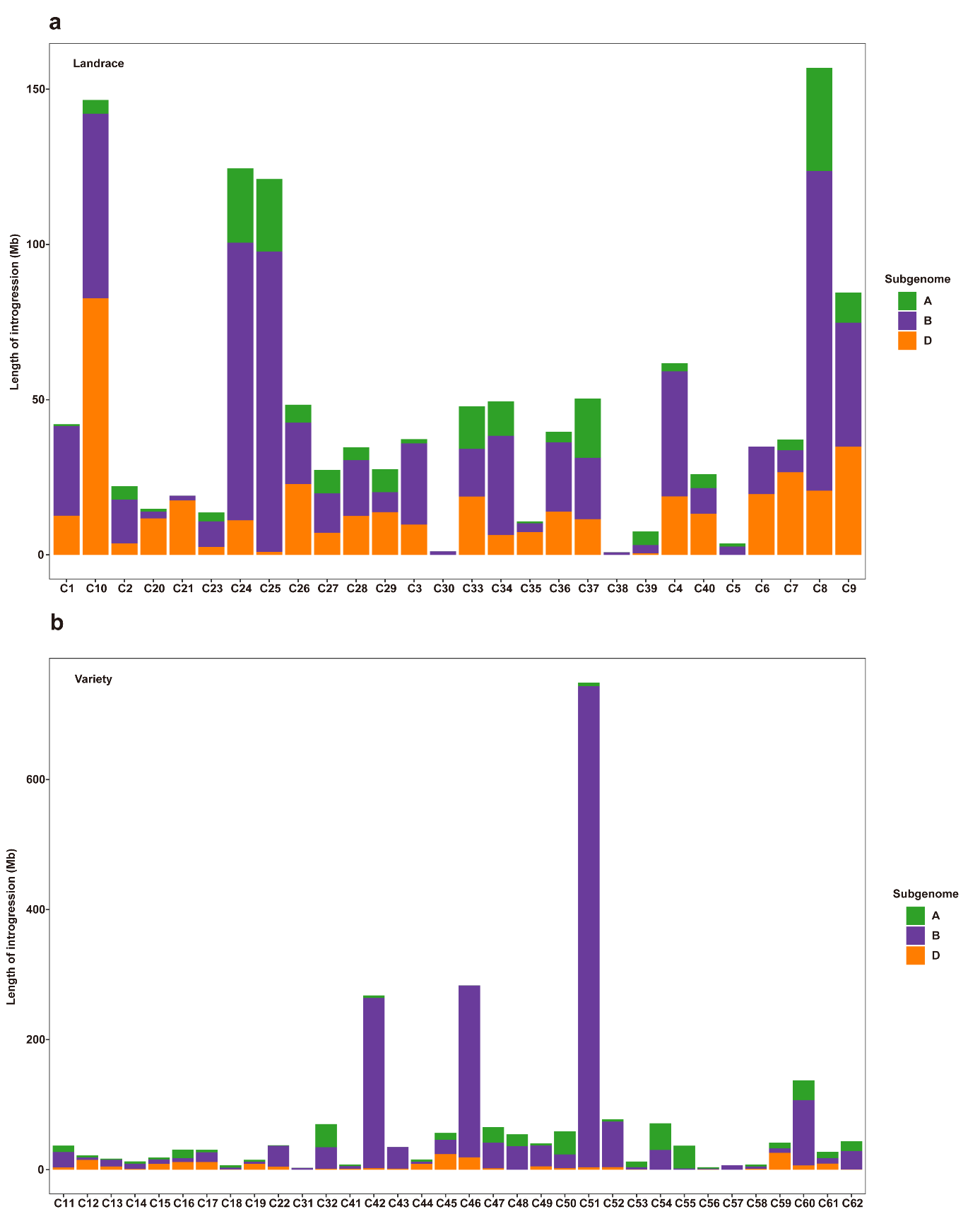


**Figure S25:** **The length of the introgressions of each bread wheat accession.** (a) and (b) separately display the lengths of introgressed regions in the landraces and varieties.


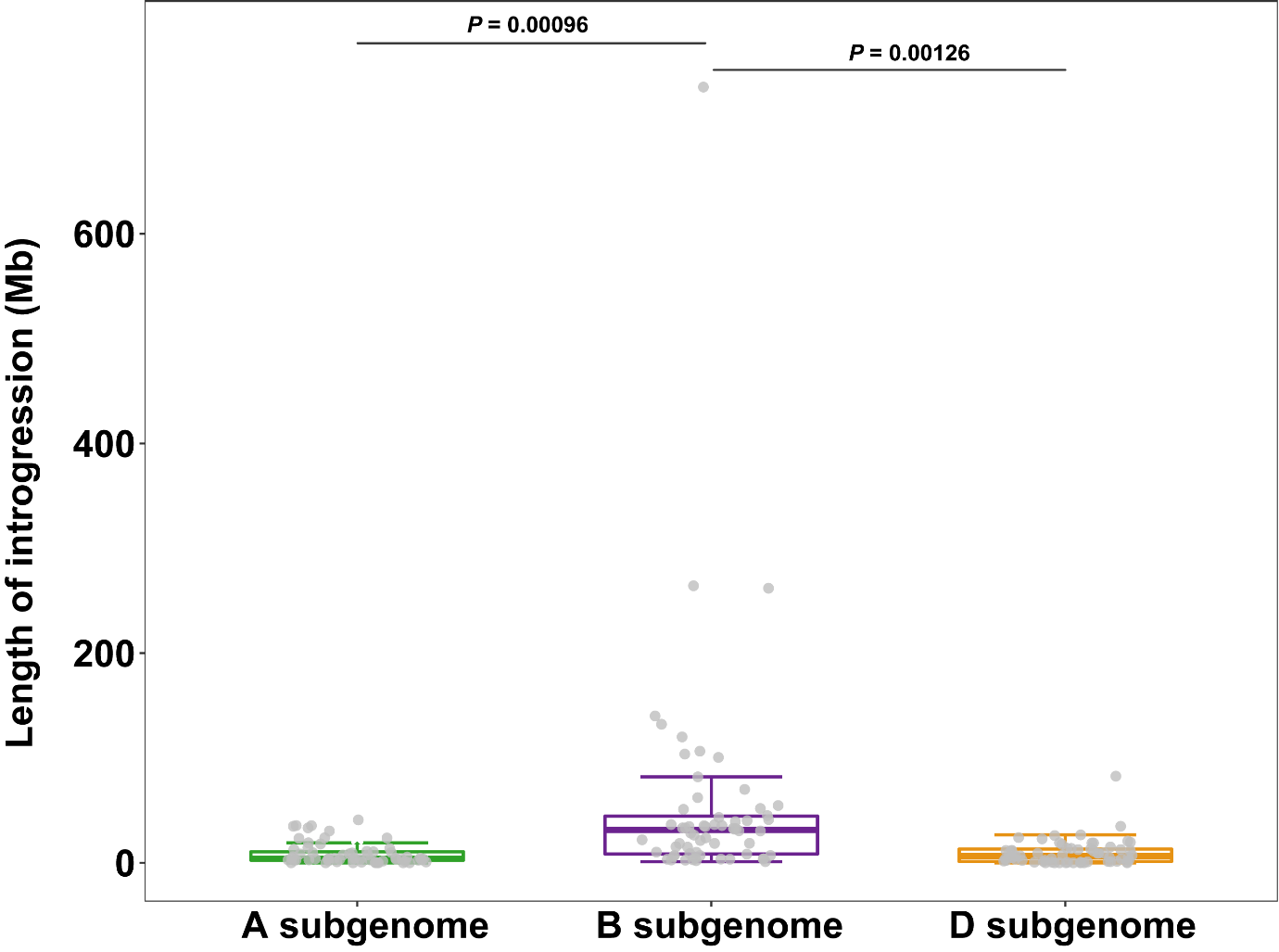


**Figure S26: The length of the introgressions of A, B, and D subgenomes.** The total length of the segments inferred to be introgressive per accession on the B subgenome was greater than those of the A and D subgenomes


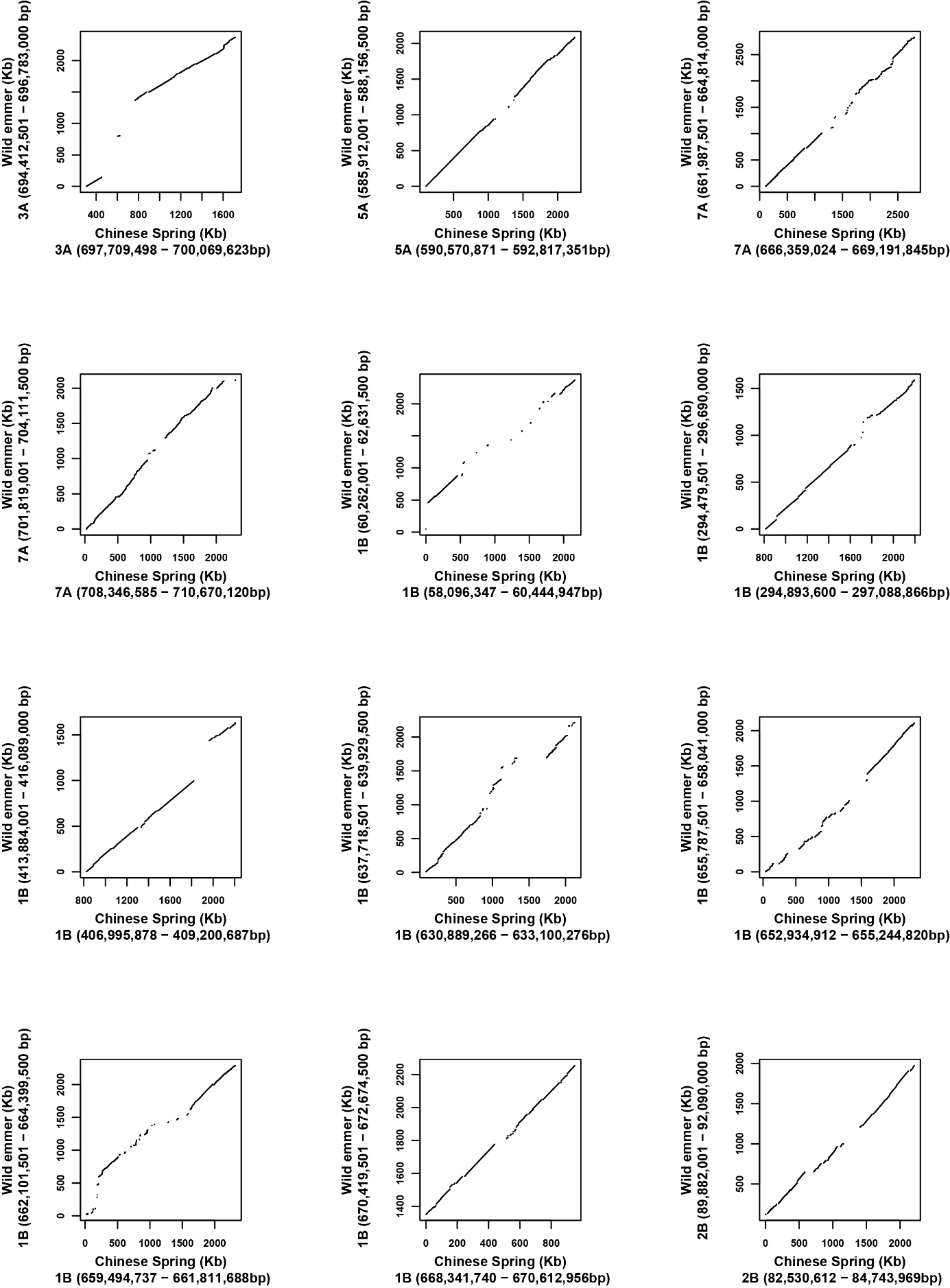


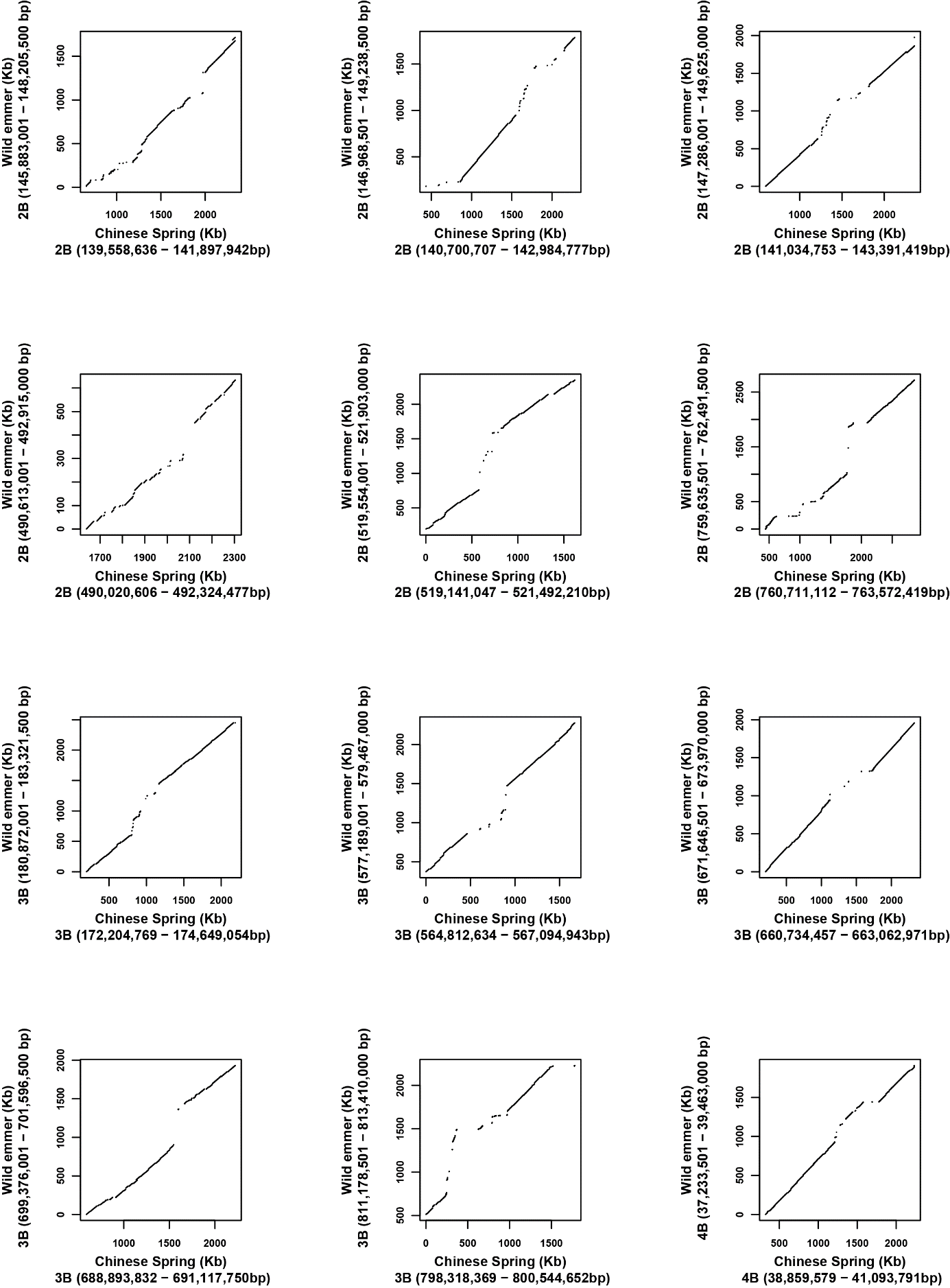


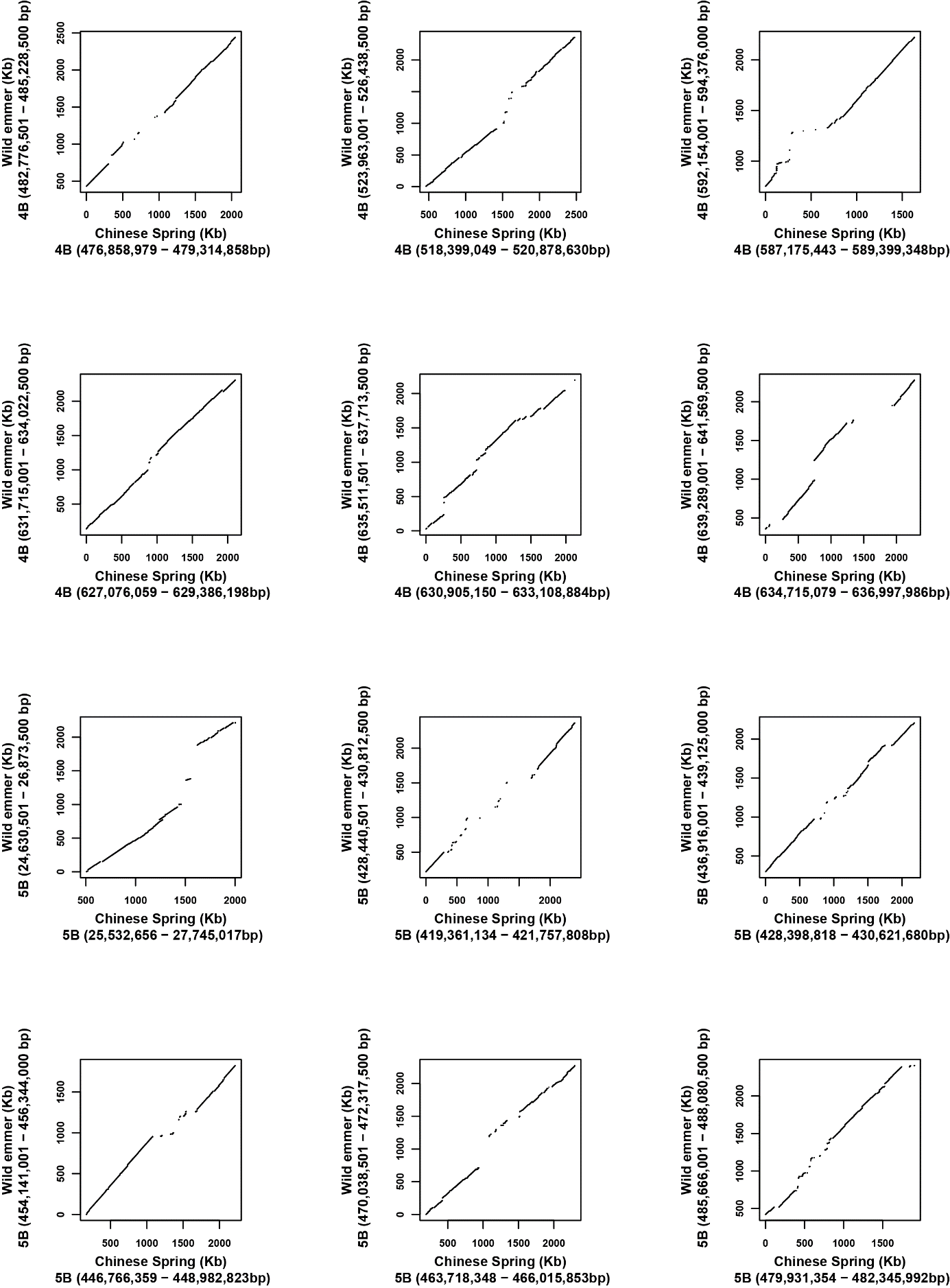


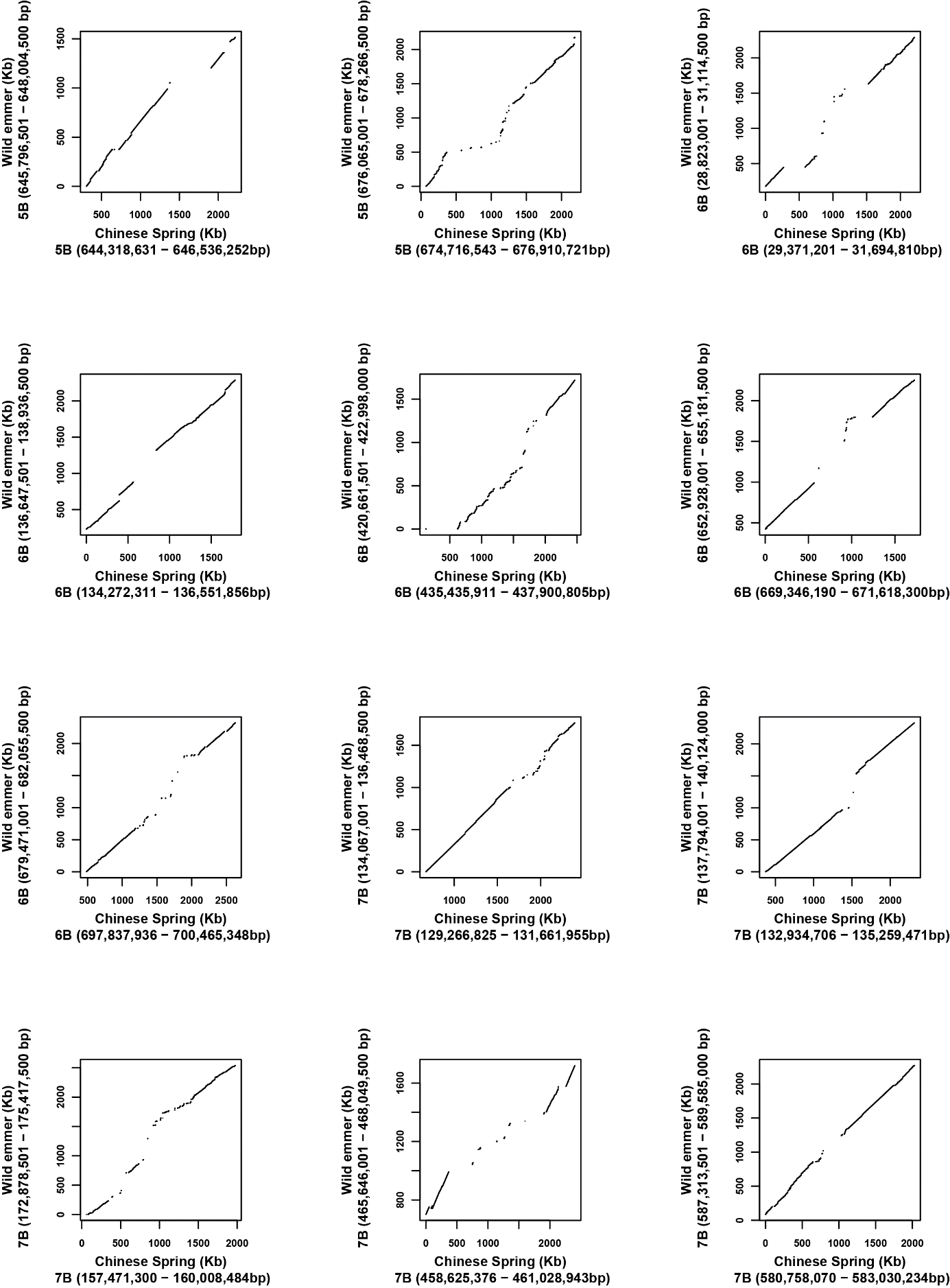


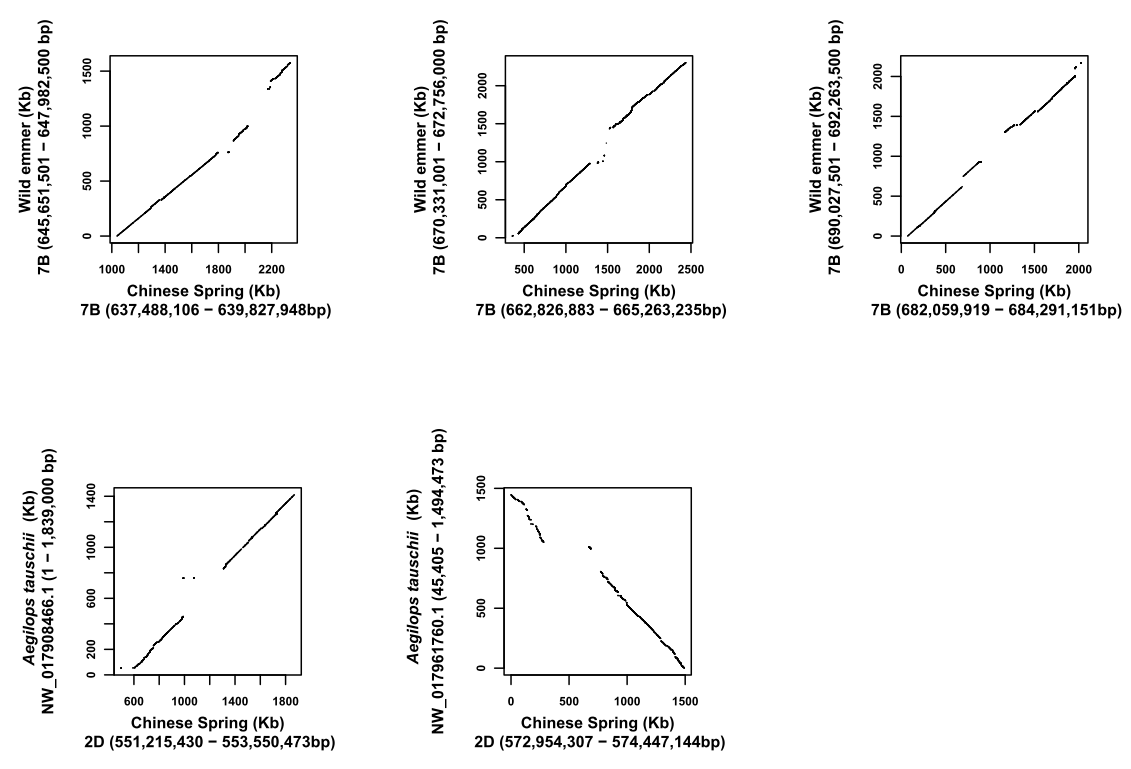


**Figure S27: The introgressied segments with accurate breakpoints identified in Chinese Spring.** Dot plot of 53 segments from Chinese Spring against the corresponding segments from wild emmer or *Aegilops tauschii* are presented. The positions in IWGSC RefSeq v1.0 and the pseudo-genome are provided in each independent figure.


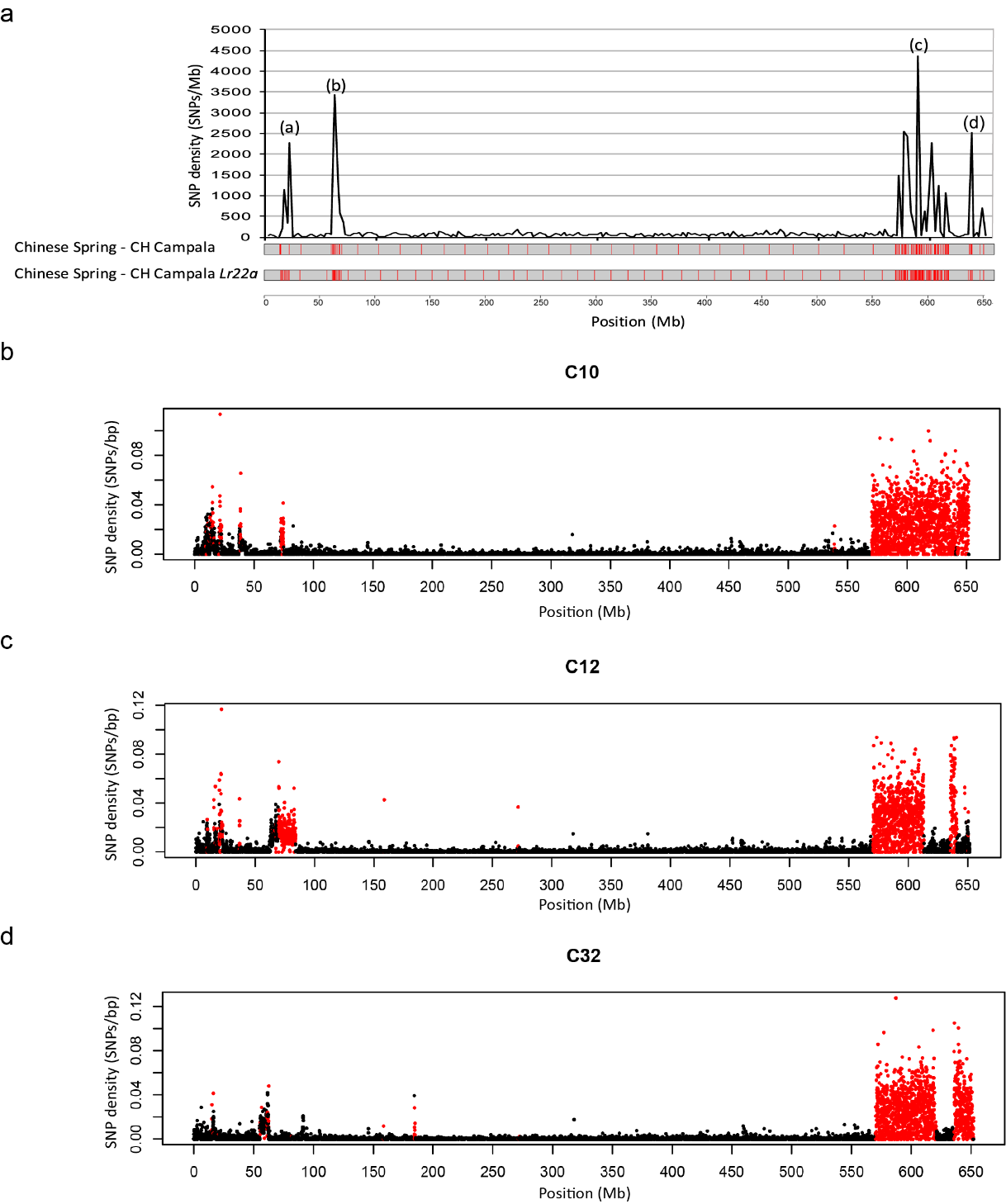


**Figure S28: Single nucleotide polymorphism (SNP) density across chromosome 2D of the Chinese Spring.** (a) SNP density between Chinese Spring and CH Campala Lr22a in a sliding window of 2.5 Mb [1]. The four introgressive haploblocks are indicated with letters a, b, c and d. (b), (c) and (d) Single nucleotide polymorphism (SNP) density of 3 resequencing accessions (C10, C12, and C32) of bread wheat. Each dot represents a 50 kb window. The dot in black represents the window which is neither deletion nor duplication, and the dot in red represent the window which is deleted in the accession.


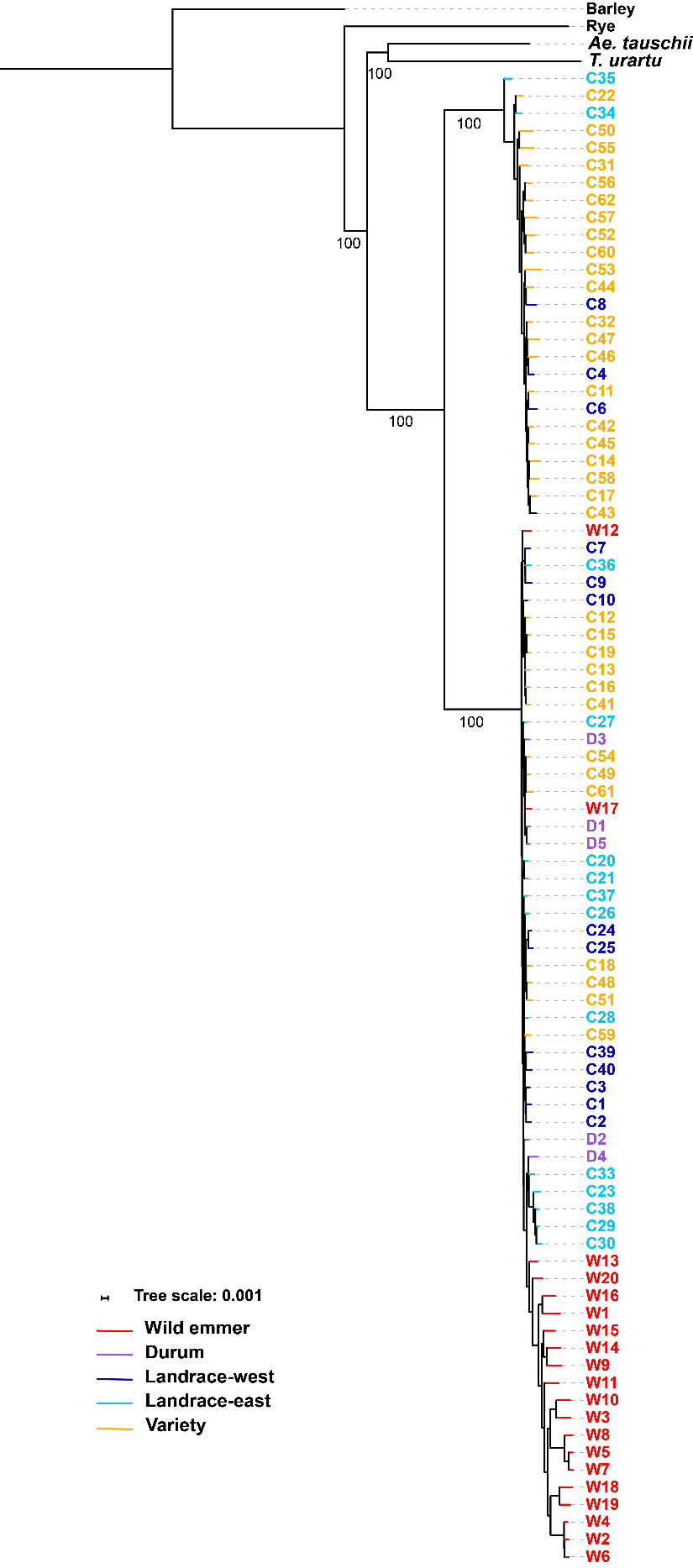
**Figure S29:** **The maximum likelihood tree of a 33 Mb introgression fragment on chromosome 5B from ~ 497 Mb to 529 Mb（853000 SNP）.** Several bread wheat accessions clustered in a clade alone, which were isolated from all wild emmer accessions.


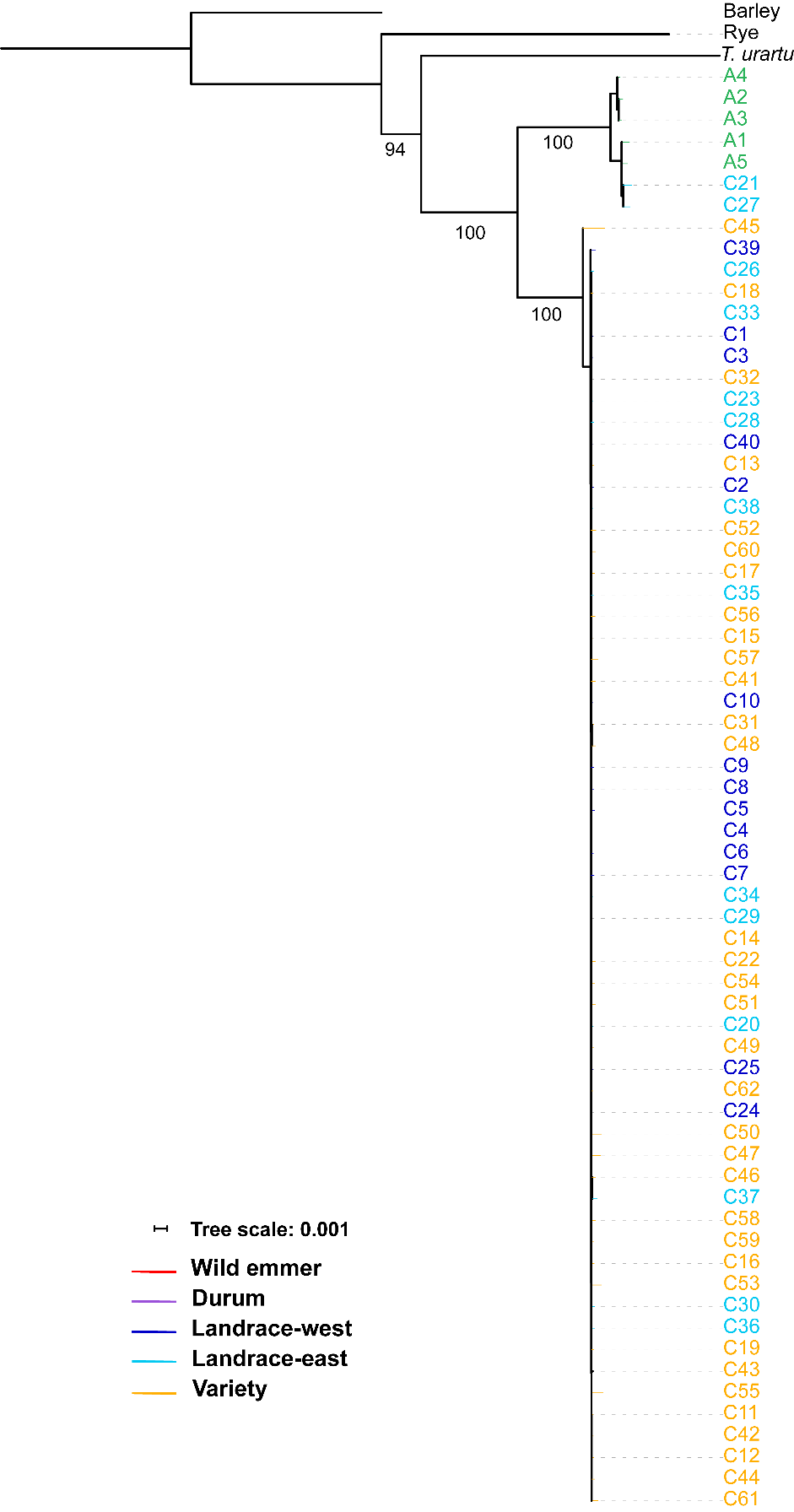


**Figure S30: The maximum likelihood tree of an introgression fragment from *Ae.tauschii*.** The introgression locate on chromosome 4D from 500, 862, 001 bp to 501, 999, 000 bp（46000 SNP）. Two landrace accessions and five *Ae.tauschii* clustered in a clade, indicating the these two accessions were likely introgressed by *Ae.tauschii* in this region.


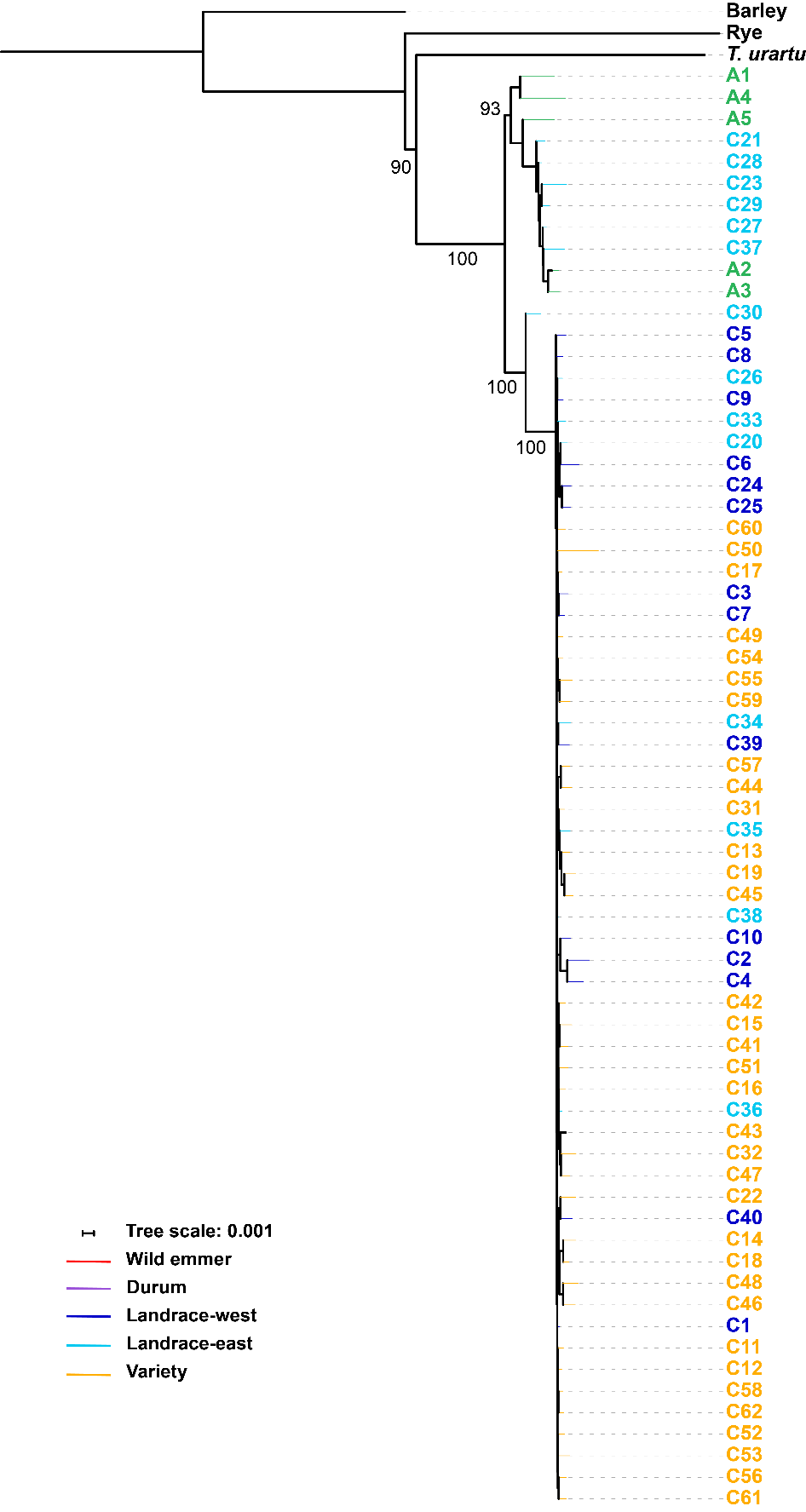


**Figure S31: The maximum likelihood tree of a introgression segment on chromosome 5D form 555, 981, 000 bp to 556, 438, 500 bp（70000 SNP）.** Six landrace accessions belong to landrace-east group clustered five *Ae.tauschii*, indicating the these six accessions were likely introgressed by *Ae.tauschii* in this region.


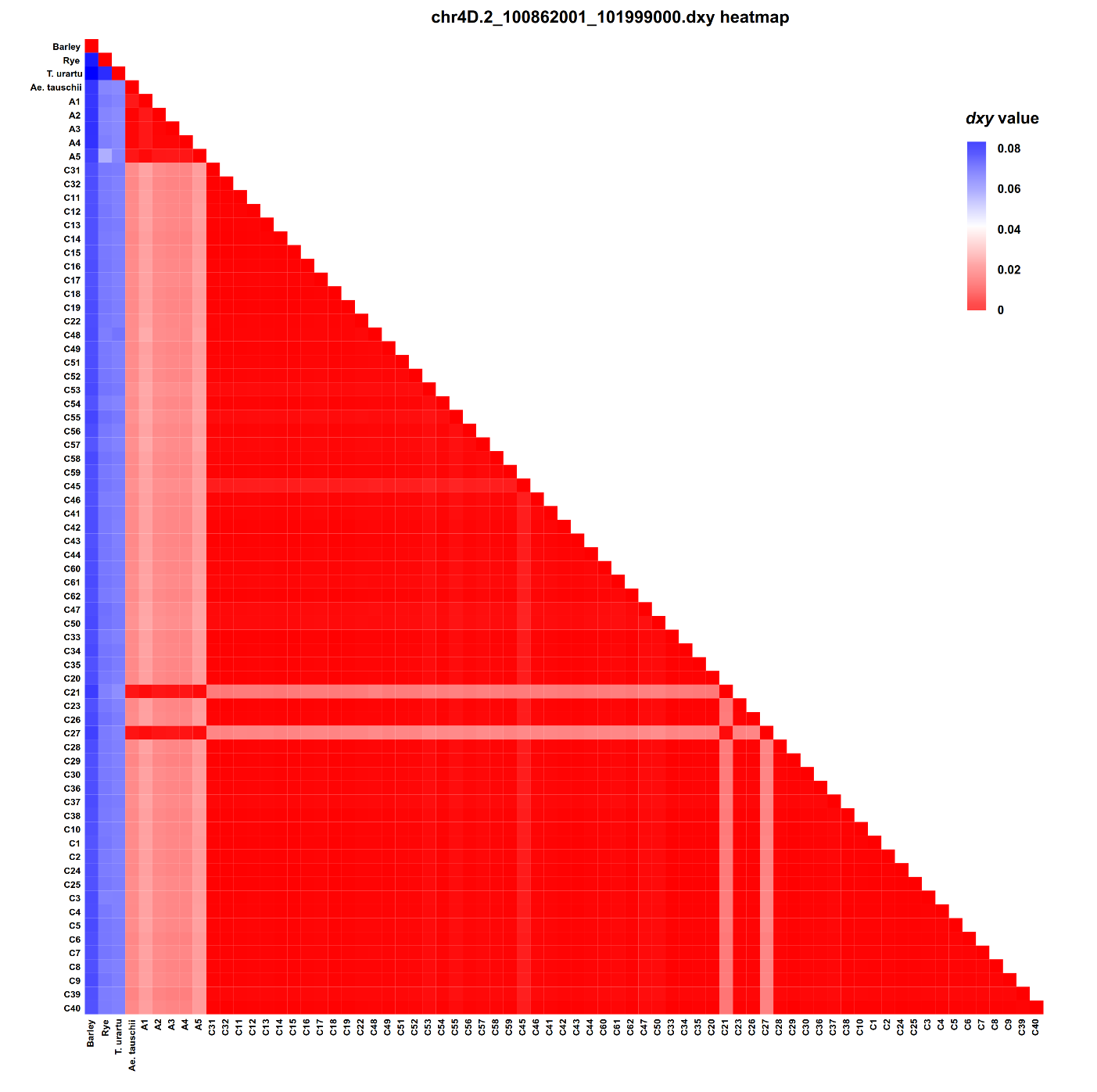


**Figure S32: Absolute sequence divergence *dxy* value (the number of pairwise differences per site) between each pairwise accessions on chromosome 4D from 500, 862, 001 bp to 501, 999, 000 bp.** C21 and C27, which were probably introgressed by *Ae.tauschii*, has the higher sequence similiraty with *Ae.tauschii* accessions than the bread wheat accessions.


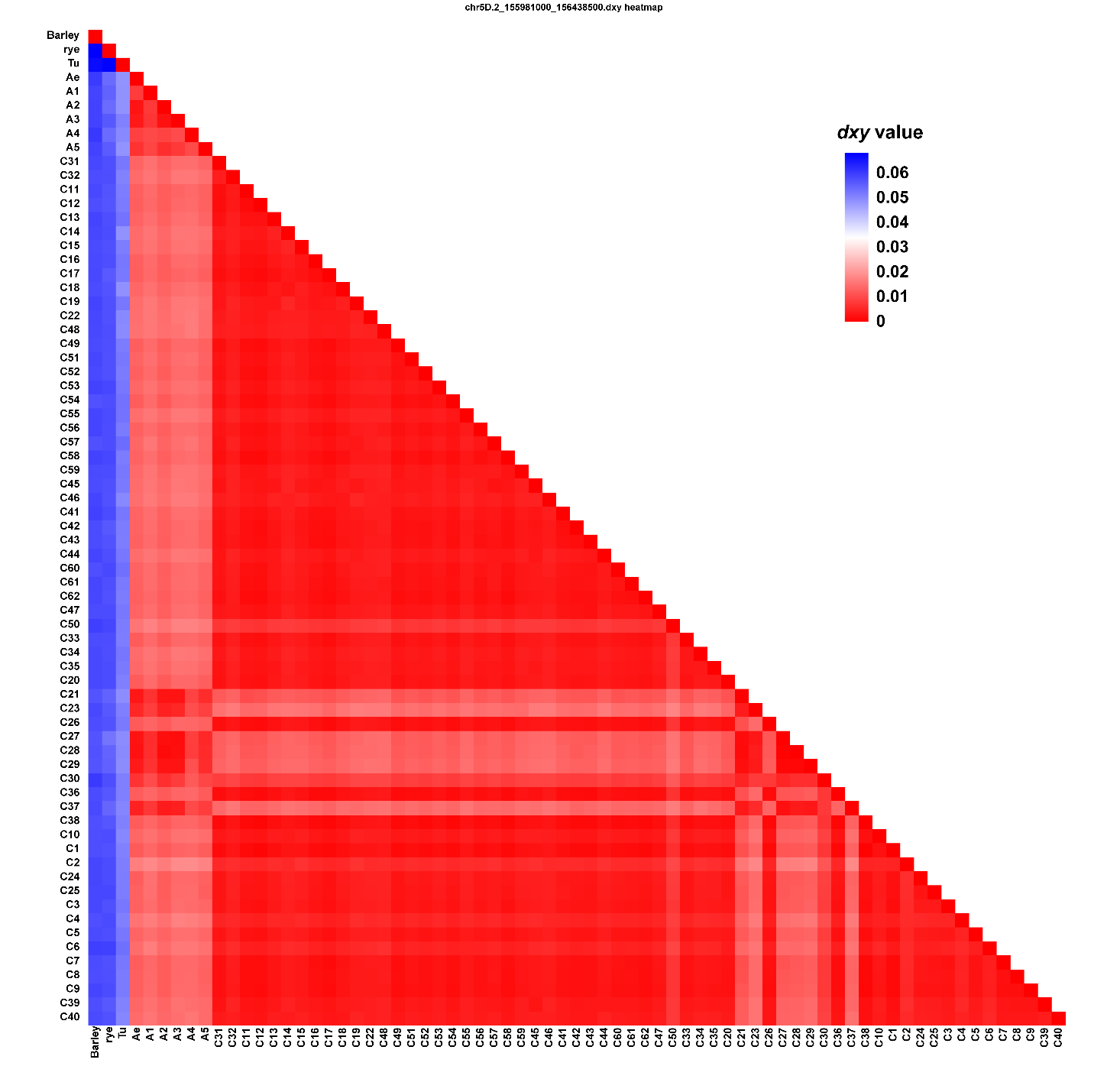


**Figure S33: Absolute sequence divergence *dxy* value (the number of pairwise differences per site) between each pairwise accessions on chromosome 5D form 555, 981, 000 bp to 556, 438, 500 bp.** Six landrace accessions (C21, C23, C27, C28, C29 and C37), which were probably introgressed by *Ae.tauschii*, has the higher sequence similiraty with *Ae.tauschii* accessions than the bread wheat accessions.


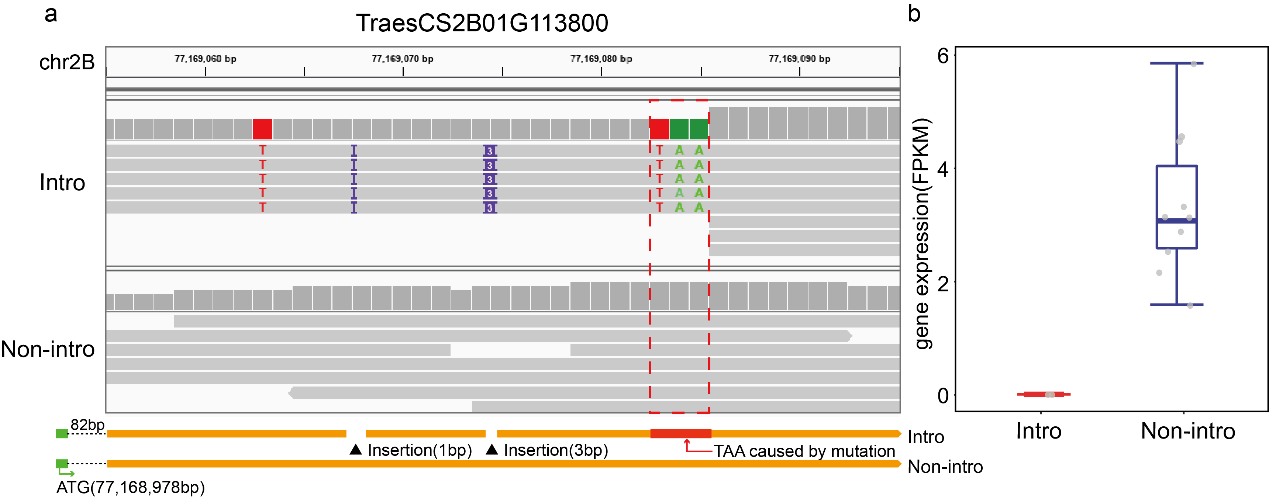
 **Figure S34：Comparison of mapping statistics and gene expression of TraesCS2B01G113800 in intro (introgression) and non-intro (non-introgression) accessions. (a) The genomic sequencing mapping statistics visualized by Integrative Genomics Viewer (IGV).** There were three consecutive mutations in the coding region in intro accessions, forming a stop codon that can result in premature termination of transcription. (**b) The boxplot of gene expression between intro and non-intro accessions.** The results showed that the expression (FPKM) of TraesCS2B01G113800 gene is nearly zero in intro accessions.

**
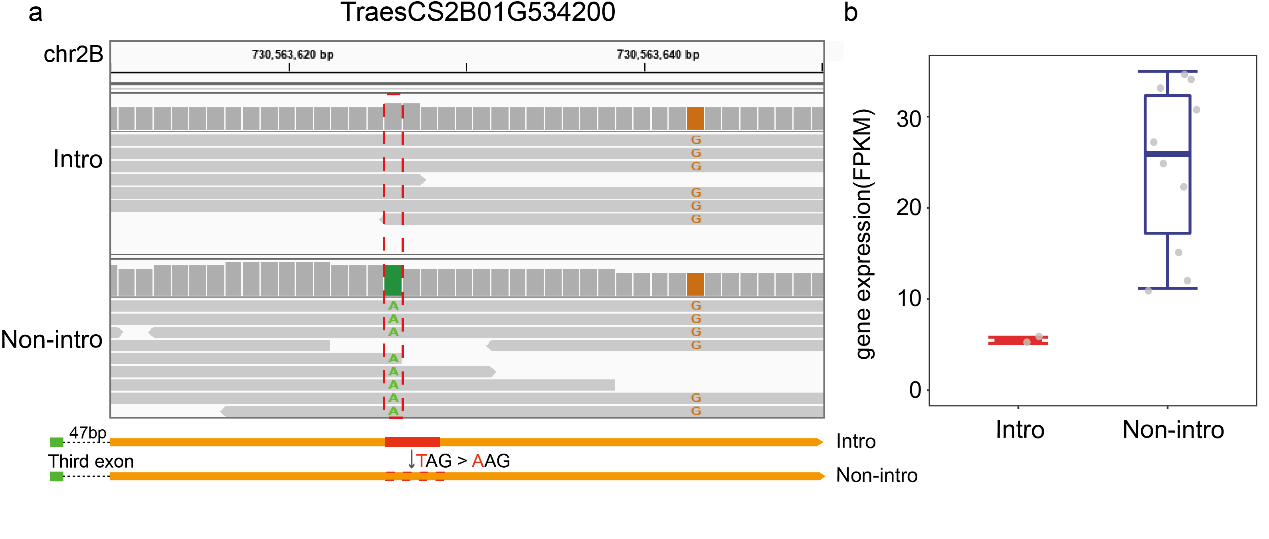
Figure S35：Comparison of mapping statistics and gene expression of TraesCS2B01G534200 in intro (introgression) and non-intro (non-introgression) accessions. (a) The genomic sequencing mapping statistics visualized by Integrative Genomics Viewer (IGV).** A mutation on the third exon in non-intro accessions made the stop codon (TAG) in intro accessions change to AAG (Lysine). (**b) The boxplot of gene expression between intro and non-intro accessions.** The results showed that the expression (FPKM) of TraesCS2B01G534200 gene is obviously lower in intro accessions than non-intro accessions.


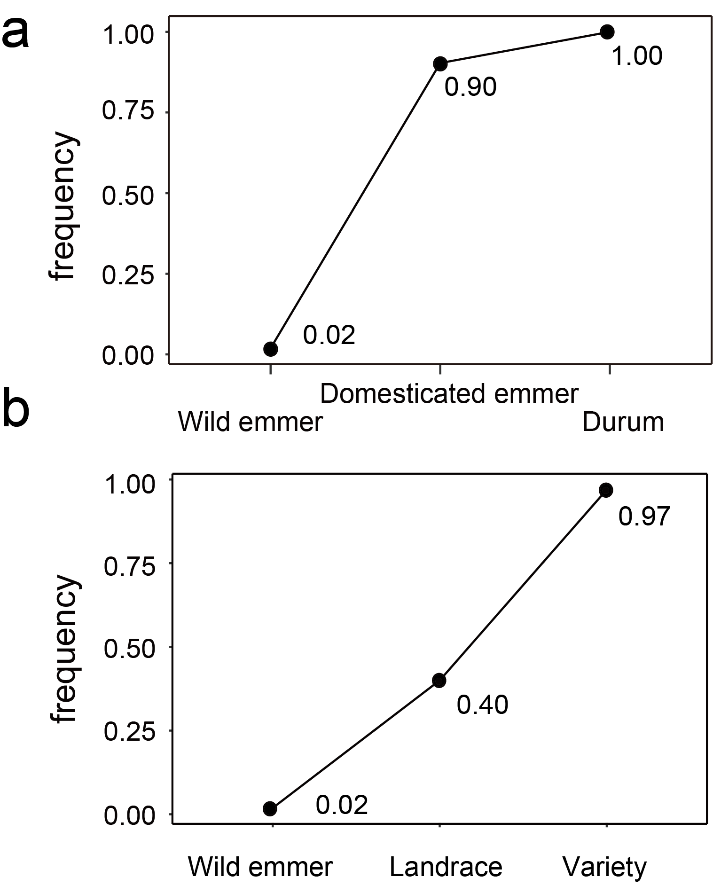


**Figure S36: (a)The frequency of haplotype-c in wild emmer, domesticated emmer, and durum wheat. (b)The frequency of haplotype-c in wild emmer, landrace, and variety.**


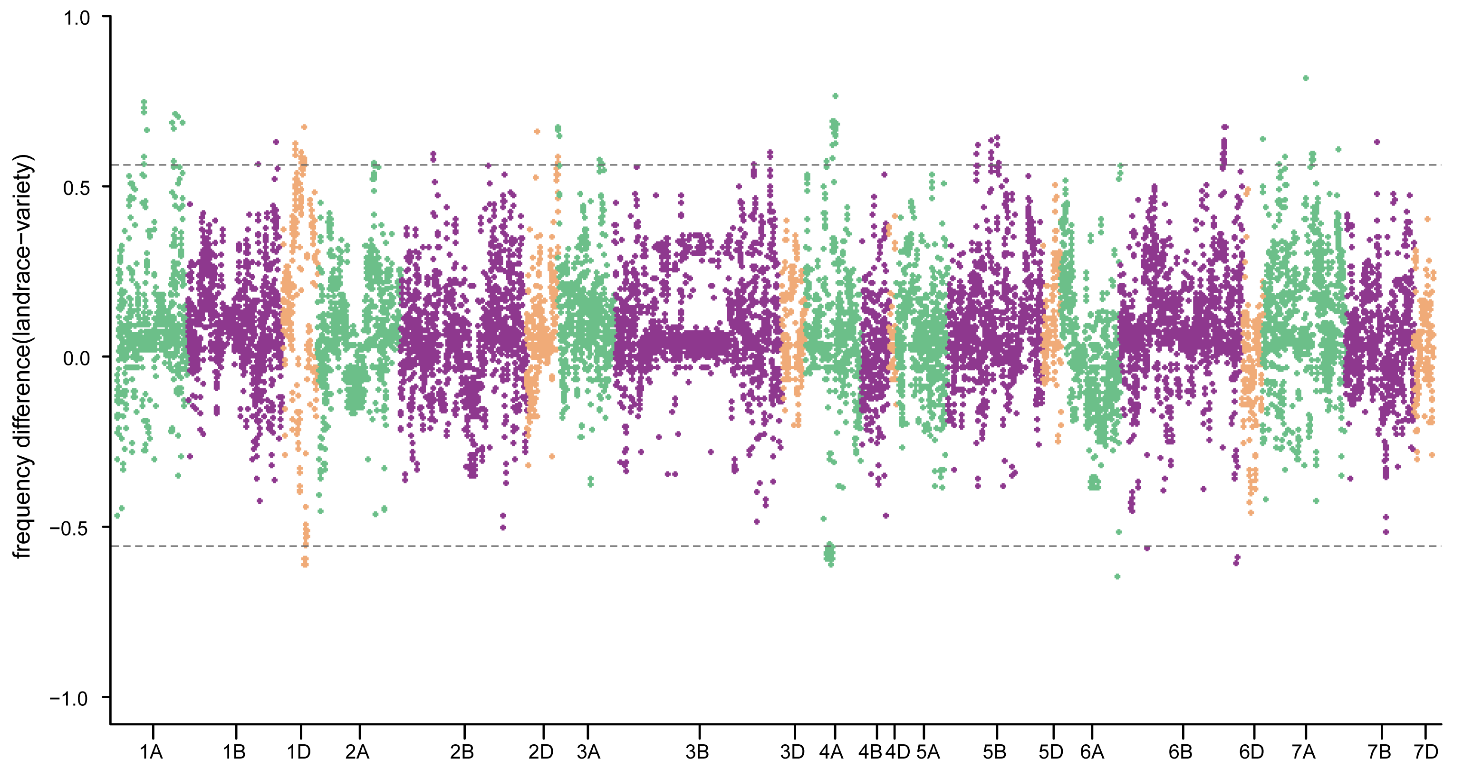


**Figure S37: Relative frequency difference (RFD) of CNVs between landraces and varieties.** The two horizontal dashed lines indicate the genome-wide threshold of selection signals, which showed the highest 1% RFD value (≥ 0.56 or ≤ -0.56).

**Table S1: Detailed information of the 93 accessions used in this study.** The * indicates that the data was downloaded from the public database. The reads of CS (Chinese Spring) and A1 were downloaded and generated during assembly of the IWGSC RefSeq v1.0 and *Ae.tauschii*. The raw coverage depth is given relative to the reference genome for the corresponding species. Reference homozygous, the number of homozygous reference sites; Alter homozygous, the number of homozygous nonreference sites, Heterozygosis, the number of heterozygous.

| **Sample** | **Genetic group** | **Average**  **depth (×)** | **Reference**  **homozygous** | **Heterozygosis** | **Alter**  **homozygous** | **Missing** | **Place** | **Description** |
| --- | --- | --- | --- | --- | --- | --- | --- | --- |
| A1* | *Ae. tauschii* | 83.042 | 5,989,226 | 371,421 | 9,960,047 | 170,697 | Caucasus | Diploid |
| A2 | *Ae. tauschii* | 21.322 | 5,260,323 | 1,911,381 | 9,113,612 | 206,075 | Caucasus | Diploid |
| A3 | *Ae. tauschii* | 12.533 | 5,679,683 | 467,176 | 10,083,884 | 260,648 | Caucasus | Diploid |
| A4 | *Ae. tauschii* | 20.312 | 5,624,196 | 1,537,984 | 9,116,741 | 212,470 | Caucasus | Diploid |
| A5 | *Ae. tauschii* | 18.529 | 5,854,056 | 378,091 | 9,893,345 | 365,899 | Caucasus | Diploid |
| W1 | Wild emmer | 9.270 | 48,762,089 | 648,896 | 17,463,284 | 1,230,134 | Israel | Tetraploid |
| W2 | Wild emmer | 11.272 | 46,708,806 | 774,700 | 19,800,622 | 820,275 | Israel | Tetraploid |
| W3 | Wild emmer | 10.620 | 47,976,920 | 653,316 | 18,122,085 | 1,352,082 | Israel | Tetraploid |
| W4 | Wild emmer | 9.463 | 47,231,914 | 719,464 | 18,942,378 | 1,210,647 | Israel | Tetraploid |
| W5 | Wild emmer | 11.762 | 39,360,212 | 699,594 | 26,501,433 | 1,543,164 | Israel | Tetraploid |
| W6 | Wild emmer | 8.349 | 46,419,017 | 712,344 | 19,263,004 | 1,710,038 | Israel | Tetraploid |
| W7 | Wild emmer | 11.504 | 39,395,103 | 733,293 | 26,553,164 | 1,422,843 | Israel | Tetraploid |
| W8 | Wild emmer | 11.639 | 46,923,077 | 752,800 | 19,264,077 | 1,164,449 | Israel | Tetraploid |
| W9 | Wild emmer | 8.960 | 48,093,493 | 651,875 | 17,704,705 | 1,654,330 | Israel | Tetraploid |
| W10 | Wild emmer | 11.036 | 48,315,510 | 697,758 | 17,720,154 | 1,370,981 | Israel | Tetraploid |
| W11 | Wild emmer | 9.653 | 44,104,402 | 11,480,027 | 11,046,624 | 1,473,350 | Syria | Tetraploid |
| W12 | Wild emmer | 8.944 | 55,182,996 | 438,205 | 11,230,330 | 1,252,872 | Turkey | Tetraploid |
| W13 | Wild emmer | 9.380 | 52,654,211 | 6,785,577 | 7,669,239 | 995,376 | Turkey | Tetraploid |
| W14 | Wild emmer | 10.501 | 46,546,280 | 9,259,665 | 11,365,392 | 933,066 | Israel | Tetraploid |
| W15 | Wild emmer | 12.902 | 45,005,165 | 9,825,988 | 12,813,973 | 459,277 | Israel | Tetraploid |
| W16 | Wild emmer | 10.648 | 45,848,890 | 7,803,045 | 13,575,560 | 876,908 | Syria | Tetraploid |
| W17 | Wild emmer | 11.392 | 46,700,521 | 8,921,633 | 11,675,381 | 806,868 | Bahrain | Tetraploid |
| W18 | Wild emmer | 6.726 | 47,087,730 | 6,237,418 | 13,225,246 | 1,554,009 | Jordan | Tetraploid |
| W19 | Wild emmer | 10.557 | 46,975,800 | 8,282,944 | 12,031,141 | 814,518 | Jordan | Tetraploid |
| W20 | Wild emmer | 10.770 | 46,919,183 | 11,080,562 | 9,144,289 | 960,369 | Lebanese | Tetraploid |
| D1 | Durum | 6.771 | 53,973,950 | 1,542,355 | 11,467,066 | 1,121,032 | Canada | Tetraploid |
| D2 | Durum | 6.709 | 53,858,772 | 1,697,381 | 11,387,163 | 1,161,087 | Canada | Tetraploid |
| D3 | Durum | 6.527 | 52,918,023 | 4,545,374 | 9,168,585 | 1,472,421 | Iraq | Tetraploid |
| D4 | Durum | 6.842 | 52,311,031 | 3,373,951 | 10,909,759 | 1,509,662 | Ireland | Tetraploid |
| D5 | Durum | 6.601 | 54,002,609 | 516,553 | 12,285,313 | 1,299,928 | Britain | Tetraploid |
| C20 | Landrace-east | 6.293 | 76,158,916 | 977,778 | 6,175,259 | 1,283,841 | Armenia | Hexaploid |
| C21 | Landrace-east | 6.371 | 75,459,248 | 2,716,479 | 5,156,174 | 1,263,893 | Armenia | Hexaploid |
| C23 | Landrace-east | 6.113 | 76,425,039 | 433,930 | 6,529,038 | 1,207,787 | Egypt | Hexaploid |
| C26 | Landrace-east | 6.158 | 75,569,808 | 432,666 | 7,018,555 | 1,574,765 | Afghanistan | Hexaploid |
| C27 | Landrace-east | 6.250 | 76,359,139 | 438,209 | 6,573,421 | 1,225,025 | Afghanistan | Hexaploid |
| C28 | Landrace-east | 6.390 | 76,646,335 | 465,989 | 6,441,998 | 1,041,472 | Afghanistan | Hexaploid |
| C29 | Landrace-east | 6.288 | 75,984,280 | 444,703 | 7,023,629 | 1,143,182 | Uzbekistan | Hexaploid |
| C30 | Landrace-east | 7.258 | 73,704,524 | 4,735,454 | 4,096,174 | 2,059,642 | Uzbekistan | Hexaploid |
| C33 | Landrace-east | 6.398 | 74,469,895 | 488,203 | 8,348,305 | 1,289,391 | India | Hexaploid |
| C34 | Landrace-east | 6.240 | 77,543,538 | 374,092 | 5,244,673 | 1,433,491 | India | Hexaploid |
| C35 | Landrace-east | 6.198 | 75,018,762 | 4,048,223 | 3,762,732 | 1,766,077 | India | Hexaploid |
| C36 | Landrace-east | 8.567 | 77,045,676 | 1,706,630 | 4,651,048 | 1,192,440 | Ji Country, Hebei China | Hexaploid |
| C37 | Landrace-east | 11.924 | 81,237,090 | 271,582 | 2,766,824 | 320,298 | Hangzhou, Zhejiang, China | Hexaploid |
| C38 | Landrace-east | 6.284 | 83,127,734 | 59,783 | 479,054 | 929,223 | Chengdu, Sichuan, China | Hexaploid |
| CS* | Landrace-east | 30.864 | 84,578,504 | 16,487 | 340 | 463 | China | Hexaploid |
| C1 | Landrace-west | 6.275 | 73,071,412 | 1,213,628 | 8,620,708 | 1,690,046 | Austria | Hexaploid |
| C10 | Landrace-west | 6.165 | 71,088,113 | 699,479 | 11,219,578 | 1,588,624 | Portugal | Hexaploid |
| C2 | Landrace-west | 6.341 | 73,557,205 | 795,535 | 8,814,901 | 1,428,153 | Austria | Hexaploid |
| C24 | Landrace-west | 6.367 | 71,454,000 | 699,186 | 11,247,495 | 1,195,113 | Jordan | Hexaploid |
| C25 | Landrace-west | 6.113 | 71,504,646 | 638,840 | 10,202,780 | 2,249,528 | Jordan | Hexaploid |
| C3 | Landrace-west | 6.383 | 73,058,027 | 1,329,758 | 8,656,999 | 1,551,010 | Austria | Hexaploid |
| C39 | Landrace-west | 12.134 | 74,221,867 | 5,873,270 | 4,132,469 | 368,188 | Heilongjiang, China | Hexaploid |
| C4 | Landrace-west | 6.244 | 73,029,229 | 549,432 | 9,487,665 | 1,529,468 | Bulgaria | Hexaploid |
| C40 | Landrace-west | 6.192 | 73,700,891 | 2,472,080 | 7,004,162 | 1,418,661 | Inner Mongolia, China | Hexaploid |
| C5 | Landrace-west | 6.274 | 70,329,480 | 6,141,417 | 6,211,606 | 1,913,291 | Andorra | Hexaploid |
| C6 | Landrace-west | 6.121 | 72,933,101 | 639,491 | 9,472,489 | 1,550,713 | Andorra | Hexaploid |
| C7 | Landrace-west | 6.220 | 74,255,596 | 562,940 | 8,392,174 | 1,385,084 | Spain | Hexaploid |
| C8 | Landrace-west | 6.226 | 70,213,665 | 777,031 | 11,699,067 | 1,906,031 | Italy | Hexaploid |
| C9 | Landrace-west | 6.268 | 72,053,861 | 558,437 | 10,550,360 | 1,433,136 | Portugal | Hexaploid |
| C11 | Variety | 6.369 | 74,863,195 | 668,841 | 7,724,528 | 1,339,230 | America | Hexaploid |
| C12 | Variety | 6.016 | 75,284,154 | 671,756 | 6,933,262 | 1,706,622 | America | Hexaploid |
| C13 | Variety | 6.045 | 74,528,391 | 498,952 | 8,261,689 | 1,306,762 | America | Hexaploid |
| C14 | Variety | 6.228 | 74,890,480 | 1,071,621 | 7,144,853 | 1,488,840 | America | Hexaploid |
| C15 | Variety | 6.135 | 75,699,447 | 557,230 | 6,843,420 | 1,495,697 | America | Hexaploid |
| C16 | Variety | 6.396 | 73,824,881 | 897,945 | 8,561,722 | 1,311,246 | America | Hexaploid |
| C17 | Variety | 6.262 | 75,140,244 | 475,979 | 7,826,751 | 1,152,820 | America | Hexaploid |
| C18 | Variety | 6.141 | 75,822,325 | 1,296,481 | 6,006,850 | 1,470,138 | America | Hexaploid |
| C19 | Variety | 6.205 | 76,174,600 | 630,876 | 6,638,963 | 1,151,355 | America | Hexaploid |
| C22 | Variety | 6.284 | 76,209,869 | 482,632 | 6,815,258 | 1,088,035 | Egypt | Hexaploid |
| C31 | Variety | 6.113 | 72,929,376 | 5,333,515 | 4,496,201 | 1,836,702 | India | Hexaploid |
| C32 | Variety | 6.145 | 75,509,165 | 539,478 | 7,311,463 | 1,235,688 | India | Hexaploid |
| C41 | Variety | 6.290 | 75,631,108 | 447,265 | 7,334,106 | 1,183,315 | Jiangsu, China | Hexaploid |
| C42 | Variety | 6.088 | 74,745,042 | 449,777 | 7,036,479 | 2,364,496 | Henan, China | Hexaploid |
| C43 | Variety | 9.399 | 76,808,353 | 506,181 | 6,475,993 | 805,267 | Shaanxi, China | Hexaploid |
| C44 | Variety | 9.629 | 77,966,218 | 385,689 | 5,377,658 | 866,229 | Shandong, China | Hexaploid |
| C45 | Variety | 8.694 | 75,238,912 | 434,632 | 7,643,218 | 1,279,032 | Sichuan, China | Hexaploid |
| C46 | Variety | 8.806 | 75,577,580 | 489,581 | 6,380,911 | 2,147,722 | Henan, China | Hexaploid |
| C47* | Variety | 16.219 | 75,127,967 | 847,193 | 6,670,765 | 1,949,869 | Australia | Hexaploid |
| C48* | Variety | 10.061 | 57,952,654 | 359,747 | 6,533,999 | 19,749,394 | Australia | Hexaploid |
| C49* | Variety | 9.278 | 74,282,240 | 572,782 | 8,635,959 | 1,104,813 | Australia | Hexaploid |
| C50* | Variety | 14.742 | 74,414,519 | 834,876 | 7,841,617 | 1,504,782 | Australia | Hexaploid |
| C51* | Variety | 22.039 | 72,831,343 | 890,747 | 7,848,785 | 3,024,919 | Australia | Hexaploid |
| C52* | Variety | 15.979 | 75,393,096 | 678,582 | 7,699,221 | 824,895 | Australia | Hexaploid |
| C53* | Variety | 12.064 | 75,993,491 | 682,422 | 7,806,120 | 113,761 | Australia | Hexaploid |
| C54* | Variety | 11.501 | 72,058,860 | 537,799 | 7,558,422 | 4,440,713 | Australia | Hexaploid |
| C55* | Variety | 12.541 | 76,452,633 | 659,824 | 7,395,521 | 87,816 | Australia | Hexaploid |
| C56* | Variety | 11.260 | 76,067,698 | 853,118 | 7,191,600 | 483,378 | Australia | Hexaploid |
| C57* | Variety | 16.168 | 64,277,548 | 405,068 | 6,173,359 | 13,739,819 | Australia | Hexaploid |
| C58* | Variety | 14.001 | 72,188,682 | 599,744 | 7,148,655 | 4,658,713 | Australia | Hexaploid |
| C59* | Variety | 56.832 | 76,261,864 | 690,021 | 7,614,748 | 29,161 | Australia | Hexaploid |
| C60* | Variety | 11.054 | 72,733,013 | 872,177 | 7,844,891 | 3,145,713 | Australia | Hexaploid |
| C61* | Variety | 15.756 | 76,818,148 | 779,007 | 6,672,980 | 325,659 | Australia | Hexaploid |
| C62* | Variety | 22.602 | 75,897,871 | 646,373 | 7,988,329 | 63,221 | Australia | Hexaploid |

**Table S2: Statistics of the whole-genome SNPs and indels.** Approximately half of the SNPs in the A and B subgenomes in wild emmer disappeared in bread wheat. While in the D genome, about 79% of the SNPs of *Ae.* *tauschii* were not found in bread wheat.

| Sequence variants | | A subgenome | | |  | B subgenome | | |  | D subgenome | | |
| --- | --- | --- | --- | --- | --- | --- | --- | --- | --- | --- | --- | --- |
|  |  | Wild emmer | Landraces | Varieties |  | Wild emmer | Landraces | Varieties |  | *Ae. tauschii* | Landraces | Varieties |
| SNPs | Total | 31,487,354 | 12,811,555 | 11,185,051 |  | 29,678,230 | 14,570,377 | 12,572,860 |  | 14,980,095 | 3,061,733 | 2,919,016 |
|  | Intergenic | 31,006,532 | 12,565,338 | 10,962,177 |  | 29,210,573 | 14,318,458 | 12,340,428 |  | 14,744,029 | 2,952,452 | 2,818,680 |
|  | Intron | 320,583 | 158,815 | 142,652 |  | 306,554 | 161,873 | 148,965 |  | 152,816 | 62,018 | 55,794 |
|  | Exon | 160,239 | 87,402 | 80,222 |  | 161,103 | 90,046 | 83,467 |  | 83,250 | 47,263 | 44,542 |
| Indels | Total | 3,944,099 | 2,060,123 | 1,951,798 |  | 4,352,845 | 2,501,420 | 2,333,969 |  | 2,568,465 | 913,821 | 867,455 |
|  | Insertions | 1,617,455 | 930,531 | 876,260 |  | 1,753,508 | 1,105,522 | 1,023,315 |  | 1,230,580 | 433,697 | 403,337 |
|  | Deletions | 2,326,644 | 1,129,592 | 1,075,538 |  | 2,599,337 | 1,395,898 | 1,310,654 |  | 1,337,885 | 480,124 | 464,118 |

**Table S3: The accuracy of the SNPs calling determined by wheat 660K array.**

| **Individual (hexaploid)** | **C43** | **C38** | **C45** | **C37** |
| --- | --- | --- | --- | --- |
| Matched | 233,202 | 231,148 | 232,297 | 233,493 |
| Unmatched | 9,232 | 11,508 | 8,458 | 11,006 |
| Count | 242,434 | 242,656 | 240,755 | 244,499 |
| Accuracy (except for missing sites) | 96.19 % | 95.26 % | 96.49 % | 95.50 % |

**Table S10:** **Detailed information of the exon capture data of wheat used in this study.**

| **Number** | **Accession** | **Population** | **Place** | **Description** |
| --- | --- | --- | --- | --- |
| 1 | WE-1 | Wild emmer | Central Israel | Tetraploid |
| 2 | WE-2 | Wild emmer | Northern Israel | Tetraploid |
| 3 | WE-3 | Wild emmer | Northern Israel | Tetraploid |
| 4 | WE-4 | Wild emmer | Central Israel | Tetraploid |
| 5 | WE-5 | Wild emmer | Northern Israel | Tetraploid |
| 6 | WE-6 | Wild emmer | Kazin, Syria | Tetraploid |
| 7 | WE-7 | Wild emmer | Northern Israel | Tetraploid |
| 8 | WE-8 | Wild emmer | Northern Israel | Tetraploid |
| 9 | WE-9 | Wild emmer | Northern Israel | Tetraploid |
| 10  11 | WE-10  WE-11 | Wild emmer  Wild emmer | Central Israel  Northern Israel | Tetraploid  Tetraploid |
| 12 | WE-12 | Wild emmer | Northern Israel | Tetraploid |
| 13 | WE-13 | Wild emmer | Northern Israel | Tetraploid |
| 14 | WE-14 | Wild emmer | Central Israel | Tetraploid |
| 15 | WE-15 | Wild emmer | Central Israel | Tetraploid |
| 16 | WE-16 | Wild emmer | Central Lebanon | Tetraploid |
| 17 | WE-17 | Wild emmer | Central Lebanon | Tetraploid |
| 18 | WE-18 | Wild emmer | Northern Israel | Tetraploid |
| 19 | WE-19 | Wild emmer | Central Israel | Tetraploid |
| 20 | WE-20 | Wild emmer | Halab, Syria | Tetraploid |
| 21 | WE-21 | Wild emmer | Iraq | Tetraploid |
| 22 | WE-22 | Wild emmer | Central Lebanon | Tetraploid |
| 23 | WE-23 | Wild emmer | Central Turkey | Tetraploid |
| 24 | WE-24 | Wild emmer | Diyarbakir, Turkey | Tetraploid |
| 25 | WE-25 | Wild emmer | Karacadag, Turkey | Tetraploid |
| 26 | WE-26 | Wild emmer | Diyarbakir, Turkey | Tetraploid |
| 27 | WE-27 | Wild emmer | Urfa, Turkey | Tetraploid |
| 28 | WE-28 | Wild emmer | Diyarbakir, Turkey | Tetraploid |
| 29 | WE-29 | Wild emmer | Diyarbakir, Turkey | Tetraploid |
| 30 | WE-30 | Wild emmer | Diyarbakir, Turkey | Tetraploid |
| 31 | WE-31 | Wild emmer | Central Turkey | Tetraploid |
| 32 | WE-32 | Wild emmer | Diyarbakir, Turkey | Tetraploid |
| 33 | WE-33 | Wild emmer | Diyarbakir, Turkey | Tetraploid |
| 34 | WE-34 | Wild emmer | Diyarbakir, Turkey | Tetraploid |
| 35 | DE-1 | Domesticated emmer | Oman | Tetraploid |
| 36 | DE-2 | Domesticated emmer | India | Tetraploid |
| 37 | DE-3 | Domesticated emmer | Central Turkey | Tetraploid |
| 38 | DE-4 | Domesticated emmer | Central Turkey | Tetraploid |
| 39 | DE-5 | Domesticated emmer | Central Israel | Tetraploid |
| 40 | DE-6 | Domesticated emmer | Southern Turkey | Tetraploid |
| 41 | DE-7 | Domesticated emmer | Central Israel | Tetraploid |
| 42 | DE-8 | Domesticated emmer | Central Israel | Tetraploid |
| 43 | DE-9 | Domesticated emmer | Syria | Tetraploid |
| 44 | DE-10 | Domesticated emmer | Central Israel | Tetraploid |
| 45 | DE-11 | Domesticated emmer | Southern Turkey | Tetraploid |
| 46 | DE-12 | Domesticated emmer | Central Turkey | Tetraploid |
| 47 | DE-13 | Domesticated emmer | Syria | Tetraploid |
| 48 | DE-14 | Domesticated emmer | Italy | Tetraploid |
| 49 | DE-15 | Domesticated emmer | Spain | Tetraploid |
| 50 | DE-16 | Domesticated emmer | Spain | Tetraploid |
| 51 | DE-17 | Domesticated emmer | Central Turkey | Tetraploid |
| 52 | DE-18 | Domesticated emmer | Central Turkey | Tetraploid |
| 53 | DE-19 | Domesticated emmer | Ukraine | Tetraploid |
| 54 | DE-20 | Domesticated emmer | Slovenia | Tetraploid |
| 55 | DE-21 | Domesticated emmer | Croatia | Tetraploid |
| 56 | DE-22 | Domesticated emmer | Bosnia and Herzegovina | Tetraploid |
| 57 | DE-23 | Domesticated emmer | Iran | Tetraploid |
| 58 | DE-24 | Domesticated emmer | Iran | Tetraploid |
| 59 | DE-25 | Domesticated emmer | Iran | Tetraploid |
| 60 | DE-26 | Domesticated emmer | Central Turkey | Tetraploid |
| 61 | DE-27 | Domesticated emmer | Central Turkey | Tetraploid |
| 62 | DE-28 | Domesticated emmer | Armenia | Tetraploid |
| 63 | DE-29 | Domesticated emmer | Central Turkey | Tetraploid |
| 64 | DE-230 | Domesticated emmer | Georgia | Tetraploid |
| 65 | DE-31 | Domesticated emmer | Iran | Tetraploid |
| 66 | PI366716 | Landrace | Afghanistan | Hexaploid |
| 67 | PI406517 | Landrace | Nepal | Hexaploid |
| 68 | PI349512 | Landrace | Switzerland | Hexaploid |
| 69 | PI481923 | Landrace | Sudan | Hexaploid |
| 70 | PI481718 | Landrace | Bhutan | Hexaploid |
| 71 | PI382150 | Landrace | Japan | Hexaploid |
| 72 | PI366905 | Landrace | Afghanistan | Hexaploid |
| 73 | PI470817 | Landrace | Algeria | Hexaploid |
| 74 | PI445736 | Landrace | Nepal | Hexaploid |
| 75 | PI477870 | Landrace | Peru | Hexaploid |
| 76 | PI262611 | Landrace | Turkmenistan | Hexaploid |
| 77 | PI222669 | Landrace | Iran | Hexaploid |
| 78 | PI278297 | Landrace | Greece | Hexaploid |
| 79 | PI210945 | Landrace | Cyprus | Hexaploid |
| 80 | PI192569 | Landrace | Sweden | Hexaploid |
| 81 | PI192147 | Landrace | Ethiopia | Hexaploid |
| 82 | PI8813 | Landrace | Iraq | Hexaploid |
| 83 | PI565213 | Landrace | Bolivia | Hexaploid |
| 84 | PI82469 | Landrace | North Korea | Hexaploid |
| 85 | PI185715 | Landrace | Portugal | Hexaploid |
| 86 | PI245368 | Landrace | Guatemala | Hexaploid |
| 87 | PI166333 | Landrace | Turkey | Hexaploid |
| 88 | PI166180 | Landrace | India | Hexaploid |
| 89 | PI177943 | Landrace | Turkey | Hexaploid |
| 90 | PI192001 | Landrace | Angola | Hexaploid |
| 91 | PI153785 | Landrace | Brazil | Hexaploid |

**Table S11:** **Two groups of the landraces, Landrace-west and Landrace-east.**

| **Subpopulations** | **Accessions** |
| --- | --- |
| Landrace-west | C1, C2, C3, C4, C5, C6, C7, C8, C9, C10, C24, C25, C39, C40 |
| Landrace-east | C20, C21, C23, C26, C27, C28, C29, C30, C33, C34, C35, CS, C36, C37, C38 |

**Table S14: The list of differentially expressed genes (DEGs) between intro and non-intro accessions.** The cells with yellow indicate introgression. In total, 18 DEGs (T-test, corrected P<0.05) between introgression and non-introgression accessions were identified, of which 12 genes are up-regulated expression in non-intro accession, while 6 genes showed higher expression in intro accessions compared to non-intro accession

| **Chrom** | **Start** | **End** | **Gene** | **Description** | **C37-root** | **C38-root** | **C39-root** | **C41-root** | **C43-root** | **C44-root** | **C37-leaf** | **C38-leaf** | **C39-leaf** | **C41-leaf** | **C43-leaf** | **C44-leaf** | **corrected P-value** |
| --- | --- | --- | --- | --- | --- | --- | --- | --- | --- | --- | --- | --- | --- | --- | --- | --- | --- |
| chr1B | 94,827,174 | 94,832,377 | TraesCS1B01G093300.1 | G-patch domain containing protein | 0.13 | 0.15 | 0.30 | 0.19 | 2.27 | 0.43 | 0.09 | 0.38 | 0.03 | 0.15 | 1.25 | 1.72 | 0.043746 |
| chr1B | 94,827,504 | 94,832,377 | TraesCS1B01G093300.2 | G-patch domain containing protein | 3.12 | 3.23 | 2.09 | 3.96 | 0.00 | 0.00 | 4.44 | 1.72 | 4.22 | 1.69 | 0.00 | 0.00 | 0.024276 |
| chr1B | 678,262,880 | 678,267,139 | TraesCS1B01G469400.1 | Protein kinase family protein | 1.29 | 0.07 | 0.09 | 0.09 | 0.89 | 2.26 | 1.34 | 0.12 | 0.08 | 0.09 | 0.91 | 1.18 | 0.03509 |
| chr5B | 448,130,568 | 448,132,821 | TraesCS5B01G263700.2 | Pectin lyase-like superfamily protein | 0.00 | 0.00 | 6.77 | 4.45 | 9.38 | 7.66 | 0.00 | 0.00 | 14.24 | 12.22 | 15.57 | 7.73 | 0.03509 |
| chr5B | 501,237,516 | 501,239,700 | TraesCS5B01G317300.1 | Mediator of RNA polymerase II transcription subunit 10 | 0.14 | 0.16 | 0.16 | 0.15 | 2.40 | 3.92 | 0.46 | 0.19 | 0.33 | 0.09 | 2.40 | 1.66 | 0.007563 |
| chr5B | 502,501,258 | 502,508,421 | TraesCS5B01G318400.1 | Transducin/WD40 repeat protein | 7.67 | 4.25 | 5.06 | 4.02 | 1.33 | 0.57 | 6.39 | 4.80 | 4.32 | 1.66 | 0.00 | 1.02 | 0.047358 |
| chr5B | 503,231,211 | 503,235,331 | TraesCS5B01G319100.1 | Altered inheritance of mitochondria protein | 1.22 | 2.27 | 0.60 | 0.75 | 3.54 | 4.13 | 1.33 | 1.40 | 0.80 | 1.73 | 3.65 | 1.76 | 0.047358 |
| chr5B | 506,503,983 | 506,513,402 | TraesCS5B01G320900.1 | Pantothenate kinase, putative | 10.87 | 11.89 | 11.95 | 10.27 | 7.47 | 6.69 | 14.73 | 14.18 | 15.78 | 12.76 | 7.89 | 3.87 | 0.024276 |
| chr5B | 507,006,223 | 507,008,901 | TraesCS5B01G322200.1 | Acyl-[acyl-carrier-protein] desaturase | 6.52 | 6.01 | 6.74 | 3.07 | 0.00 | 0.06 | 4.30 | 3.46 | 3.38 | 3.50 | 0.00 | 0.00 | 0.019965 |
| chr5B | 508,777,182 | 508,778,873 | TraesCS5B01G324700.1 | RNA-binding (RRM/RBD/RNP motifs) family protein | 9.64 | 10.99 | 7.77 | 8.23 | 1.21 | 0.88 | 6.96 | 4.18 | 3.94 | 3.64 | 0.00 | 0.29 | 0.047358 |
| chr5B | 508,795,398 | 508,798,533 | TraesCS5B01G324800.3 | Triosephosphate isomerase | 0.15 | 0.17 | 0.20 | 0.10 | 48.97 | 29.19 | 0.10 | 0.14 | 0.92 | 0.28 | 104.24 | 61.63 | 0.019965 |
| chr5B | 511,695,246 | 511,697,474 | TraesCS5B01G328200.1 | Armadillo repeat only | 6.01 | 4.61 | 6.05 | 4.66 | 2.17 | 1.16 | 3.95 | 3.66 | 5.55 | 3.58 | 0.78 | 0.87 | 0.013915 |
| chr5B | 524,553,019 | 524,554,054 | TraesCS5B01G340100.1 | ATPase family associated with various cellular activities | 2.74 | 2.06 | 2.76 | 3.64 | 0.00 | 0.00 | 3.59 | 2.47 | 2.13 | 1.52 | 0.00 | 0.09 | 0.008873 |
| chr5B | 528,590,211 | 528,591,278 | TraesCS5B01G347600.1 | Protein PHLOEM PROTEIN 2-LIKE A10 | 1.64 | 1.38 | 1.53 | 1.40 | 0.07 | 0.23 | 0.80 | 0.62 | 0.84 | 1.67 | 0.08 | 0.00 | 0.024402 |
| chr5B | 694,519,059 | 694,526,604 | TraesCS5B01G539500.1 | Transducin/WD-like repeat-protein | 0.09 | 1.32 | 0.70 | 2.43 | 2.71 | 0.33 | 0.66 | 2.94 | 0.94 | 1.15 | 2.64 | 0.63 | 0.03509 |
| chr7A | 67,984,029 | 67,989,366 | TraesCS7A01G110700.1 | NBS-LRR-like resistance protein | 3.97 | 7.92 | 2.92 | 0.03 | 0.05 | 3.33 | 2.49 | 2.67 | 4.00 | 0.00 | 0.01 | 3.92 | 0.047358 |
| chr7A | 68,087,481 | 68,094,141 | TraesCS7A01G111300.1 | Mitogen-activated protein kinase | 0.07 | 0.06 | 0.07 | 5.27 | 5.98 | 0.10 | 0.22 | 0.12 | 0.18 | 3.39 | 2.79 | 0.09 | 0.00484 |
| chr7A | 68,188,195 | 68,190,915 | TraesCS7A01G111400.1 |  | 2.71 | 2.99 | 4.40 | 1.33 | 0.77 | 3.70 | 2.66 | 2.93 | 1.99 | 1.16 | 0.35 | 1.89 | 0.047358 |

**Table S18: Summary of the KEGG enrichment analysis of genes located in selective regions during domestication process.**

| **KEGG pathway** | **Group count** | **Total count** | **Corrected *P*-value** | **Genes** |
| --- | --- | --- | --- | --- |
| Flavonoid biosynthesis | 9 | 21 | 0.000185853 | *TraesCS1D01G310000, TraesCS4D01G339800, TraesCS5A01G475600, TraesCS4D01G019300, TraesCS4D01G339900, TraesCS3A01G342900, TraesCS4A01G002900, TraesCS7D01G089300, TraesCS5D01G342400* |
| Phenylpropanoid biosynthesis | 21 | 157 | 0.000609871 | *TraesCS1D01G310000, TraesCS7D01G090500, TraesCS4D01G337300, TraesCS7B01G274500, TraesCS7B01G275200, TraesCS2B01G401600, TraesCS7A01G369400, TraesCS4B01G041200, TraesCS5D01G488900, TraesCS3A01G337700, TraesCS3A01G342900, TraesCS5D01G488800, TraesCS4A01G002900, TraesCS7D01G090600, TraesCS7D01G090300, TraesCS7B01G275400, TraesCS5D01G342400, TraesCS5D01G438300, TraesCS7D01G090200, TraesCS7B01G275000, TraesCS4B01G041100* |
| Metabolic pathways | 115 | 1910 | 0.004627491 | *TraesCS3A01G430000, TraesCS5D01G344900, TraesCS3D01G052700, TraesCS5A01G454500, TraesCS7B01G275400, TraesCS1D01G013500, TraesCS5D01G506900, TraesCS2B01G276300, TraesCS2A01G425100, TraesCS5B01G493400, TraesCS7B01G250400, TraesCS5D01G488800, TraesCS6D01G330700, TraesCS4A01G002500, TraesCS5A01G177400, TraesCS4A01G043800, TraesCS7D01G090500, TraesCS3D01G052800, TraesCS2B01G101700, TraesCS2B01G401600, TraesCS3A01G338600, TraesCS7D01G221000, TraesCS5A01G480500, TraesCS5A01G465200, TraesCS3B01G438700, TraesCS7A01G383200, TraesCS3B01G435500, TraesCS3A01G430200, TraesCS5A01G475600, TraesCS3A01G430400, TraesCS5A01G461500, TraesCS4A01G002800, TraesCS5A01G476700, TraesCS7A01G384000, TraesCS3A01G337700, TraesCS7D01G361000, TraesCS4B01G222100, TraesCS7D01G089300, TraesCS4D01G320700, TraesCS7A01G382500, TraesCS5A01G473400, TraesCS4D01G339900, TraesCS1D01G013400, TraesCS3A01G430300, TraesCS5A01G411200, TraesCS7D01G090200, TraesCS6A01G264700, TraesCS5A01G461900, TraesCS6A01G358100, TraesCS5A01G464500, TraesCS4B01G037400, TraesCS3D01G394200, TraesCS1D01G013000, TraesCS2B01G362700, TraesCS7D01G070900, TraesCS4D01G320600, TraesCS1A01G419600, TraesCS4B01G221900, TraesCS3A01G336700, TraesCS5D01G438300, TraesCS4D01G339800, TraesCS1D01G311200, TraesCS4B01G041200, TraesCS3D01G052500, TraesCS3A01G342900, TraesCS7D01G090300, TraesCS7D01G090600, TraesCS5D01G309000, TraesCS2A01G437100, TraesCS4D01G019300, TraesCS7B01G275200, TraesCS7B01G275000, TraesCS5B01G489600, TraesCS7D01G361200, TraesCS7D01G221100, TraesCS3D01G052600, TraesCS5D01G316400, TraesCS3D01G053500, TraesCS4B01G037500, TraesCS2B01G096300, TraesCS3D01G435900, TraesCS6A01G262300, TraesCS7D01G089500, TraesCS2A01G437500, TraesCS3B01G565200, TraesCS5D01G509100, TraesCS4D01G339400, TraesCS5A01G465100, TraesCS6D01G281100, TraesCS1D01G310000, TraesCS7A01G495400, TraesCS3A01G430100, TraesCS5A01G411500, TraesCS5A01G416300, TraesCS7B01G274500, TraesCS5B01G204800, TraesCS7A01G384400, TraesCS5A01G172700, TraesCS5D01G019600, TraesCS5D01G488900, TraesCS3D01G053400, TraesCS7A01G383900, TraesCS5A01G472000, TraesCS5A01G474300, TraesCS4B01G052300, TraesCS7D01G071200, TraesCS3A01G146600, TraesCS5D01G342400, TraesCS2A01G348500, TraesCS5D01G448200, TraesCS4B01G041100, TraesCS3D01G250600, TraesCS5A01G470200, TraesCS4A01G002900, TraesCS5A01G417000* |
| Ribosome | 32 | 363 | 0.004627491 | *TraesCS5D01G345600, TraesCS6D01G092500, TraesCS5D01G486500, TraesCS5A01G415800, TraesCS3D01G499500, TraesCS6A01G262600, TraesCS3D01G053200, TraesCS7A01G454700, TraesCS3A01G431800, TraesCS3A01G431700, TraesCS5A01G467200, TraesCS3D01G053100, TraesCS3D01G498500, TraesCS5D01G345700, TraesCS3A01G431600, TraesCS5A01G473700, TraesCS5D01G446300, TraesCS3A01G431300, TraesCS5A01G464200, TraesCS4D01G320800, TraesCS3A01G339200, TraesCS5A01G468600, TraesCS4D01G339500, TraesCS3A01G432000, TraesCS7B01G271400, TraesCS5A01G469300, TraesCS4D01G339700, TraesCS3D01G053000, TraesCS2B01G120400, TraesCS5A01G452400, TraesCS3A01G431500, TraesCS5D01G481100* |
| Biosynthesis of secondary metabolites | 69 | 1076 | 0.015545527 | *TraesCS1D01G310000, TraesCS7A01G495400, TraesCS4D01G339800, TraesCS2A01G425100, TraesCS5A01G417000, TraesCS4D01G339900, TraesCS4B01G041200, TraesCS5A01G454500, TraesCS1D01G013400, TraesCS7D01G090600, TraesCS2B01G362700, TraesCS7B01G275400, TraesCS5A01G465100, TraesCS5D01G316200, TraesCS1D01G013500, TraesCS7B01G275200, TraesCS5D01G309000, TraesCS5A01G416300, TraesCS4D01G019300, TraesCS2B01G276300, TraesCS5B01G204800, TraesCS7D01G090200, TraesCS5D01G488800, TraesCS6D01G330700, TraesCS4A01G002500, TraesCS5A01G177400, TraesCS5D01G342400, TraesCS2B01G101700, TraesCS4A01G043800, TraesCS3A01G337700, TraesCS7D01G090500, TraesCS7D01G089300, TraesCS5D01G509100, TraesCS5A01G476700, TraesCS5D01G316400, TraesCS2B01G401600, TraesCS3A01G338600, TraesCS5A01G472000, TraesCS1D01G013000, TraesCS7D01G221000, TraesCS5A01G465200, TraesCS2B01G096300, TraesCS4B01G052300, TraesCS7D01G071200, TraesCS5D01G488900, TraesCS3A01G451100, TraesCS7B01G275000, TraesCS5B01G489600, TraesCS2A01G348500, TraesCS5D01G448200, TraesCS5A01G475600, TraesCS3B01G565200, TraesCS4B01G041100, TraesCS3D01G250600, TraesCS7D01G221100, TraesCS7B01G250400, TraesCS2A01G437100, TraesCS4A01G002800, TraesCS5A01G411200, TraesCS7B01G274500, TraesCS3A01G342900, TraesCS4A01G002900, TraesCS7D01G090300, TraesCS1A01G419600, TraesCS4B01G221900, TraesCS7A01G382500, TraesCS5D01G438300, TraesCS7A01G001700, TraesCS7D01G089200* |
| Circadian rhythm-plant | 7 | 36 | 0.025481436 | *TraesCS4A01G122500, TraesCS4D01G019300, TraesCS2B01G365300, TraesCS4D01G052200, TraesCS7D01G089300, TraesCS5A01G154600, TraesCS4B01G052000* |

**Table S19: Summary of the KEGG enrichment analysis of genes located in selective regions during the improvement process.**

| **KEGG pathway** | **Group count** | **Total count** | **Corrected *P*-value** | **Genes** |
| --- | --- | --- | --- | --- |
| Monoterpenoid biosynthesis | 7 | 7 | 9.42784E-06 | *TraesCS2A01G371300, TraesCS2A01G371200, TraesCS2A01G371500, TraesCS2A01G371100, TraesCS2A01G370900, TraesCS2A01G371400, TraesCS2A01G371000* |
| Selenocompound metabolism | 6 | 18 | 0.001607668 | *TraesCS2B01G070300, TraesCS2A01G058800, TraesCS2B01G070000, TraesCS2B01G070100, TraesCS2B01G070200, TraesCS1B01G199600* |
| Biosynthesis of secondary metabolites | 56 | 1076 | 0.002258579 | *TraesCS2A01G058800, TraesCS2A01G381000, TraesCS6B01G276700, TraesCS7A01G322100, TraesCS2B01G070000, TraesCS2A01G381100, TraesCS2A01G371500, TraesCS4B01G144000, TraesCS2B01G070100, TraesCS6B01G280700, TraesCS2D01G005900, TraesCS2B01G070200, TraesCS2A01G371400, TraesCS4D01G000600, TraesCS6B01G284600, TraesCS2A01G384400, TraesCS7A01G227300, TraesCS2D01G064900, TraesCS7A01G254700, TraesCS6B01G284900, TraesCS7A01G226000, TraesCS2D01G081900, TraesCS7D01G500100, TraesCS2A01G370900, TraesCS7A01G324300, TraesCS1D01G420500, TraesCS2A01G382700, TraesCS5A01G407700, TraesCS7A01G222300, TraesCS2B01G070300, TraesCS7D01G498500, TraesCS6D01G369400, TraesCS7A01G222200, TraesCS3D01G087300, TraesCS2A01G380800, TraesCS2D01G007000, TraesCS2A01G371100, TraesCS7A01G319100, TraesCS7A01G219100, TraesCS1D01G419800, TraesCS2D01G076900, TraesCS2A01G371000, TraesCS1D01G042300, TraesCS3D01G090700, TraesCS2A01G371300, TraesCS3B01G125200, TraesCS7A01G473900, TraesCS2A01G371200, TraesCS2D01G010700, TraesCS7D01G498800, TraesCS2D01G080100, TraesCS2A01G380900, TraesCS3D01G086200, TraesCS7A01G232900, TraesCS6D01G369500, TraesCS2D01G006600* |
| Tryptophan metabolism | 8 | 46 | 0.003401917 | *TraesCS6B01G284600, TraesCS7D01G498100, TraesCS4A01G027500, TraesCS6B01G284900, TraesCS7D01G498000, TraesCS7D01G498300, TraesCS2D01G012100, TraesCS2D01G076900* |
| Metabolic pathways | 85 | 1910 | 0.005044203 | *TraesCS2A01G058800, TraesCS2A01G381000, TraesCS4A01G027500, TraesCS1A01G330000, TraesCS6B01G282800, TraesCS2D01G065100, TraesCS7A01G322100, TraesCS2B01G070000, TraesCS2A01G381100, TraesCS2D01G065200, TraesCS6B01G279100, TraesCS7A01G320000, TraesCS5A01G459000, TraesCS2B01G070100, TraesCS6B01G280700, TraesCS2D01G065300, TraesCS1D01G419400, TraesCS2D01G005900, TraesCS2B01G070200, TraesCS4D01G000600, TraesCS2A01G389900, TraesCS1D01G419800, TraesCS6B01G276700, TraesCS6B01G284600, TraesCS7A01G227300, TraesCS2D01G064900, TraesCS7A01G254700, TraesCS6B01G284900, TraesCS4B01G170800, TraesCS2D01G081900, TraesCS3D01G086200, TraesCS2D01G082000, TraesCS4B01G146800, TraesCS6B01G280400, TraesCS7D01G500100, TraesCS1A01G330200, TraesCS7A01G324300, TraesCS4A01G027800, TraesCS1D01G420500, TraesCS2A01G382700, TraesCS7A01G226000, TraesCS5A01G407700, TraesCS7A01G222300, TraesCS2B01G070300, TraesCS2D01G065500, TraesCS7D01G498500, TraesCS2A01G384400, TraesCS7A01G222200, TraesCS7A01G320400, TraesCS4B01G146900, TraesCS3D01G087300, TraesCS7A01G319000, TraesCS4B01G146600, TraesCS2A01G380800, TraesCS2D01G007000, TraesCS6B01G281700, TraesCS2D01G222800, TraesCS5A01G408100, TraesCS7A01G319100, TraesCS7A01G219100, TraesCS4B01G146700, TraesCS6B01G281600, TraesCS2D01G076900, TraesCS4B01G147000, TraesCS2D01G012100, TraesCS6B01G276300, TraesCS4B01G144000, TraesCS1B01G199700, TraesCS3D01G090700, TraesCS4B01G147300, TraesCS7D01G498800, TraesCS7A01G473900, TraesCS3B01G125200, TraesCS1D01G046100, TraesCS2D01G063100, TraesCS6B01G279400, TraesCS2D01G010700, TraesCS5A01G490500, TraesCS2D01G080100, TraesCS2A01G380900, TraesCS1A01G330900, TraesCS2D01G065400, TraesCS7A01G232900, TraesCS6D01G369500, TraesCS2D01G006600* |
| Cysteine and methionine metabolism | 12 | 112 | 0.005177038 | *TraesCS2B01G070300, TraesCS2A01G058800, TraesCS2B01G070000, TraesCS3D01G087300, TraesCS4A01G027800, TraesCS2B01G070100, TraesCS5A01G407700, TraesCS2B01G070200, TraesCS1B01G199600, TraesCS3B01G125200, TraesCS6B01G276700, TraesCS1D01G420500* |
| Phenylpropanoid biosynthesis | 13 | 157 | 0.018819218 | *TraesCS7A01G222300, TraesCS2A01G384400, TraesCS2A01G381000, TraesCS7A01G227300, TraesCS2D01G064900, TraesCS7A01G254700, TraesCS7A01G319100, TraesCS2A01G380800, TraesCS2D01G081900, TraesCS2A01G381100, TraesCS7A01G222200, TraesCS2A01G380900, TraesCS6D01G369500* |
| Carbon fixation in photosynthetic organisms | 8 | 69 | 0.022150196 | *TraesCS2D01G065500, TraesCS2D01G065100, TraesCS2D01G065200, TraesCS7A01G473900, TraesCS2D01G065300, TraesCS2D01G065400, TraesCS1D01G420500, TraesCS5A01G407700* |
| Sulfur metabolism | 6 | 41 | 0.027411071 | *TraesCS2B01G070300, TraesCS2A01G058800, TraesCS2B01G070000, TraesCS3D01G087300, TraesCS2B01G070100, TraesCS2B01G070200* |
| Glyoxylate and dicarboxylate metabolism | 8 | 74 | 0.031107711 | *TraesCS2D01G065500, TraesCS2D01G065100, TraesCS2D01G065200, TraesCS5A01G407700, TraesCS7A01G219100, TraesCS2D01G065400, TraesCS1D01G420500, TraesCS2D01G065300* |
