# Supplementary material for: Frequent intra- and inter-species introgression shapes the landscape of genetic variation in bread wheat
Source: Genome Biol. 2019 Jul 12;20:136. doi: 10.1186/s13059-019-1744-x (PMC6624984; doi:10.1186/s13059-019-1744-x)
Supplement: Supplementary file 16 — Review history. (DOCX 478 kb) [file 13059_2019_1744_MOESM16_ESM.docx]

Review History

**Comments to author and authors’ point-by-point responses to the reviewers’ comments:**

**Reviewer 1**

The report by Hong Cheng et al aimed to answer the question "where are the genetic diversities in modern common wheat from". They investigated the sequences data of 93 worldwide accessions, including wild emmer wheat, Ae. Tauschii, durum wheat, hexaploid landraces and hexaploid varieties. They managed to reveal the trajectories of nowadays wheat varieties and found that:1) D sub-genome has decreased genetic diversity than A and B sub-genome;2) a single northern Levant (NL) wild emmer origin of AB sub-genome of common wheat;3) introgressed fragments are the selection targets during wheat domestication. This work contains large amount of data and provides quite some deep insights into the understanding towards wheat domestication, although some of which had been more or less mentioned in other studies. It could be useful resource to people in the field. My major concerns are:

1.the authors combined the sequence information of self-generated 75 accessions and publicly available data. However, these datasets covers the genome with variable coverages, respectively, caused by different sequencing depth (eg.,8 x VS. ~50 x). In this case, how did the authors avoid bias since the deeper the genome was sequenced, the more SNP one would get. I would be curious to see the results obtained only using the 75 accessions.

*Response: We appreciated the reviewer’s constructive comment which is an important point we have missed. As commented, we performed SNPs statistics only using the 75 accessions that we have sequenced by ourselves. Compared with the previous results obtained from 93 accessions, the total number of SNPs reduced from 84,594,991 to 83,206,687 (reduced by only 1.6%) (as shown in the following Table 1). And, the population-specific and overlapped SNPs count among different populations showed the similar patterns as before (as shown in the following figures 1), suggesting there is no obvious bias on SNP calling using different set of accessions. Furthermore, we detected the relationship between the numbers of detected SNPs and the sequencing depth among hexaploid wheat accessions. It is true that more SNPs could be obtained with the increase of sequencing depth. However, the difference in number of detected SNPs between accessions with high (>10×) and low (<10×) sequencing depth is not significant (Figure 2).*

*In conclusion, the difference in sequencing depth between the downloaded samples and the samples we sequenced by ourselves in the study did not result in significant influence for detection of the variations.*

*Table 1. Comparison of the whole-genome SNPs identified from 93 and 75 accessions.*

| *subgenome* | *93 samples* | *75 samples* |
| --- | --- | --- |
| *A* | *34,802,951* | *34,707,846* |
| *B* | *33,300,925* | *33,212,635* |
| *D* | *16,491,115* | *15,286,206* |
| *Total* | *84,594,991* | *83,206,687* |

*Figure 1. The Venn diagram of the population-specific and overlapped SNPs among different populations. (a) for 93 accessions and (b) for 75 accessions.*

*Figure 2. The distribution of the number of SNPs of hexaploid wheat with different sequencing depth. Alter homozygous, the number of homozygous nonreference sites; Heterozygous, the number of heterozygous sites. The difference in number of detected SNPs between accessions with high (>10×) and low (<10×) sequencing depth is not significant. The indicated P values are based on one-way ANOVA.*

2.one very interesting finding is that introgressions are largely overlapped with QTLs and some special haploblocks, co-localizing with QTL loci were found. Would be essential to characterize at least one of these loci in more details, e.g., to pick up a candidate gene by analyzing more varieties (more haplotypes). This would largely increase the biological meaning of the study.

*Response: We appreciate the reviewer’s invaluable suggestion and comment. As suggested,* *we explored the introgression regions overlapped with the reported QTLs. About 9,600 genes were identified in these overlapped regions, including some important functionally known genes. The non-sense or shift mutations of these genes were examined between introgression and non-introgression accessions. Interestingly, we found a CAM7 gene (Calmodulin 7, TraesCS2B01G113800), which co-localized with a QTL associated with heading date and kernel weight [3, 4], contained a pre-mature stop mutation (the following Figure 3, or Additional file 1: Figure S34). The stop codon mutation was verified in 18 introgression accessions that we sequenced. CAM7 has been reported to act as a transcriptional regulator to enhance photomorphogenesis, and to regulate gene expression under various light conditions, as well as to regulate root growth and abscisic acid responses [1, 2].*

*Another case is TraesCS2B01G534200, which is the orthology gene of ATR2 in Arabidopsis thaliana) encoding an NADPH-cytochrome P450 reductase. A T-to-A mutation on the third exon in non-introgression accessions made the initial stop codon (TAG) in introgression accessions change to AAG (Lysine) (Figure 4, or Additional file 1: Figure S35), which was verified in 11 introgression accessions. This gene co-localized with the QTLs associated with leaf rust and plant height [3, 5]. In plants, ATR2 is found to be involved in altering tryptophan metabolism in plants [6]. Tryptophan, which is as an important precursor for auxin biosynthesis in plants, plays a vital role in disease resistance and plant growth. The mutation in this gene might be associated with disease resistance to leaf rust and plant height between introgression and non-introgression accessions.*

*The expression of TraesCS2B01G113800 and TraesCS2B01G534200 in introgression and non-introgression accessions were also detected and compared. It is no accident that TraesCS2B01G113800 displayed almost no expression in the introgression accessions but highly expressed in the non-introgression accessions (Figure 3). In addition, TraesCS2B01G534200 showed lower expression in introgression accessions than in non-introgression accessions (Figure 4).*

*Figure 3: Comparison of mapping statistics and gene expression of TraesCS2B01G113800 in intro (introgression) and non-intro (non-introgression) accessions. (a) The genomic sequencing mapping statistics visualized by Integrative Genomics Viewer (IGV). There were three consecutive mutations in the coding region in intro accessions, forming a stop codon that can result in premature termination of transcription. (b) The boxplot of gene expression between intro and non-intro accessions. The results showed that the expression (FPKM) of TraesCS2B01G113800 gene* *is nearly zero in intro accessions.*

*Figure 4: Comparison of mapping statistics and gene expression of TraesCS2B01G534200 in intro (introgression) and non-intro (non-introgression) accessions. (a) The genomic sequencing mapping statistics visualized by Integrative Genomics Viewer (IGV). A mutation on the third exon in non-intro accessions made the stop codon (TAG) in intro accessions change to AAG (Lysine). (b) The boxplot of gene expression between intro and non-intro accessions. The results showed that the expression (FPKM) of* *TraesCS2B01G534200 gene is obviously lower in intro accessions than non-intro accessions.*

3.Can the author also analyze the gene expression dynamics of the analyzed accessions with a focus on the introgressive fragments.

*Response: We appreciated the reviewer’s constructive suggestion.* *We performed* *RNA sequencing of 24 samples collecting from the roots and leaves from 3 wild emmer, 3 Ae. tauschii as well as 3 hexaploid landraces and 3 hexaploid varieties, which also have been re-sequenced.*

*Based on the obtained transcriptome data,* *we investigated the gene expression of 45 regions* *where at least two introgression accessions and two non-introgression accessions were sequenced in the 12 RNA-seq accessions, with 1373 genes in these regions.* *The genes that did express in all samples or the FPKM value in all samples lower than 1 were filtered in the analysis of differentially expressed genes (DEGs). Among the remaining 605 expressed genes, we identified* *18 DEGs (t-test, corrected P<0.05) between intro and non-introgression accessions (the following table 2, or Additional file 1: Table S10). Among them, 12 genes are up-regulated expression in non-introgression accession, while 6 genes showed higher* *expression in introgression accessions compared to non-introgression accessions.*

*Table 2. The list of differentially expressed genes (DEGs) between intro and non-intro accessions. The cells with yellow indicate introgression. In total, 18 DEGs (T-test, corrected P<0.05) between introgression and non-introgression accessions were identified, of which 12 genes are up-regulated expression in non-intro accession, while 6 genes showed higher expression in intro accessions compared to non-intro accession*

4.the manuscript is well-written but there is still some language issues. For instance, line 63, suspect/have two verbs in one sentence; line 113, in that?; line 433, of is missing? There are some more, please carefully check the writing in the revision.

*Response: We are sorry for language errors and other issues and we are grateful for your careful reviewing on our manuscript. We have revised the sentences at lines 63, 113 and 433 which you mentioned, and have amended language issues throughout the manuscript carefully. In the revised manuscript, we have tried to correct the mistakes and inappropriate writings. Additionally, the manuscript has been edited and proofread by native speaking co-authors to improve the quality. And we also consult the help of an English-language editing service, American Journal Experts (AJE), for professional editing.*

**Reviewer 2**

This is a very cool paper! It is a very thorough and deep analysis of the origins and evolution of the wheat sub genomes under >12,000 years of selection and hybridization. I don't feel qualified to judge the merits of every last statistical and bioinformatic test, but it looks like the conclusions and methodologies and generally sound.

1My principal major objection is that it is very difficult to read in parts due to the poor quality of the English - this requires thorough revision/rewriting.

*Response: Thank you very much for your suggestion. We have consulted the native English speakers to help us improve the quality of our manuscript. Also, the manuscript has been professionally edited for language by an English editing service agency, American Journal Experts (AJE).*

2.My second concern is that there is a lot of redundancy in the narrative in parts of the manuscript, for example the Introduction - it could be condensed significantly by eliminating unnecessary phrases and repeated ideas.

*Response: We appreciated your constructive suggestions. We have deleted these redundancy statements throughout our manuscript to make it concise and readable. All the changes are marked with red.*

3.A very minor concern is that the color scheme in some of the figures (ie, 3 and 4) could have been better if the authors had used more contrasting colors instead of brown, maroon, gold, and two different shades of blue. However, this is an aesthetic preference but note it might be difficult for red-green colorblind readers to discriminate between brown and maroon.

*Response: We apologize for the confusing colors of our figures. Following your suggestions, we have chosen more contrasting colors to improve figures 3, 4 as well as figure 5. In figure 3b and 5c, the indistinguishable colors brown, maroon and gold were all replaced by more contrasting colors red and light blue. And, the two different shades of blue in figure 3a were replaced by purple and green, respectively. All the revised figures have been provided in the revised version.*

*Reference*

*1.	Abbas, N., et al., Arabidopsis CAM7 and HY5 physically interact and directly bind to the HY5 promoter to regulate its expression and thereby promote photomorphogenesis. The Plant Cell, 2014. 26(3): p. 1036-1052.*

*2.	Abbas, N. and S. Chattopadhyay, CAM7 and HY5 genetically interact to regulate root growth and abscisic acid responses. Plant signaling & behavior, 2014. 9(9): p. e29763.*

*3.	Zhang, J., et al., Identification and validation of QTL for grain yield and plant water status under contrasting water treatments in fall-sown spring wheats. Theor Appl Genet, 2018.*

*4.	Ramya, P., et al., QTL mapping of 1000-kernel weight, kernel length, and kernel width in bread wheat (Triticum aestivum L.). Journal of applied genetics, 2010. 51(4): p. 421-429.*

*5.	Aoun, M., et al., Genome-wide association mapping of leaf rust response in a durum wheat worldwide germplasm collection. The plant genome, 2016. 9(3).*

*6.	Smolen, G.A., et al., Dominant alleles of the basic helix-loop-helix transcription factor ATR2 activate stress-responsive genes in Arabidopsis. Genetics, 2002. 161(3): p. 1235-1246.*
